# Cell-in-cell associated lncRNA signature predicts prognosis and immunotherapy response in gastric cancer

**DOI:** 10.3389/fonc.2025.1597187

**Published:** 2025-07-08

**Authors:** Junzuo Lin, Liancheng Wu, Zhengfei Zhao

**Affiliations:** ^1^ Department of Graduate School, Southwest Medical University, Luzhou, China; ^2^ Department of Gastrointestinal Surgery, The Affiliated Hospital of Southwest Medical University, Luzhou, China

**Keywords:** gastric cancer, cell-in-cell, long non-coding RNAs, tumor microenvironment, personalized therapy

## Abstract

**Introduction:**

Gastric cancer (GC) remains a leading cause of cancer mortality, necessitating robust prognostic biomarkers and personalized therapeutic strategies.

**Materials and methods:**

We developed a risk model integrating three cell-in-cell-associated lncRNAs (CICRlncRNAs: AP003392.1, AP000695.2, AL161785.1) using transcriptomic data from 367 TCGA-GC patients. The cohort was randomly split into training (n = 184) and test sets (n = 183) for model construction and external validation. Statistical rigor included LASSO-Cox regression, Kaplan-Meier analysis, and ROC curves assessing 1/3/5-year AUC.

**Results:**

The model stratified patients into low- and high-risk groups with distinct overall survival (OS, HR = 2.62, *P <*0.001) and progression-free survival (PFS, HR = 1.94, *P <*0.001). High-risk patients exhibited an immunosuppressive tumor microenvironment (TME), characterized by elevated Tregs (*P <*0.05) and M2 macrophages (*P <*0.05), correlating with poor response to immune checkpoint inhibitors (TIDE score, *P <*0.001). Drug sensitivity analysis revealed low-risk patients responded better to gefitinib/entinostat, while high-risk patients benefited from dasatinib/foretinib. Experimental validation confirmed AP000695.2 promoted proliferation and invasion in GC cells (*P <*0.01).

**Conclusion:**

This study establishes CICRlncRNAs as prognostic biomarkers and provides insights for precision therapy, though clinical applicability requires prospective validation.

## 1 Introduction

Gastric cancer (GC) refers to malignant tumors from the cardia to the pylorus and remains the second leading cause of cancer-related death worldwide. Despite reductions in incidence and mortality rates in several regions over the past half century, the five-year survival rate for patients with GC is only 20% (1). The prevalence and mortality of this disease vary widely across the world and between ethnic groups. Approximately 95% of cases of GC are pathologically classified as adenocarcinoma, which is mainly divided into the intestinal type and the diffuse type (1). The biological and clinical diversity of GC requires tailored therapeutic approaches, as a one-size-fits-all strategy leads to variable outcomes. The diversity in question encompasses a wide array of aspects, spanning from the genomic level to the environmental sphere. This complexity is manifested in the various subtypes of the condition. The primary basis for differentiating these subtypes lies in the molecular attributes of the cancer cells (2). A central element of therapeutic strategies for GC has become immune checkpoint inhibitors (ICIs). Pivotal clinical trials (e.g., CheckMate-649 for nivolumab and KEYNOTE-811 for pembrolizumab) have demonstrated that ICIs targeting PD-1/PD-L1, particularly in combination with chemotherapy, significantly improve survival outcomes in advanced GC patients (3, 4). However, prolonged use of ICIs can lead to the development of resistance to these drugs (5). Therefore, investigating the novel mechanisms underlying GC pathogenesis and identifying more effective therapeutic targets to ensure treatment efficacy are imperative.

The phenomenon of cell-in-cell (CIC) configurations is characterized by the occurrence of cells enclosed within the cytoplasm of other cells (6). This peculiar arrangement exerts various influences on the behavior and functionality of both the encapsulating and the enclosed cells, including aspects such as apoptosis, cell division and modulation of the immune system (7). CIC is particularly prevalent in different types of cancer tissues (8, 9). Research has suggested that CIC-mediated ‘in-cell killing’ could be a valuable approach in cancer immunotherapy (10). In addition, the potential clinical relevance of CIC has been recognized in the context of pancreatic cancer immunotherapy (11). Therefore, elucidating the mechanisms underlying CIC in tumors is crucial for gaining insight into the processes leading to cancer cell death.

In recent years, non-coding RNAs (ncRNAs) have received considerable attention for their role in the molecular pathways that contribute to cancer development (12). Research indicates that ncRNAs have the potential to influence the proper expression of associated genes, including proto-oncogenes and tumor suppressor genes. Consequently, they have emerged as promising targets for therapeutic intervention and potential biomarkers for the early detection of cancer (13). Among the class of ncRNAs, long non-coding RNAs (lncRNAs) have been particularly well studied in GC (14). For example, the lncRNA NEAT1 has been implicated in the pathogenesis of GC through multiple molecular pathways and has been associated with resistance to radiotherapy and chemotherapy as well as an unfavorable prognosis in GC patients. The observed correlation suggests that NEAT1 functions as an independent risk factor. Furthermore, it demonstrates considerable promise in terms of its potential applications in clinical therapy and as a prognostic marker (15). In addition, lncRNAs associated with ferroptosis, telomeres, and the immune response have been used to predict survival in GC patients (16–18). These studies provide valuable insights for therapeutic strategies. Notwithstanding the documented involvement of a multitude of lncRNAs in GC, further in-depth research on this topic is warranted. However, lncRNAs specifically regulating cell-in-cell (CIC) structures—a phenomenon linked to immunosuppressive TME remodeling and therapy resistance (7, 19)—remain largely unexplored. Elucidating CIC-specific lncRNAs (CICRlncRNAs) may reveal unique mechanisms distinct from canonical lncRNAs (e.g., NEAT1 or ferroptosis-related lncRNAs), given their potential to modulate both cell-cell engulfment processes and immune evasion pathways. This necessity arises from the limited availability of clinical samples and the inherent constraints of existing cellular and animal models.

The efficacy of ICIs is intrinsically tied to the tumor microenvironment (TME), where CIC structures have emerged as key regulators of immune suppression. Specifically, GC is increasingly being treated with immunotherapy, which has become a conventional approach. Recent research highlights the critical role of the tumor microenvironment (TME) in determining the immunotherapy response. For instance, CIC structures within the TME may promote immune evasion by modulating macrophage polarization (e.g., M2 macrophage enrichment) and regulatory T cell (Treg) infiltration (19). This aligns with the observed failure of ICIs in approximately 66% of advanced GC patients, where an immunosuppressive TME is a key barrier (20). Consequently, immunotherapy strategies have shifted from directly targeting tumor cells to reprogramming the TME through immune checkpoint inhibition and stromal modulation. Immunotherapy strategies using ICIs, including those targeting PD-1/PD-L1 and CTLA-4, focus primarily on modulating the TME (21). These therapies have transformed the treatment of a wide range of cancers (20). A recent review suggested that inhibitors of the PD-1/PD-L1 pathway have therapeutic activity in the treatment of advanced GC, particularly at later stages of the disease. However, the benefit of using these inhibitors as the sole treatment modality is relatively modest (22). Pretreatment with chemotherapy in GC has been shown to significantly alter the immunological landscape within the TME. Specifically, this treatment approach has been associated with a decrease in regulatory T cells (Tregs) and a concomitant increase in cytotoxic CD8^+^ T cells within the TME. These changes are thought to contribute to the remodeling of the TME in GC, thereby improving the clinical outcomes of affected individuals (23).

We hypothesized that CICRlncRNAs constitute a novel class of biomarkers capable of predicting GC prognosis and immunotherapy response by orchestrating CIC-driven TME immunosuppression. To test this, we aimed to (1) a CICRlncRNA-based risk model for prognostic stratification; (2) decipher the functional roles of CICRlncRNAs in TME modulation and immune evasion; and (3) identify risk-group-specific therapeutic vulnerabilities, including ICI responsiveness and targeted drug sensitivity.

To increase the accuracy of predicting the prognosis of GC patients, this research drew upon clinical data related to GC patients sourced from The Cancer Genome Atlas (TCGA) database. R software was subsequently used to construct a prognostic model, which was grounded in the varied expression levels of CICRlncRNAs. The research also investigated the biological roles of CICRlncRNAs, associated pathways, immune cell infiltration, somatic mutation profiles and responses to immunotherapy while evaluating the impact of antitumor drugs on patients in different risk categories. These results not only deepen our understanding of the mechanisms by which CICRlncRNAs influence GC but also provide potential guidance for the clinical prognostic management of patients with GC.

## 2 Materials and methods

### 2.1 Data collection

RNA-seq, clinical and somatic mutation data from GC patients were obtained from the TCGA database. This included transcriptomic information from 412 tumor samples, 36 normal tissue samples and 443 clinical records. The GC patients were then randomly divided into a training set (n = 184) and a test set (n = 183). To assess the disparities in clinical features between the two patient groups, the chi-square test was utilized. Concurrently, genes associated with cell-in-cell phenomena were extracted from a recently released research study (24).

### 2.2 Construction of a risk model based on CICRlncRNAs

A comprehensive review of the relevant literature was conducted, leading to the identification of 101 genes associated with intracellular processes. An assessment of the relationships between lncRNAs and the expression profiles of these intracellular-related genes was subsequently performed, utilizing Pearson correlation coefficients with thresholds of *P <*0.001 and |R| >0.4. A stepwise reduction process was subsequently employed to identify CICRlncRNAs with prognostic significance.

In the initial phase of the study, 18 CICRlncRNAs were identified as being associated with the prognosis of GC patients through univariate Cox regression analysis. LASSO (Least Absolute Shrinkage and Selection Operator) Cox regression analysis was then performed using the ‘glmnet’ R package. The optimal penalty parameter (λ) was determined via 10-fold cross-validation, resulting in λ.min = 0.0338 and λ.1se = 0.0856. To achieve a more parsimonious model with stronger generalization potential, the λ.1se criterion was selected. Applying this λ value, subsequent dimensionality reduction via the lasso algorithm revealed that a subset of 3 CICRlncRNAs was associated with the OS of GC patients. In conclusion, a multivariate Cox regression analysis was performed. This analysis successfully identified these three CICRlncRNAs (AP003392.1, AP000695.2, and AL161785.1) as crucial indicators of prognosis. Following the discovery of these results, a prognostic model was developed. The risk score for each patient was ascertained via a formula generated by the model. Following this computation, the patients were divided into two distinct risk categories, with the division being based on the median value of the risk scores.

### 2.3 Assessing the accuracy and independence of the risk model and constructing nomograms

To evaluate the predictive capacity of the risk model, Kaplan–Meier (K–M) curves were generated. To further evaluate the model’s predictive ability, receiver operating characteristic (ROC) curves were generated. Specifically, the model’s performance in forecasting survival was assessed at 1-year, 3-year, and 5-year intervals. In addition, principal component analysis (PCA) was performed for two objectives: (i) to visualize intrinsic clustering patterns of CICRlncRNAs across subgroups and (ii) to validate the risk model’s stratification capability by projecting risk scores into reduced-dimensional space. The reliability of the risk model as a predictor of outcomes was further examined through both univariate and multivariate Cox regression analyses. Furthermore, a nomogram was constructed by synthesizing multiple clinical features along with the corresponding risk scores. Calibration curves were subsequently constructed to assess the predictive accuracy of the nomogram.

### 2.4 Functional enrichment analysis

DEGs were identified using the limma R package with thresholds of |log2FC| >1.0 and nominal *P <*0.05, followed by Benjamini-Hochberg FDR correction (*P <*0.05) to control false discoveries. To further understand the biological roles of the DEGs, a Gene Ontology (GO) analysis was conducted. The GO analysis encompassed three primary aspects: biological processes (BPs), cellular components (CCs), and molecular functions (MFs). Furthermore, Kyoto Encyclopedia of Genes and Genomes (KEGG) analysis was employed to investigate the signaling pathways related to these DEGs. In conclusion, gene set enrichment analysis (GSEA) was carried out to characterize differential signaling pathways within the low- and high-risk groups.

### 2.5 Tumor microenvironment, analyzing somatic mutations and predicting drug response

The ESTIMATE algorithm was used to assess differences in the TME between the two risk groups (25). To ascertain the infiltration degrees of 22 distinct immune cell types within these groups, the CIBERSORT algorithm was employed, as referenced in the literature (26). Furthermore, the ssGSEA algorithm was utilized to explore immune cell infiltration and immune function across various risk categories (27). Tumor immune dysfunction and exclusion (TIDE) scores were calculated to assess the immune escape of tumor cells and their responsiveness to ICIs (28). The TIDE algorithm has been extensively validated in gastric cancer cohorts for predicting ICI response. For instance, studies have confirmed its prognostic utility in GC patients treated with anti-PD-1/PD-L1 therapy (29, 30). Somatic mutation data from the TCGA database were analyzed to determine the tumor mutational burden (TMB) of patients in the two risk groups, with patients categorized into low and high TMB groups on the basis of the median TMB score. In addition, the R package ‘oncoPredict’ was used to calculate the IC50 values of common antitumor drugs using the GDSC2 (Genomics of Drug Sensitivity in Cancer, version 2) reference dataset to predict the drug response of GC patients in different risk groups. The GDSC2 database provides drug sensitivity profile (IC50) and transcriptomic data from over 1,000 cancer cell lines, enabling robust prediction of patient-specific drug responses.

### 2.6 Cellular cultivation techniques coupled with quantitative reverse transcription polymerase chain reaction methodologies

The GC cell lines AGS and HGC-27, along with the normal control cell line GES-1, were procured from Procell located in China. These cell lines were maintained in RPMI-1640 medium, which was supplied by Gibco (USA). The culture medium was supplemented with 10% fetal bovine serum sourced from Gibco, USA, as well as a 1% mixture containing streptomycin and penicillin. The culture conditions were a humidified environment at 37°C with 5% CO_2_. Total RNA was extracted via TRIzol (Invitrogen, USA), and CICR lncRNA expression was measured via qRT–PCR. PCR amplification was performed on the ABI 7500 platform utilizing UltraSYBR (CWBIO, China) following standard protocols. In this process, GAPDH served as the internal control to ensure the reliability and accuracy of the experimental results. While multi-reference genes (e.g., β-actin and 18S rRNA) are sometimes recommended, GAPDH alone has been widely adopted as a stable control in GC transcriptomic studies due to its consistent expression across gastric tissue types (16, 31, 32). Notably, qRT–PCR validation of AL161785.1 was not feasible because of the lack of specific primer sequences for the lncRNA AL161785.1 in public databases such as NCBI and Ensembl and unsuccessful attempts at online primer design using tools such as Primer-BLAST. The primer sequences utilized in the PCR are enumerated in **Table 1**.

**Table 1:** Primer sequences for qRT–PCR.

Primer name	Primer sequence (5’−3’)
AP000695.2	F: 5’ GGACACTCTGAAGGAACTC 3’
	R: 5’ GATGACCATTAGCCAACAAG 3’
AP003392.1	F: 5’ GAATTCACCCACCTCAGCC 3’
	R: 5’ GTGTGCGTTTTCCCACTGTC 3’
GAPDH	F: 5’ CCCCACCACACTGAATCTCC 3’
	R: 5’ GTACATGACAAGGTGCGGCT 3’

### 2.7 Cell transfection assay

To investigate the functional role of AP000695.2 in gastric cancer progression, siRNA-mediated gene silencing was performed. Custom-designed siRNA duplexes targeting AP000695.2 (GenBank accession: NR_135734.1). AGS gastric cancer cells were seeded in 6-well plates at a density of 5 × 10^5^ cells/well and cultured until reaching 60–70% confluence. Transfection complexes were prepared according to the manufacturer’s protocol using Lipofectamine™ 3000 (Invitrogen, USA). Briefly, 100 pmol of siRNA and 5 μL of Lipofectamine 3000 were mixed in 250 μL of serum-free Opti-MEM medium (Gibco, USA), incubated at room temperature for 15 min, and then added to cell cultures. After 6 h of transfection, the medium was replaced with fresh complete DMEM containing 10% fetal bovine serum (FBS), followed by 24 h of additional incubation prior to functional assays. Knockdown efficiency was confirmed via qRT–PCR 24 h post-transfection using GAPDH for normalization, with primers listed in **Table 1**.

### 2.8 Cell proliferation analysis

Cell proliferation capacity was evaluated using the CCK-8 assay (TargetMol, Catalog# TP1197) to determine the biological effects of AP000695.2 knockdown in gastric cancer cells. At 24 h post-transfection, the cells were harvested and seeded into 96-well plates at 3 × 10³ cells/well with 100 μL of RPMI-1640 medium supplemented with 10% FBS. To minimize proliferation-dependent confounding, measurements at early timepoints (24 h) primarily reflect metabolic activity rather than proliferation rates (33). Optical density measurements were performed at 0, 24, 48, and 72 h intervals using the following protocol: 10 μL of CCK-8 reagent was added to each well, followed by a 2 h incubation at 37°C under standard culture conditions. The absorbance values were quantified using a microplate reader (BioTek Instruments, USA) with dual-wavelength detection (450 nm measurement wavelength vs. 650 nm reference wavelength). Three technical replicates were included per experimental group, with all the assays independently repeated in triplicate.

### 2.9 Wound healing assay

To assess the impact of AP000695.2 knockdown on gastric cancer cell migration dynamics, a standardized wound healing assay was implemented. Twenty-four hours post-transfection, AGS cells were seeded into 6-well plates at 2 × 10^6^ cells/well and cultured to form confluent monolayers. Mechanical wounds were generated using 200 μL sterile pipette tips, with three parallel scratches created perpendicular to the plate surface. The detached cells were removed by gentle washing with PBS, followed by incubation with serum-reduced medium (Gibco, USA) containing 1% FBS to minimize proliferation interference. Wound closure was documented at 0 and 24 h post-scratching through systematic image acquisition of five random fields per well under phase-contrast microscopy (Olympus, Japan) at 40× magnification. Quantitative analysis was performed using ImageJ software (National Institutes of Health, USA) by calculating the relative wound area reduction: **Migration rate (%)=(1−A_initial_/A_terminal​_​)×100%,** where A_initial_ and A_terminal_ represent the wound areas at baseline and post-migration timepoints, respectively. Triplicate experiments with three biological replicates ensured statistical robustness.

### 2.10 Transwell migration and invasion assay

The cell migration capacity was evaluated using 8 μm pore Transwell chambers (Corning, USA). A Transwell chamber system was employed to evaluate the cellular migration capacity. Transfected AGS cell suspensions (1×10^4^ cells/chamber) in serum-free conditions were plated in the upper chambers, with complete growth medium containing 10% fetal bovine serum serving as a chemoattractant in the lower compartments (600 μL volume). After 24 hours of incubation under standard culture conditions (37°C, 5% CO_2_), residual non-migratory cells retained on the upper chamber surface were mechanically eliminated using sterile cotton applicators. The transmembrane migratory population was subsequently processed through sequential steps: primary fixation with 4% paraformaldehyde solution, followed by 0.1% crystal violet histochemical staining. Given that the assay duration (24 h) is significantly shorter than the cell doubling time (AGS: ~32 h), the observed differences predominantly reflect migration/invasion capacity rather than proliferation effects.

Quantitative analysis was performed through microscopic enumeration of stained cellular elements adhering to the lower membrane surface. For invasion assessment, Transwell inserts were pre-coated with 50 μL of Matrigel matrix (Corning, USA) diluted 1:8 in serum-free medium and polymerized at 37°C for 1 h prior to cell seeding. Subsequent processing mirrored the migration protocol. Cell quantification was performed by imaging five random fields per chamber under 200× magnification (Nikon Eclipse, Japan). Stained cells within each field were manually counted using ImageJ software (National Institutes of Health, USA). The results from three independent experiments (each with three technical replicates) are expressed as the mean ± standard deviation (SD) of the number of cells per field. The relative migration/invasion rate was calculated as follows: (mean cell count of the experimental group/mean cell count of the control group) × 100%.

### 2.11 Statistical analysis

In this study, R software (version 4.3.3) was employed to conduct all the statistical analyses. For comparing different groups, the t test was utilized. To assess the differences in survival rates across distinct risk strata, the K–M method coupled with the log-rank test was employed. A series of univariate and multivariate Cox regression analyses were subsequently systematically conducted with the objective of pinpointing the factors associated with the prognosis of patients with GC. It was determined that a *P <*0.05 would indicate statistical significance.

## 3 Results

### 3.1 Data of patients with GC

**Figure 1:**
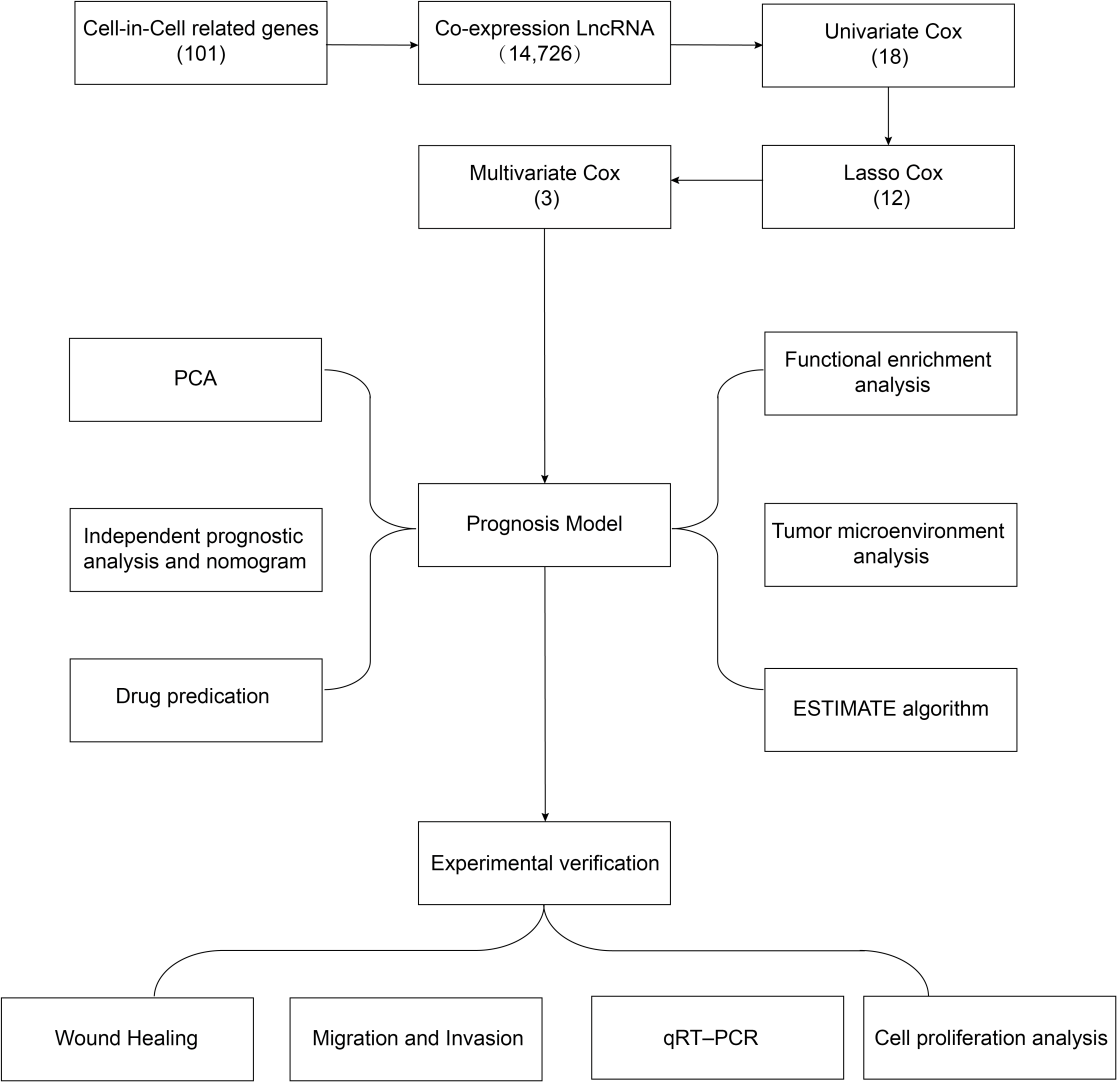
Flow chart of the study protocol.

A flowchart of the study design is shown in **Figure 1**. In this study, 367 GC patients were recruited and randomly assigned to a training group of 184 patients and a test group of 183 patients at an approximate 1:1 ratio. Data from the training cohort were used to screen for prognostically relevant CICRlncRNAs and to develop a prognostic model. In contrast, the accuracy of the model was assessed via data derived from the test group. **Table 2** shows that there were no statistically significant disparities in clinical features, including age, gender, grade, and TNM staging between the two groups (*P >*0.05).

**Table 2:** The clinical characteristics of the GC patients in the training, validation and overall sets.

	Overall	Validation	Training	*P*
Age				0.8157
<60	106(32.42%)	52(31.52%)	54(33.33%)	
≥60	221(67.58%)	113(68.48%)	108(66.67%)	
Gender				0.415
FEMALE	119(36.39%)	56(33.94%)	63(38.89%)	
MALE	208(63.61%)	109(66.06%)	99(61.11%)	
Grade				0.2669
G1	8(2.45%)	3(1.82%)	5(3.09%)	
G2	110(33.64%)	62(37.58%)	48(29.63%)	
G3	209(63.91%)	100(60.61%)	109(67.28%)	
Stage				0.7404
Stage I	42(12.84%)	21(12.73%)	21(12.96%)	
Stage II	108(33.03%)	52(31.52%)	56(34.57%)	
Stage III	145(44.34%)	73(44.24%)	72(44.44%)	
Stage IV	32(9.79%)	19(11.52%)	13(8.02%)	
T classification				0.8004
T1	15(4.59%)	8(4.85%)	7(4.32%)	
T2	68(20.8%)	37(22.42%)	31(19.14%)	
T3	156(47.71%)	79(47.88%)	77(47.53%)	
T4	88(26.91%)	41(24.85%)	47(29.01%)	
N classification				0.5766
N0	102(31.19%)	46(27.88%)	56(34.57%)	
N1	89(27.22%)	47(28.48%)	42(25.93%)	
N2	67(20.49%)	37(22.42%)	30(18.52%)	
N3	69(21.1%)	35(21.21%)	34(20.99%)	
M classification				0.5157
M0	307(93.88%)	153(92.73%)	154(95.06%)	
M1	20(6.12%)	12(7.27%)	8(4.94%)	

### 3.2 GC prognosis risk model based on CICRlncRNAs

**Figure 2:**
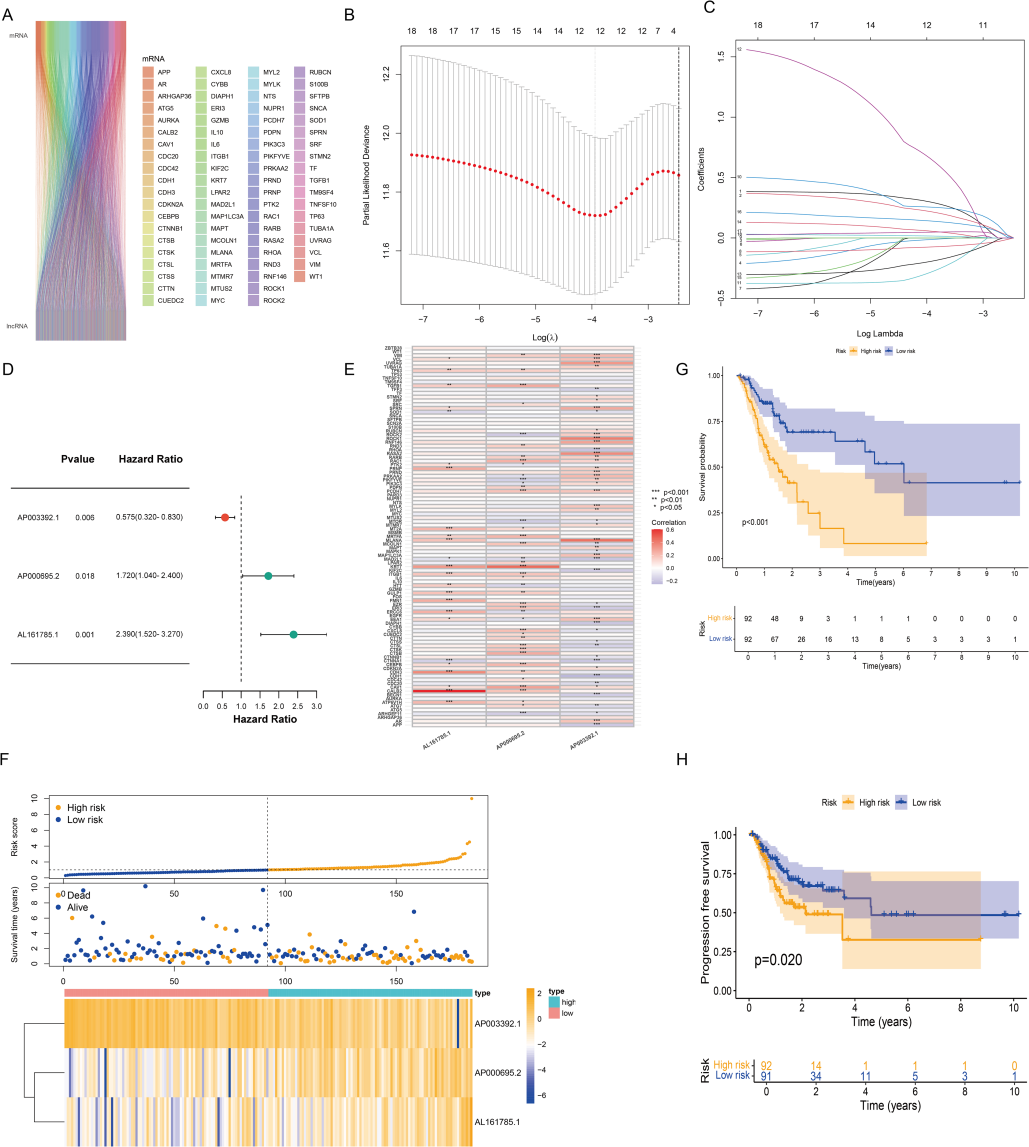
Construction of the risk model based on CICRlncRNAs. **(A)** Sankey diagram demonstrating CIC-related genes and CICRlncRNAs. **(B, C)** Eighteen CICRlncRNAs were identified via LASSO regression analysis. **(D)** Three CICRlncRNAs were used to construct the multivariate Cox regression model. **(E)** Correlation heatmap of 3 CICRlncRNAs and 101 CIC-related genes included in the multivariate Cox regression model. *P <0.05, **P <0.01, ***P <0.001. Red colours indicate positive correlation, blue colours indicate negative correlation. **(F)** Distribution of risk scores, survival status and survival time patterns of patients in different risk groups in the training set and the expression heatmap of the 3 CICRlncRNAs. OS **(G)** and PFS **(H)** of patients in different risk groups in the training set.

We retrieved 101 CIC-related genes from the literature. By employing the Pearson correlation coefficient method, we analyzed the correlation between lncRNAs and CIC-related mRNAs within the TCGA database. Ultimately, 684 CICRlncRNAs were filtered out (**Supplementary Table S1**). As shown in **Figure 2A**, the Sankey diagram illustrates the coexpression network between 101 CIC-related genes and 684 CICRlncRNAs. Univariate Cox regression analysis was performed on the OS data of the GC patients in the training set. This analysis revealed that 18 CICRlncRNAs were associated with the OS of GC patients (**Supplementary Table S2**). LASSO Cox regression analysis with 10-fold cross-validation was performed (**Figures 2B, C**). Applying the λ.1se criterion (λ = 0.0856) for model parsimony, a subset of 3 key lncRNAs closely associated with the prognosis of GC patients was identified. Finally, three prognostically relevant lncRNAs were selected, namely, AP003392.1, AP000695.2 and AL161785.1. A multivariate Cox regression model was then constructed (**Figure 2D**). The risk score was calculated by multiplying the expression levels of specific lncRNAs by their respective Cox regression coefficients, as described below: risk score value = AP003392.1 × (-0.668208656275531) + AP000695.2 × (0.393189166858392) + AL161785.1 × (0.80085986373646). Furthermore, the interactions between 101 CIC-associated genes and 3 CIC-related lncRNAs were examined (**Figure 2E**). **Figure 2F** shows the expression levels of these three CIC-related lncRNAs in the low-risk and high-risk groups. To assess differences in overall survival and progression-free survival (PFS) between the two groups, K–M curves were generated. The results revealed that individuals in the low-risk group had significantly better overall survival and PFS than those in the high-risk group did (*P <*0.001 for all groups) (**Figures 2G, H**).

### 3.3 Validating the CICRlncRNA-based risk model and PCA

**Figure 3:**
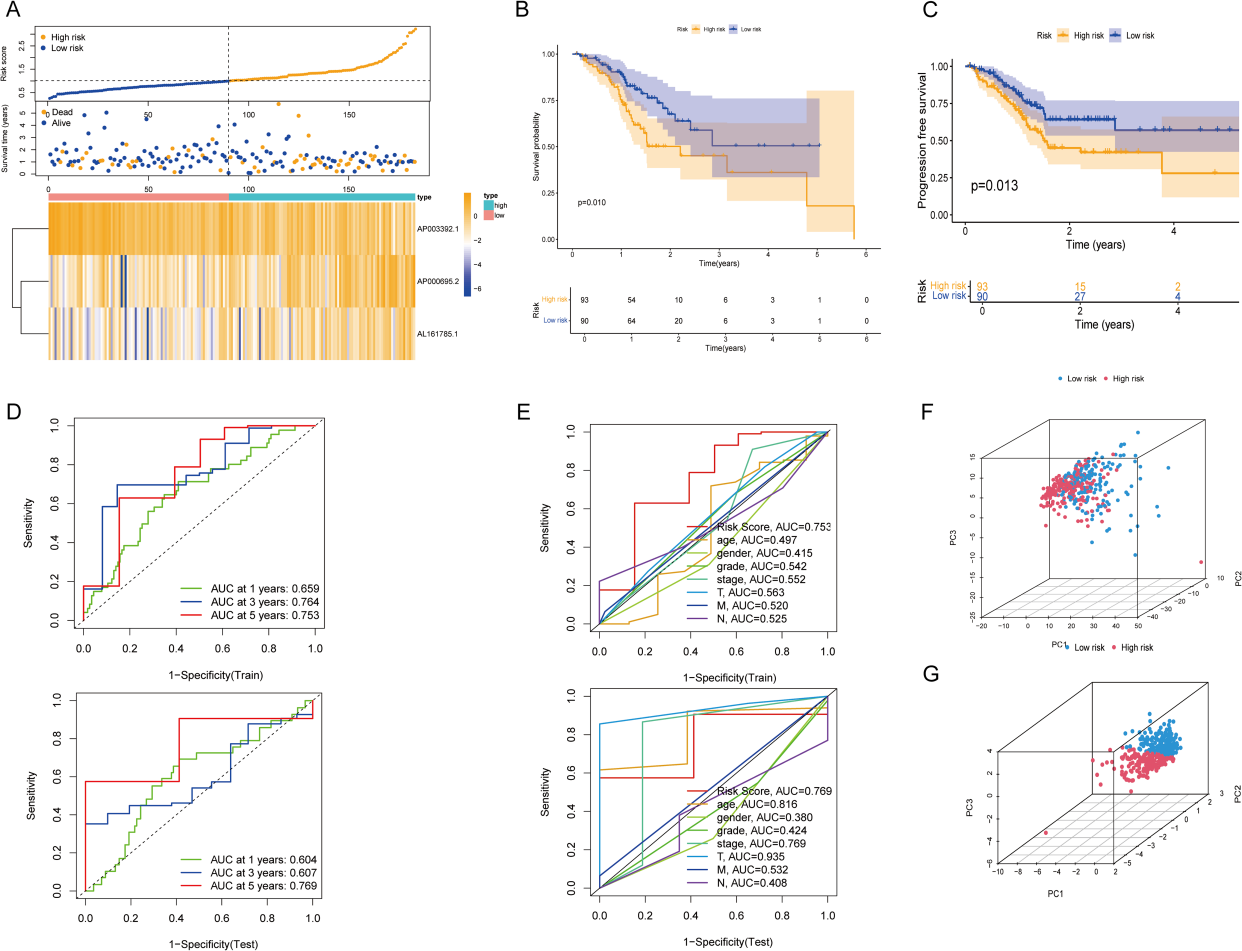
Validation of the CICRlncRNA-based risk model and PCA. **(A)** Risk score distribution, survival status and survival time patterns of patients in different risk groups in the test set and the expression heatmap of 3 CICRlncRNAs. **(B, C)** Kaplan-Meier curves for OS and PFS between low-risk and high-risk groups. **(D)** ROC curves for predicting 1-, 3- and 5-year OS in training and test sets. **(E)** ROC curves of the clinical risk indicators and risk scores in training and test sets. **(F)** PCA between different risk groups based on CICRlncRNAs. **(G)** PCA between different risk groups based on the risk model.

The reliability of the risk model was verified in the test set. As shown by the risk curves and scatter plots, when comparing scores, patients in the low-risk group were positioned relatively lower than those in the high-risk group (**Figure 3A**). Furthermore, the heatmap confirmed that AP003392.1 acted as a protective factor for GC, whereas AP000695.2 and AL161785.1 were identified as risk factors. K–M analysis revealed that the low-risk group had longer OS (*P* = 0.010) and PFS (*P* = 0.013) than did the high-risk group (**Figures 3B, C**). In both the training and test datasets, the risk model’s area under the curve (AUC) for predicting 1-year, 3-year, and 5-year OS exceeded 0.6 (**Figure 3D**). ROC curves were used to assess the sensitivity and specificity of the risk scores for the test set. The AUCs of the risk scores in both the training and test sets were greater than those of the other clinical risk indicators (**Figure 3E**). PCA based on CICRlncRNAs expression (**Figure 3F**) revealed partial subgroup separation, indicating that these lncRNAs capture biological heterogeneity. Critically, PCA of the risk scores (**Figure 3G**) revealed clear divergence between the low- and high-risk groups (PC1 contribution: 35.7%), confirming the model’s stratification robustness.

### 3.4 Independent analysis of the prognosis and the nomogram

**Figure 4:**
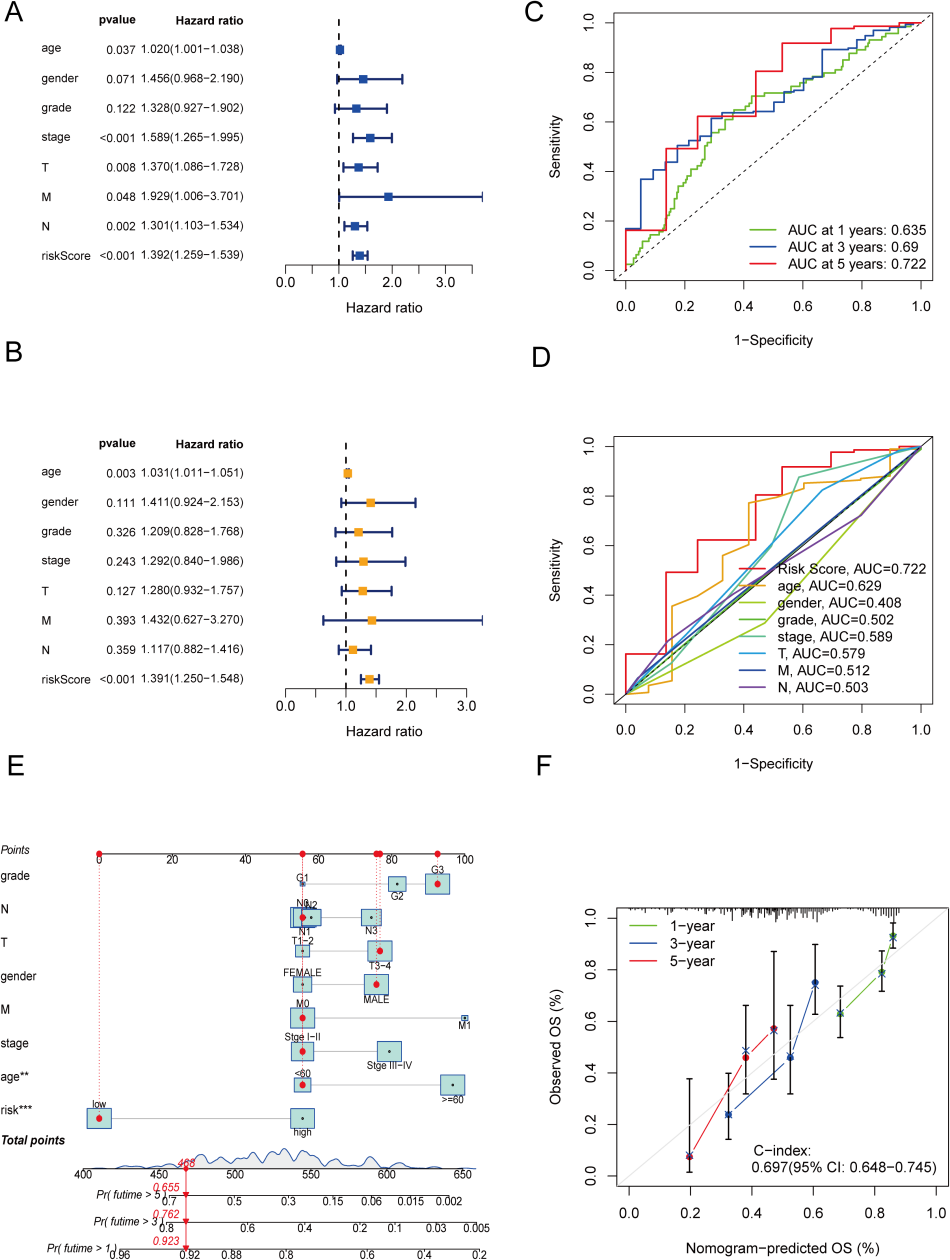
Independent prognostic analysis and construction of a nomogram. **(A)** Forest plot of univariate Cox regression analysis. **(B)** Forest plot of multivariate Cox regression analysis. **(C)** ROC curves of clinical risk indicators and risk scores for the entire cohort. **(D)** ROC curves of the risk model for predicting 1-, 3- and 5-year OS for the entire cohort. **(E)** Nomogram. **(F)** Calibration curves of the nomogram.

**Figure 5:**
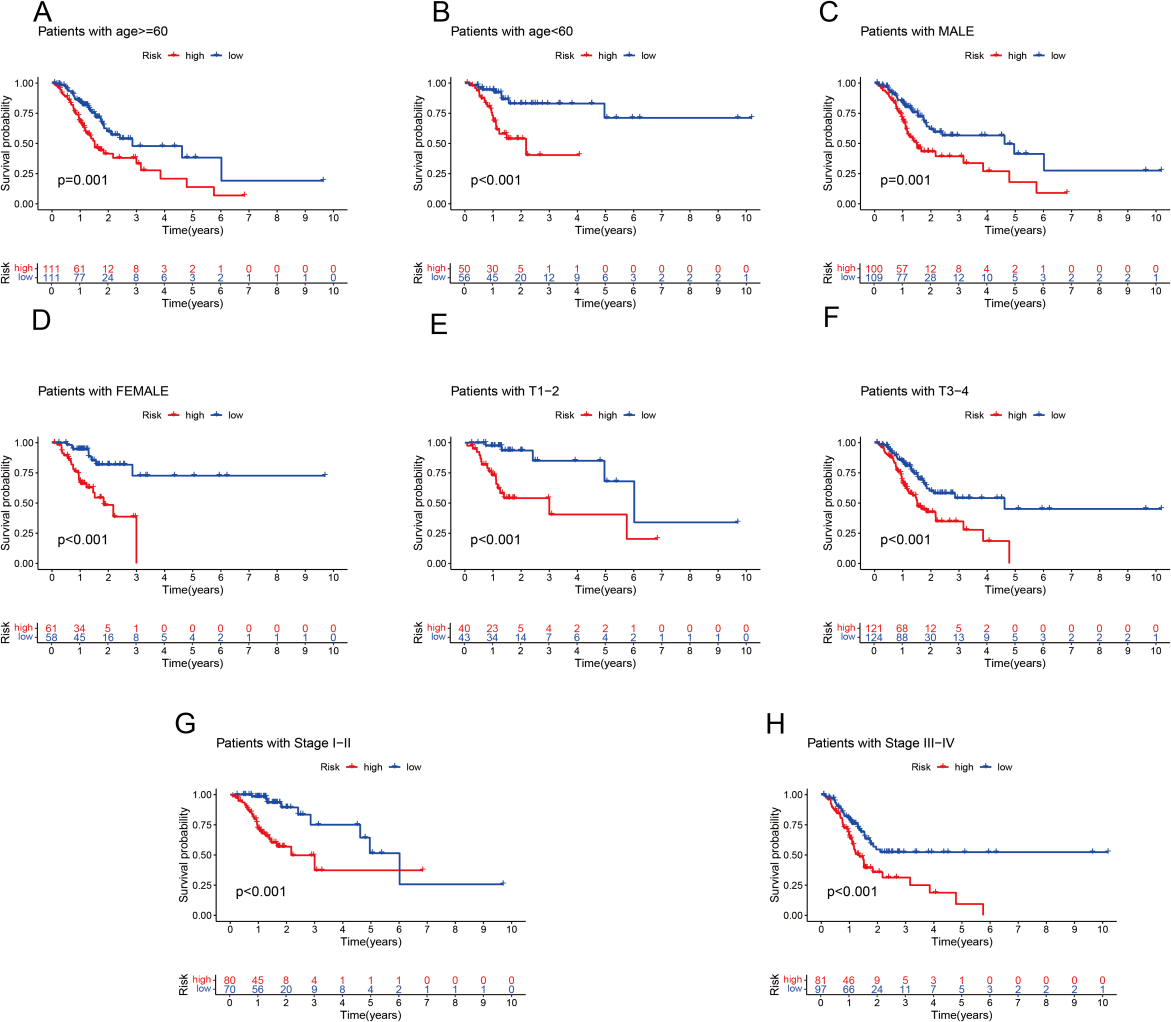
K–M analysis of OS in different subgroups based on the clinical characteristics of patients with GC in the TCGA cohort. **(A)** Age ≤ 60 years. **(B)** Age > 60 years. **(C)** Male. **(D)** Female. **(E)** T1–T2. **(F)** T3–T4. **(G)** Stages I–II. **(H)** Stage III–IV.

To independently assess the predictive power of the three CICRlncRNAs for OS in GC patients, we executed Cox proportional hazards regression analyses, encompassing both single-variable and multiple-variable approaches. Our findings from these analyses revealed that the calculated risk score served as a significant and independent prognostic indicator for OS in the context of GC, with a *p* value <0.001 (**Figures 4A, B**). The AUC of the risk model for predicting 1-, 3- and 5-year survival probabilities within the entire cohort was greater than 0.6. This demonstrated the predictive accuracy and reliability of the model (**Figure 4C**). In addition, the risk model had higher AUC values for the entire cohort (AUC = 0.722) than the other clinical risk indicators did (**Figure 4D**). A nomogram was created by incorporating multiple clinical factors and risk scores to predict the 1-, 3- and 5-year survival of patients with GC (**Figure 4E**). A calibration curve was then plotted to validate the accuracy of the nomogram (**Figure 4F**). K–M curves were plotted to assess the efficiency of the risk model in predicting various clinical characteristics, including age, gender and TNM stage. The results revealed that the low-risk group had a more favorable prognosis (**Figure 5**).

### 3.5 Analysis of functional enrichment

**Figure 6:**
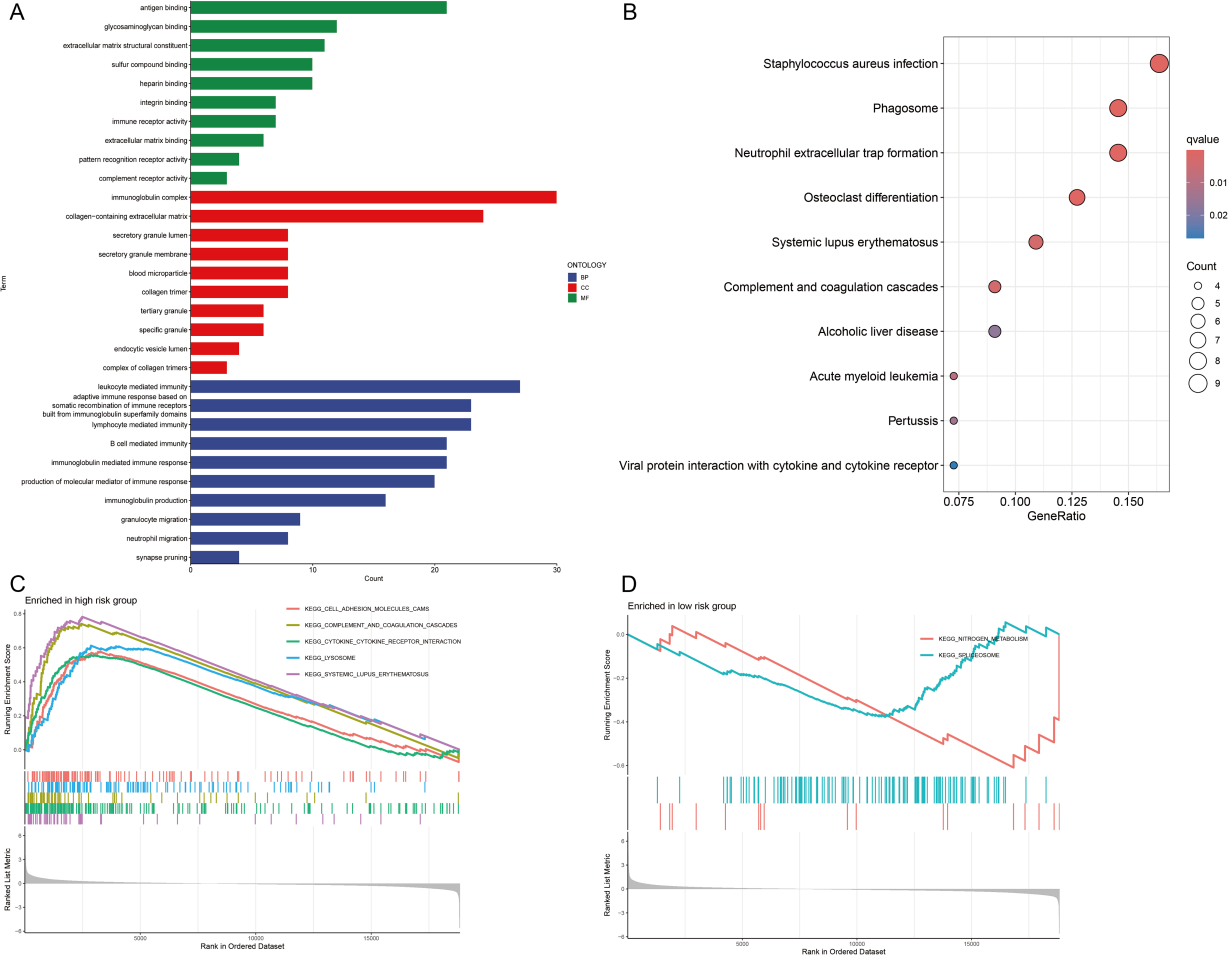
Functional enrichment analyses. **(A)** Gene Ontology (GO) analysis of biological processes (BPs), cellular components (CCs), and molecular functions (MFs). **(B)** Kyoto Encyclopedia of Genes and Genomes (KEGG) pathway analysis. **(C, D)** Gene Set Enrichment Analysis (GSEA) of signaling pathways in high- and low-risk groups.

GO and KEGG enrichment analyses were carried out to clarify the functions of the 183 DEGs (**Supplementary Table S3**). These DEGs were enriched predominantly in immune-related BPs. The immune system comprises several key components, such as leukocyte-mediated immunity and lymphocyte-mediated immunity. Additionally, it encompasses an adaptive immune response that is dependent on the somatic recombination of immune receptors, which are composed of immunoglobulin superfamily domains. In CCs, the DEGs were strongly enriched in the immunoglobulin complex and the extracellular matrix containing collagen. For MFs, DEGs were involved in antigen binding, glycosaminoglycan binding and extracellular matrix structural components (**Figure 6A**). KEGG enrichment analysis revealed that the DEGs were predominantly enriched in pathways related to *Staphylococcus aureus* infection and phagosome and neutrophil extracellular trap formation (**Figure 6B**).

Furthermore, GSEA revealed that pathways related to cell adhesion molecules, complement and coagulation cascades, cytokine–cytokine receptor interactions, lysosomal function and systemic lupus erythematosus were significantly enriched in the high-risk group (**Figure 6C**). Conversely, pathways related to nitrogen metabolism and the spliceosome were significantly enriched in the low-risk group (**Figure 6D**). On the basis of these observations, we hypothesize that CIC is closely linked to immune-related pathways, inflammatory responses and metabolic processes.

### 3.6 Analyzing the immune infiltration landscape and immunotherapeutic efficacy

**Figure 7:**
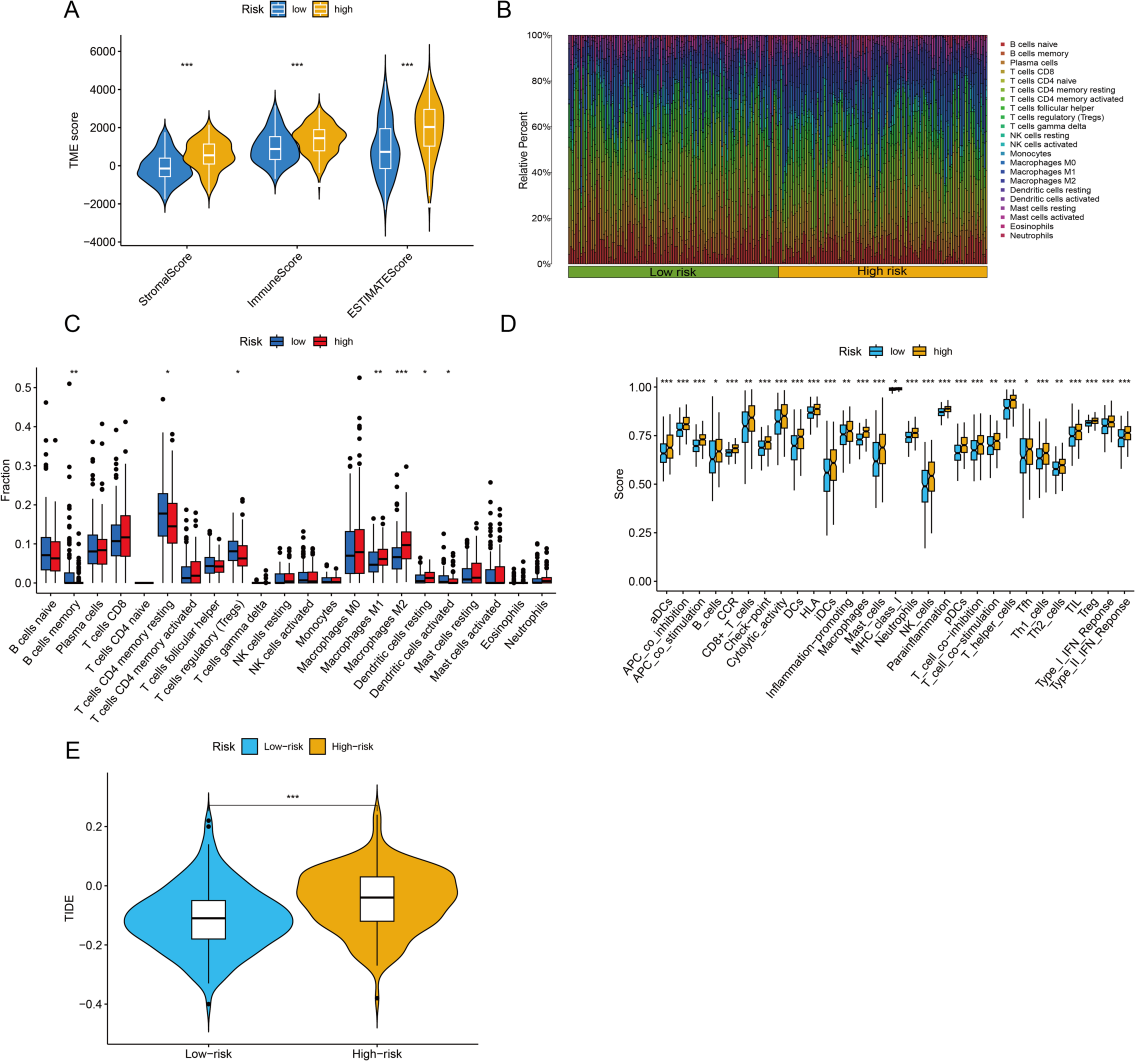
Immune infiltration analysis and assessment of immunotherapy outcomes. **(A)** The ESTIMATE algorithm was used to assess differences in immune, stromal and ESTIMATE scores between the two groups. **(B, C)** The CIBERSORT algorithm was used to evaluate differences in the abundances of 22 types of immune cells between the two groups. **(D)** The single-sample Gene Set Enrichment Analysis (ssGSEA) algorithm was used to analyze immune cell infiltration and immune functions. **(E)** Tumor Immune Dysfunction and Exclusion (TIDE) scores were calculated to predict immune evasion and response to immune checkpoint inhibitors. *P <0.05, **P <0.01, ***P <0.001.

The TME is central to the progression and treatment of GC. Therefore, we analyzed the TME in different risk categories using different algorithms. The ESTIMATE algorithm indicated that both the immune score and the ESTIMATE score were notably lower in the low-risk group than in the high-risk group (*P <*0.001 for all) (**Figure 7A**). To assess the proportions of 22 types of tumor-infiltrating immune cells, the CIBERSORT algorithm was utilized. The analysis results revealed disparate distributions within the two distinct risk groups (**Figure 7B**). As shown in **Figure 7C**, the box plot revealed that the low-risk group presented reduced numbers of M1 macrophages, M2 macrophages and resting dendritic cells, whereas the numbers of memory B cells, CD4 resting memory T cells, Tregs and activated dendritic cells were higher (*P <*0.05). The ssGSEA algorithm was also used to examine immune cell infiltration and immune function in different risk groups. The results revealed that the low-risk group had lower frequencies of various immune cells and weaker immune responses (*P <*0.05 for all) (**Figure 7D**). Finally, the TIDE algorithm was used to investigate the correlation between the risk score and response to immunotherapy. The results revealed that the high-risk group displayed a suboptimal response to ICI therapy. Although clinical ICI response data (e.g., objective response rates) are lacking here, TIDE has been extensively validated in GC cohorts for predicting anti-PD-1/PD-L1 efficacy (29, 30). Our risk score alignment with TIDE (**Figure 7E**) thus provides a bioinformatic proxy for immunotherapy resistance, which is consistent with the immunosuppressive TME features observed in high-risk patients (e.g., elevated Tregs/M2 macrophages).

This paradox essentially reflects a ‘quality-quantity imbalance’ in the TME: although the total immune cell count increases in the high-risk group (ESTIMATE score), the dominance of suppressive subsets (e.g., Tregs and M2 macrophages; **Figure 7C**) leads to ‘functional immune silencing.’ This finding aligns with the ‘immunosuppressive network’ theory proposed by Zheng et al. (34), where Tregs directly suppress effector T cells via IL-10 and TGF-β secretion, and M2 macrophages remodel the immunosuppressive stroma through ARG1 and MMP9 expression. Additionally, the elevated TIDE score in the high-risk group suggests T cell exhaustion, potentially associated with high PD-L1 expression in suppressive cells or tumor cell-secreted chemokines (e.g., CCL2) recruiting suppressive cells (19).

### 3.7 Analysis of the landscape of somatic mutations

**Figure 8:**
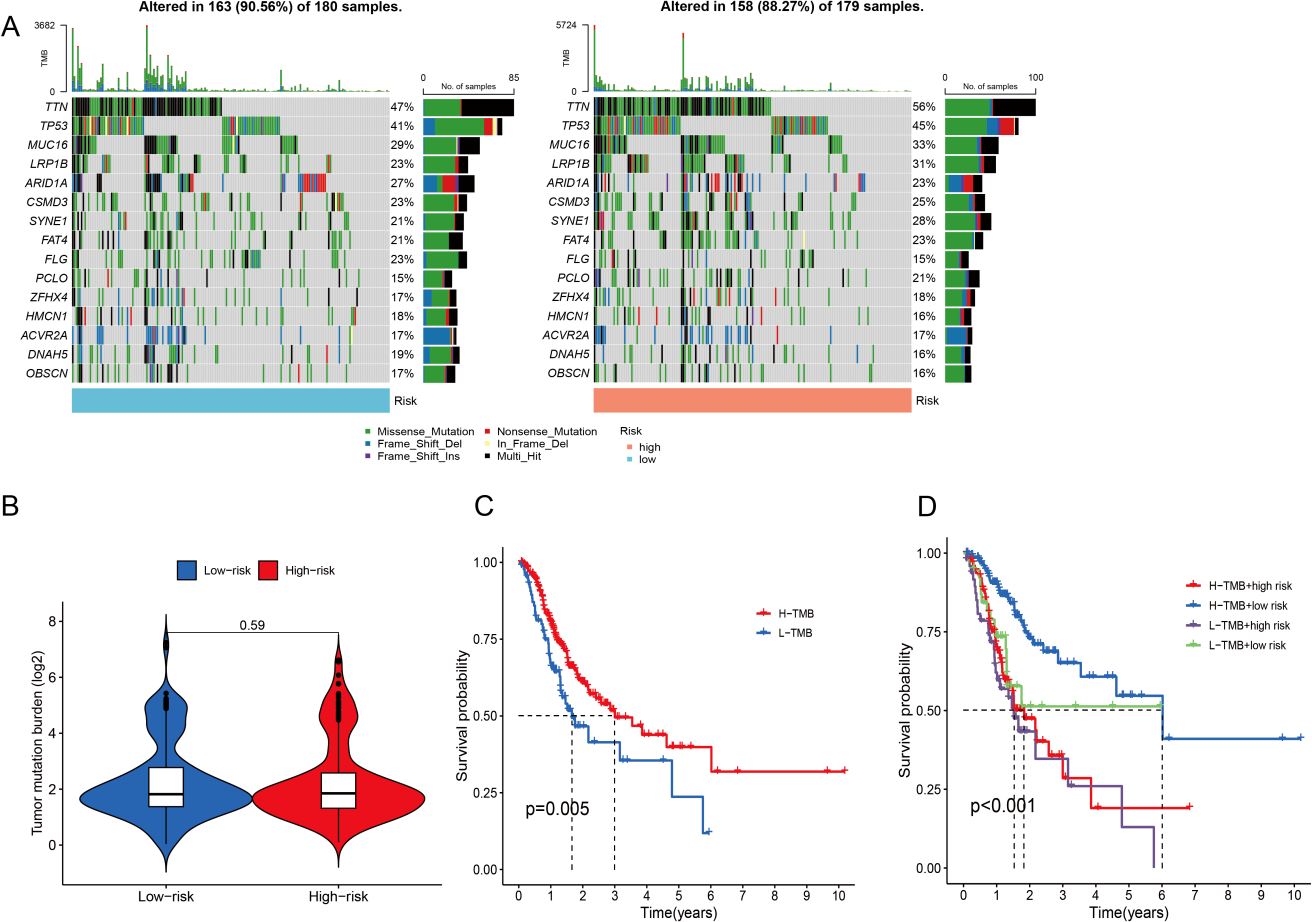
Analysis of the somatic mutation landscape. **(A)** Mutation distribution in the low-risk group and the high-risk group. **(B)** Differences in TMB scores between different risk groups. **(C, D)** K–M analysis of OS in different TMB groups.

When the somatic mutation rates between the two risk categories were compared, the low-risk group presented a higher mutation rate, with 163 out of 180 samples (90.56%) showing mutations, than did the high-risk group, where 158 out of 179 samples (88.27%) presented mutations. The top 15 genes responsible for these mutations are shown in **Figure 8A**. In addition, our analysis revealed no significant difference in TMB scores between the two risk categories (*P* = 0.59) (**Figure 8B**). When GC patients were stratified into low- and high-TMB groups on the basis of the median TMB score, K–M analysis revealed that individuals in the low-TMB subgroup had significantly better OS than those in the high-TMB subgroup did (*P* = 0.005) (**Figure 8C**). The efficiency of the TMB and risk score in predicting the prognosis of GC patients was evaluated by integrating these two factors. K–M analysis revealed that patients with both low TMB and low risk scores had the best OS, whereas patients with high TMB and high risk scores had the worst OS (*P <*0.001) (**Figure 8D**).

### 3.8 Analyzing drug reactions

**Figure 9:**
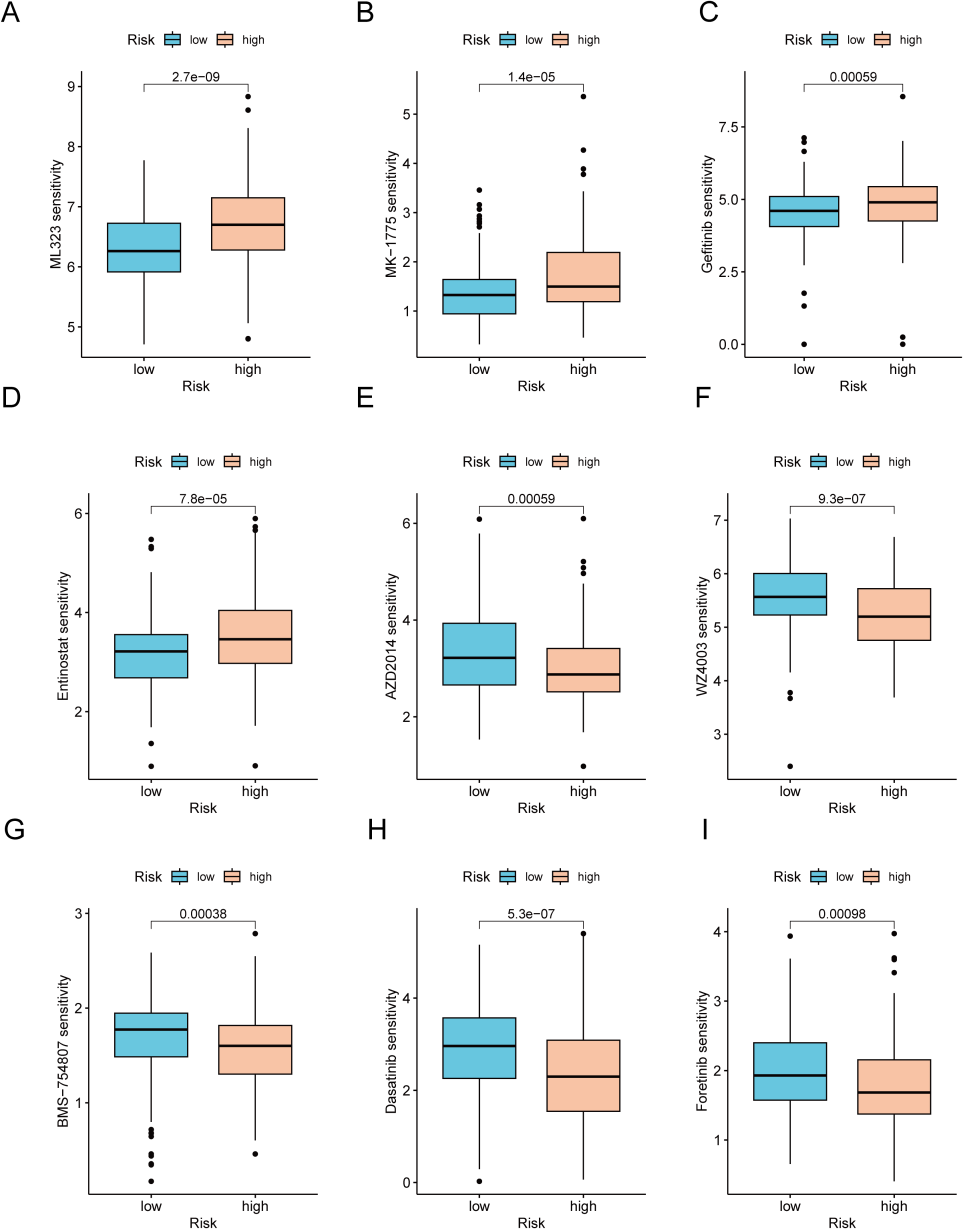
Analysis of therapeutic sensitivity. **(A)** ML323. **(B)** MK-1775. **(C)** Gefitinib. **(D)** Entinostat. **(E)** AZD2014. **(F)** WZ4003. **(G)** BMS-754807. **(H)** Dasatinib. **(I)** Foretinib.

Considering the notable disparities in prognosis and the immune microenvironment between the two risk categories, we conducted a search for drugs that are responsive to precision therapy for patients in each group. The R package oncoPredict facilitates the association of antitumor drugs with biomarkers, enabling the prediction of patient responses to various anticancer medications (35). Using *oncoPredict*, we identified potential antitumor drugs for patients with GC across different risk categories. The IC50 values of four drugs (ML323, MK-1775, gefitinib and entinostat) were lower in the low-risk group. This indicates that these drugs provide more benefit to low-risk GC patients (**Figures 9A–D**). Conversely, the IC50 values of the other five drugs (AZD2014, WZ4003, BMS-754807, dasatinib and foretinib) were higher in the low-risk group. These findings suggest that these drugs are more effective in patients with high-risk GC (**Figures 9E–I**).

### 3.9 Expression patterns of CICRlncRNAs

**Figure 10:**
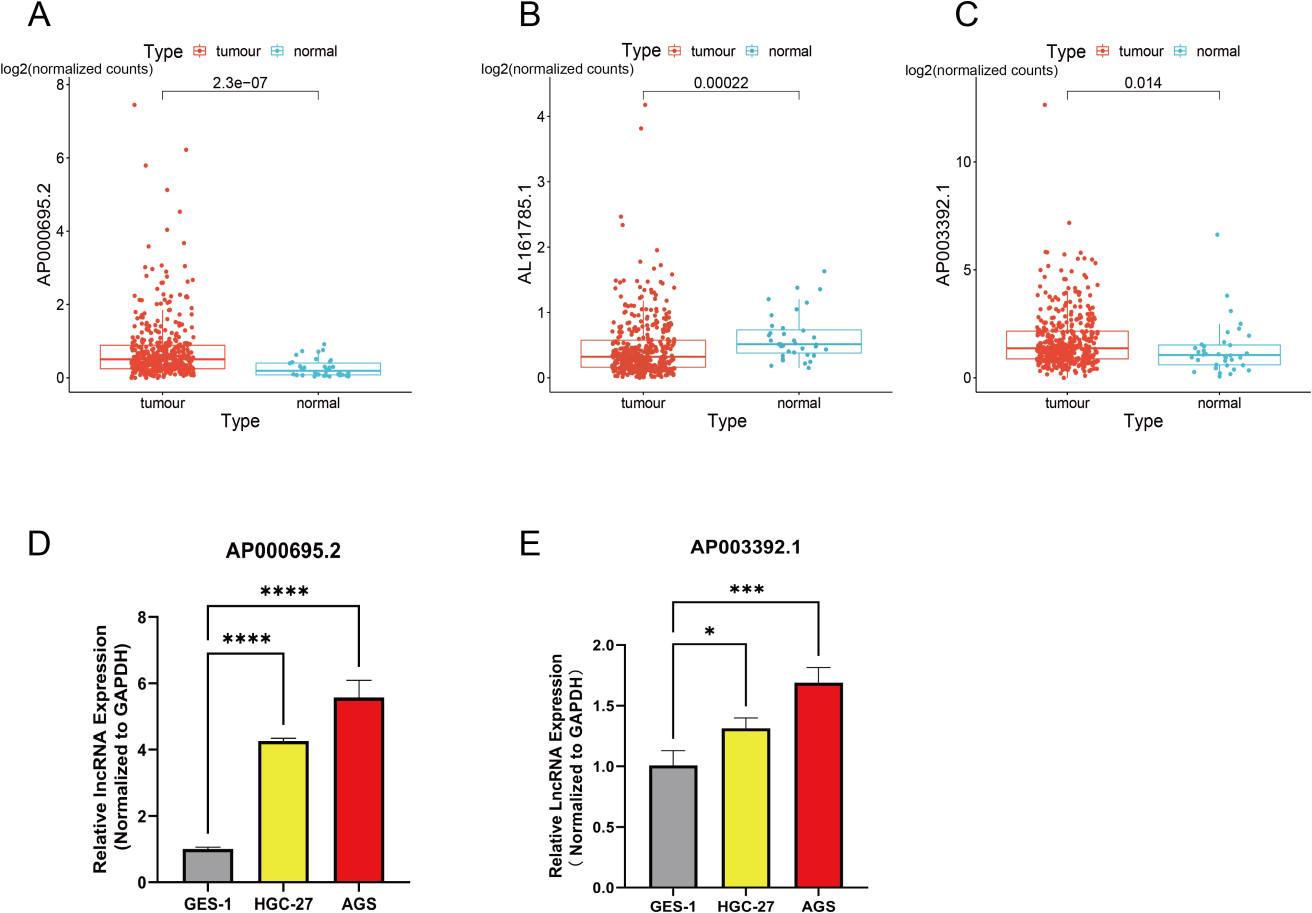
Exploration of the expression of CICRlncRNAs in GC. Expression pattern of **(A)** AP000695.2. **(B)** AL161785.1. **(C)** AP003392.1. qRT‒PCR was used to detect the expression of **(D)** AP000695.2. **(E)** AP003392.1 expression in normal human gastric mucosal epithelial cells (GES-1) and GC cells (HGC-27, AGS). *P <0.05, ***P <0.001, ****P <0.0001.

The expression levels of three CICRlncRNAs were initially assessed in GC and normal samples via the TCGA database. As depicted in **Figure 10B**, AL161785.1 exhibited decreased expression, whereas AP000695.2 and AP003392.1 presented increased expression in GC samples relative to normal samples, as illustrated in **Figures 10A, C**. To assess the reliability of the CICRlncRNA-based risk model, qRT–PCR was conducted to quantify the expression levels of AP000695.2 and AP003392.1 in both human gastric mucosal epithelial cells (GES-1) and GC cells (AGS and HGC-27). As depicted in **Figures 10D, E**, these two genes were markedly upregulated in GC cells compared with normal gastric cells. Taken together, these results suggest that the CICRlncRNA-based risk model has a certain degree of reliability.

### 3.10 Verification of the impact of AP000695.2 gene silencing on the malignant phenotype of GC cells

**Figure 11:**
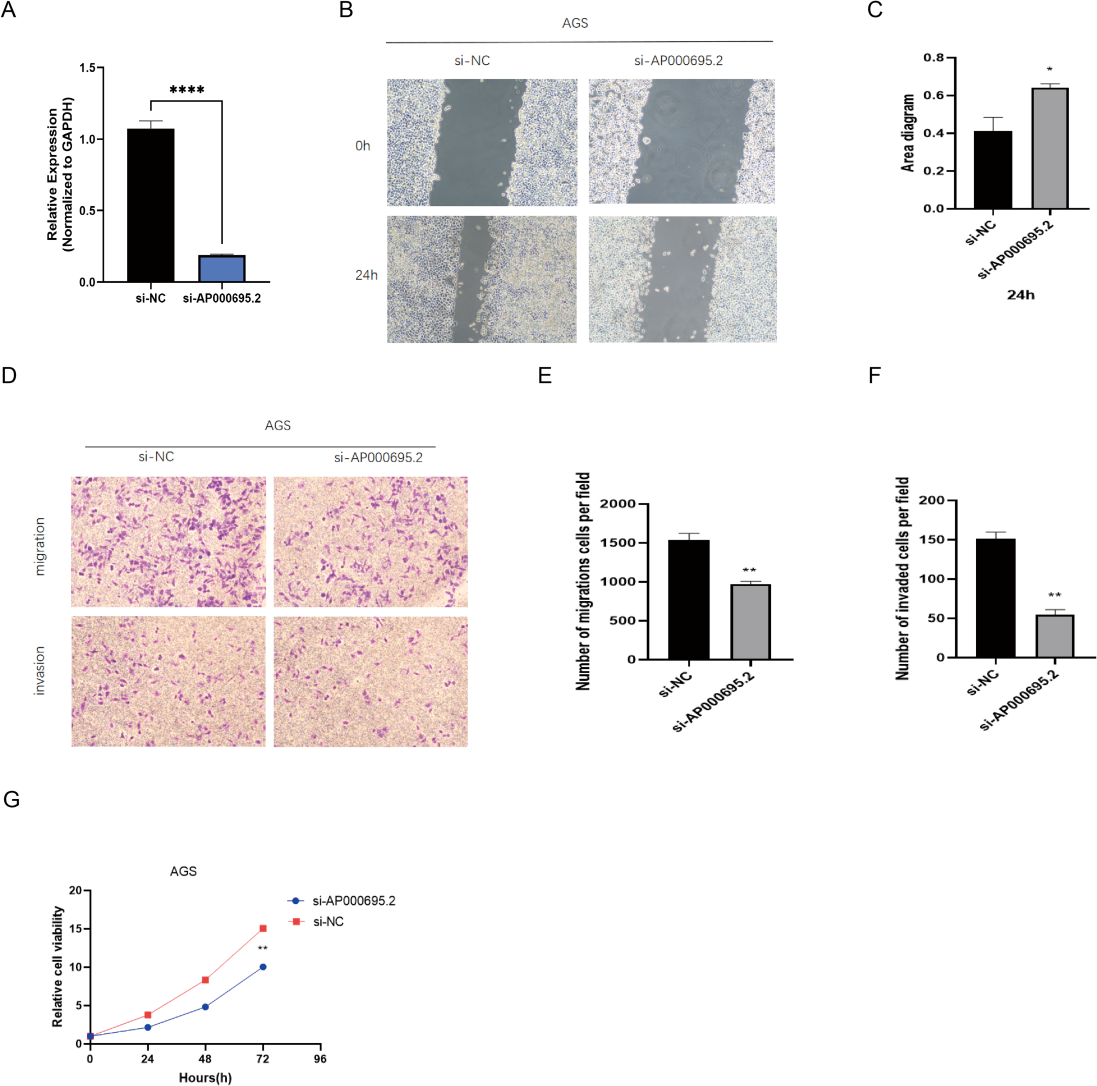
Exploration of the expression of CICRlncRNAs in GC. **(A)** qRT‒PCR analysis of the relative expression of AP000695.2 in the transfected cells. **(B, C)** A scratch assay for assessing migration ability of the cells. **(D–F)**. Transwell assays for assessing the invasion ability of the cells. **(G)** A CCK-8 assay was performed to evaluate the proliferative capacity of transfected GC cells. *P <0.05, **P <0.01, ****P <0.0001.

Based on the prominent AP000695.2 expression in AGS cells within the GC cell model (**Figure 10A**), we selected this cell line for functional studies. Given its significant clinical relevance in the prognostic model and differential expression, we used shRNA interference to establish a stable AP000695.2-knockdown cell model and evaluate its biological functions. QRT–PCR analysis (**Figure 11A**) confirmed that AP000695.2 expression in the siRNA group was reduced by >80% compared to the negative control (*P <*0.0001). In the cell motility assays, the scratch - wound healing assays revealed a significant reduction in migration speed in the interference group (*P <*0.05, **Figures 11B, C**). Transwell assays showed a 42% decrease in migrated cells indicating impaired migration capacity independent of proliferation in the treated group (*P <*0.01, **Figures 11D, E**), and Matrigel invasion assays indicated a roughly 35% drop in invasive ability post - silencing (*P <*0.01, **Figure 11F**). Moreover, CCK-8 assays demonstrated that AP000695.2 inhibition significantly reduced metabolic activity, with a 28% lower absorbance in the treated group at 72 hours (**Figure 11G**). The statistical evidence from these experiments aligns with prior bioinformatics predictions, confirming that AP000695.2 plays a key role in promoting tumor cell migration, invasion, and proliferation during GC progression.

## 4 Discussion

GC is a common malignancy worldwide. The absence of early symptoms and the rarity of screening often result in the majority of patients being diagnosed at advanced stages (36). Traditional diagnostic approaches, such as gastroscopy and histopathological analysis, are not effective in the early detection of GC. The clinical utility of common protein biomarkers, such as carcinoembryonic antigen (CEA), carbohydrate antigen 19-9 (CA19-9), and carbohydrate antigen 72-4 (CA72-4), is limited due to their insufficient sensitivity and specificity for GC screening (37). Although immunotherapy is an option for the treatment of GC, approximately two-thirds of patients with advanced disease do not respond well to ICI therapy. This resistance may be due to certain factors in the TME that allow tumor cells to evade immunotherapy (21, 38). In conclusion, to improve the diagnosis of early-stage GC and minimize the risk of metastasis, we used bioinformatics tools to identify biomarkers associated with GC prognosis. These biomarkers could aid in the development of more effective immunotherapy strategies.

Entosis is a cellular process in which one cell is engulfed by another, resulting in the formation of a ‘cell-in-cell’ (CIC) structure. This phenomenon has been implicated in the development and progression of cancer (39). The term “cell-in-cell” describes the internalization of a viable cell by another, with the engulfed cell being encapsulated within large vesicles of the host cell (40, 41). Although this structure was first observed over a century ago, its biological significance has often been neglected (42). In several types of cancer, the presence of intracellular structures is associated with a poor prognosis (8). Research on the role of intracellular cells in cancer is still limited. However, one study suggested that these structures may serve as potential prognostic biomarkers, with their predictive value depending on the breast cancer subtype and the underlying biological mechanisms of intracellular cell formation (43). Elevated levels of homotypic CICs, a specific category of intracellular structures, have been shown to be significantly correlated with reduced overall and disease-free survival in patients with non-small cell lung cancer. The aggressive nature of tumor cells may facilitate the formation of CICs, thereby promoting tumor invasion, progression and metastasis (44). Leonardo et al. pioneered the use of sphere bioprinting to investigate intracellular cell events in oral cancer and reported that intracellular cells are more frequently detected in the proliferative regions of spheres where cancer cells are cocultured with cancer-associated fibroblasts (CAFs) (45). Collectively, these studies offer significant perspectives on the identification of prognostic biomarkers and the exploration of potential therapeutic targets.

Extensive studies have shown that lncRNAs can either promote or inhibit tumor formation by modulating gene signaling pathways, making them potential biomarkers (46–48). Researchers have developed a model using seven non-coding RNA molecules to predict the prognosis of breast cancer patients, suggesting that these molecules may play specific roles in breast cancer development (49). However, the function of CICRlncRNAs in GC is still unclear. In this study, we identified CICRlncRNAs based on genes related to cellular nesting and investigated their effects on tumor progression and prognosis in patients with GC.

A sequential approach to dimensionality reduction was employed, utilizing LASSO regression followed by Cox regression analysis. This process aimed to minimize the number of feature parameters, thereby facilitating the construction of a prognostic model. This technique has been documented in several studies (50, 51). Three CICRlncRNAs, AP003392.1, AP000695.2 and AL161785.1, were significantly associated with GC prognosis and were used to develop a prognostic model. A nomogram was used to improve the accuracy of survival prediction for GC patients at 1, 3 and 5 years. All three CICRlncRNAs were correlated with GC. Min Jiang and colleagues constructed a prognostic risk model for GC based on long non-coding RNAs associated with pyroptosis (PRlncRNAs). Six CICRlncRNAs, including AP003392.1, were selected using techniques such as univariate and multivariate Cox regression analyses. The study further revealed that AP003392.1 is significantly differentially expressed in GC cell lines compared to normal cell lines, indicating its potential as an important prognostic biomarker for GC (31). In a recent investigation, scientists developed a molecular signature model that included four lncRNAs associated with ferroptosis prognosis, such as AP003392.1, to assess their predictive power for GC outcomes. The results showed that this model is a reliable predictor of GC prognosis. Furthermore, four lncRNAs, including AP003392.1, were confirmed in GC cell lines (16). Yun Cheng and colleagues investigated the expression profile, diagnostic significance and prognostic implications of the lncRNA AP000695.2 in GC. Their results revealed that AP000695.2 is abnormally upregulated in 19 different types of cancer, including GC, and is associated with reduced patient survival. Multivariate Cox regression analysis validated AP000695.2 as an independent predictor of OS and PFS. In addition, ROC analysis highlighted its potential as a diagnostic tool (52). Additionally, AP000695.2 is positively associated with various tumor-infiltrating immune cells. A recent study developed a robust prognostic model using eight costimulatory molecules, which may broaden the spectrum of cancer treatment strategies. Importantly, the lncRNA AP000695.2 is overexpressed in GC cell lines. *In vitro* experiments revealed that it promotes the proliferation, invasion, and migration of GC cells, indicating its potential as a therapeutic target for GC (53). In a particular study, six lncRNAs, including AL161785.1, were identified through univariate Cox regression and multivariate Cox analysis. A predictive model was subsequently developed. The model classified patients with GC into high-risk and low-risk groups. Subsequent survival analysis revealed a statistically significant disparity in survival outcomes between the two groups (*P <*0.001). Moreover, the ROC analysis revealed an AUC of 0.686. On the basis of these findings, researchers drew the conclusion that AL161785.1 is among the lncRNAs related to the prognosis of GC and has potential predictive value for the prognosis of GC patients (54). Combining these findings with our research results, these three lncRNAs may be involved in different pathogenesis patterns of GC, and further investigation of their specific roles in GC is warranted.

To explore the biological roles and signaling pathways associated with the three CICRlncRNAs, enrichment analysis was performed. The results showed that these lncRNAs are associated mainly with immune and metabolic pathways. According to the GSEA results, CICRlncRNAs have the potential to impact pathways related to immunity and apoptosis. Additionally, they are linked to the spliceosome and nitrogen metabolism pathways. While our bioinformatic analyses and previous studies provide a foundation for these hypotheses, direct experimental evidence is required to confirm these mechanisms. For example, previous studies have demonstrated that lncRNAs can function as ceRNAs by competitively binding to microRNAs, thereby relieving the repression of immunosuppressive factors. Our analysis shows that AP000695.2 is highly expressed in gastric cancer and positively correlated with multiple tumor-infiltrating immune cells, which provides a basis for our speculation (19). Furthermore, research has highlighted the role of CIC structures in secreting cytokines such as TGF-β and IL-10, which recruit Tregs and polarize macrophages to the M2 phenotype, establishing an immune-excluded TME (7). This evidence supports our hypothesis regarding the potential role of AP000695.2 in enhancing cytokine production. However, we fully acknowledge that these mechanisms require direct experimental validation.

A thorough analysis of the characteristics of the TME in GC is essential to help understand how the tumor responds to immunotherapy. Moreover, it provides innovative approaches for cancer therapy (55). This study investigated the correlation between the risk score and changes in the TME in GC patients. The stromal score, immune score and ESTIMATE score were significantly elevated in the high-risk cohort. The heightened immune cell infiltration and augmented immune function in the high-risk group offer a partial rationale for the disparity in OS between the two risk categories. Existing studies have demonstrated that Tregs play a pivotal role in dampening antitumor immune responses. They achieve this by either inhibiting the functions of effector cells or secreting immunosuppressive cytokines, which are essential for preserving immune tolerance and mitigating immune reactions (34). Research has revealed substantial disparities in Treg cell counts across various clinical stages of GC. Moreover, Treg infiltration is linked to an unfavorable prognosis in the majority of solid malignant tumors. For instance, in colorectal cancer, Treg infiltration serves as an independent prognostic indicator, with a higher infiltration rate correlating to a reduced OS for patients. Additionally, a meta-analysis ascertained the prognostic and clinicopathological significance of tumor-associated macrophages (TAMs) in GC patients. The findings revealed that a high density of M1-type TAMs is associated with increased OS, whereas a high density of M2-type TAMs is indicative of a poorer prognosis (56). Several references also mention the same view (57–59).

Prior research has demonstrated that M1-type TAMs are capable of releasing IL-24, CCL2, and TNF-α. The secretion of these cytokines serves to diminish cellular activity and increases the susceptibility of cells to chemotherapy agents. Consequently, this mechanism effectively impedes the advancement of GCs (60–62). M2-type TAMs exert a significant influence the progression of GC. Specifically, they secrete exosomes (63), miR-588 (64), lncRNAs (65), and the proteins MMP2 (66) and MMP9 (67). While high-risk patients exhibited elevated immune infiltration, the dominance of immunosuppressive subsets (Tregs and M2 macrophages) creates a ‘deceptive hot’ microenvironment that actively suppresses antitumor immunity. This finding aligns with emerging evidence that not all immune-rich tumors are immunotherapy responsive (68). Specifically, CIC structures may secrete cytokines (e.g., TGF-β and IL-10) that recruit Tregs and polarize macrophages toward the M2 phenotype (19), thereby establishing an immune-excluded TME despite high cellularity. Our data corroborate this finding: high-risk patients showed increased TIDE dysfunction scores (**Figure 7E**), indicating T-cell exhaustion, and elevated Treg/M2 ratios (**Figure 7C**), which are established biomarkers of ICI resistance (69). Thus, the paradoxical coexistence of abundant infiltration and poor ICI response reflects qualitative defects in immune activation rather than quantitative deficiencies. In addition to these secretions, M2 TAMs release various chemotactic factors (70) and engage in metabolic reprogramming (71, 72). These actions collectively promote cell proliferation and metastasis, confer chemoresistance, and ultimately intensify the progression of GC (73). M2 macrophages play a predominant role in the TME (74). Our research revealed that within the TME of patients with GC, the proportion of Tregs and M2 macrophages was markedly elevated in the high-risk group. This immunosuppressive shift aligns with the known role of CIC structures in secreting cytokines (e.g., TGF-β and IL-10) that recruit Tregs and polarize macrophages toward an M2 phenotype (7, 19). Based on the strong co-expression of CICRlncRNAs with CIC-related genes (**Figure 2E**) and established lncRNA functions in GC (15, 74), we hypothesize that AP000695.2 could potentially contribute to immunosuppression via (i) acting as a competitive endogenous RNA (ceRNA) to derepress immunosuppressive factors and/or (ii) enhancing cytokine production within CIC formations. However, these mechanisms remain speculative and warrant experimental validation. Critically, our data demonstrate that the CICRlncRNA signature correlates with functional outcomes (T cell dysfunction, M2 polarization; **Figure 7**) consistent with CIC-mediated immune evasion (7, 67), providing a rationale to investigate direct CIC regulation in future work. The experimental validation of the role of AP000695.2 in promoting GC cell invasion (**Figure 11**) supports its functional impact on TME remodeling. Nevertheless, the precise molecular pathways require further investigation. Furthermore, in the present study, the abundance of the majority of immune cells and the immune function scores were greater in the high-risk group than in the low-risk group. These findings suggest that individuals with high-risk scores exhibit heightened immune activity.

Regarding tumor mutational burden (TMB), the non-significant difference between risk groups warrants nuanced interpretation beyond sample size considerations. Gastric cancer exhibits profound mutational signature heterogeneity—including microsatellite instability (MSI), Epstein-Barr virus (EBV) infection, and chromosomal instability (CIN) subtypes—that may confound TMB-risk associations (75). Crucially, these molecular subtypes demonstrate divergent TMB profiles and clinical behaviors: MSI-high tumors typically show elevated TMB and favorable immunotherapy response, whereas CIN tumors often exhibit intermediate TMB but poorer outcomes (76). The balanced representation of these subtypes across our risk groups (inferred from comparable prognosis stratification) likely contributes to the observed TMB homogeneity. This further underscores that our CICRlncRNA-based model captures biological features distinct from conventional genomic classifiers.

Additionally, the TIDE algorithm was employed to assess the correlation between the risk score and the response to ICIs. The findings revealed that the high-risk group displayed a suboptimal response to ICI therapy. This implies that CICRlncRNAs could potentially serve as promising biomarkers for forecasting the response of GC patients to ICIs.

Several researches have explored the correlation between the TMB and immune infiltration in the progression and prognosis of GC. These findings suggest that a high TME is likely linked with an unfavorable prognosis for patients with GC (77). Within the scope of this research, a significant positive correlation was identified between the risk score and TMB. Specifically, patients in the high-risk score and high-TMB groups had the shortest survival and the most unfavorable prognosis. Conversely, patients in the low-risk score and low-TMB score groups had the longest survival and the most favorable prognosis. Such a finding underscores the precise predictive capability of the risk score regarding the outcomes of immunotherapy in individuals suffering from GC. In addition, the sensitivity of patients with GC to various anticancer drugs was assessed in two different risk categories. In the low-risk cohort, USP1-UAF1 inhibitors (ML-323), adavosertib, gefitinib and entinostat showed superior therapeutic efficacy. Conversely, in the high-risk group, vistusertib (AZD2014), NUAK kinase inhibitors (WZ4003), IGF-1R/IR inhibitors (BMS-754807), dasatinib and foretinib showed improved efficacy. These results could serve as a valuable reference to guide the clinical management of patients with GC. For clinical translation, CICRlncRNAs could be detected in liquid biopsies (e.g., plasma exosomal RNA via RT–qPCR) or tumor tissues (via RNA *in situ* hybridization), similar to established lncRNA biomarkers such as H19 in GC (78). Integration with routine histopathology or blood tests would enable risk stratification during diagnosis.

This study has several limitations. First, the reliance on retrospective data from the TCGA database may introduce selection bias, and the limited number of normal tissue samples (n = 36) could affect model generalizability. Second, experimental validation of CICRlncRNAs was incomplete (e.g., AL161785.1 remained unverified owing to primer limitations), leaving gaps in confirming their biological roles. Third, we emphasize that resolving AL161785.1’s function through advanced methodologies (e.g., long-read sequencing, RNA-FISH) is a key objective of our ongoing research program. Third, while bioinformatic analyses revealed associations between CICRlncRNAs and immunosuppressive TME features (e.g., Tregs/M2 macrophages), direct mechanistic links remain unvalidated. Specifically, how AP000695.2 regulates cytokine secretion or ceRNA networks in CIC contexts needs experimental interrogation. Additionally, although algorithms such as CIBERSORT exhibit good consistency with single-cell sequencing data (29, 79), their resolution remains limited by bulk RNA-seq data and cannot capture spatial immune suppression networks at the single-cell level (e.g., molecular interactions at tumor-immune cell synapses). Future studies could combine single-cell RNA sequencing and spatial transcriptomics to further elucidate the specific mechanisms of CICRlncRNA in the recruitment and polarization of Tregs/M2 macrophages (79). Fourth, clinical translation requires further validation through prospective, multicenter cohorts to assess real-world applicability. Fifth, the drug sensitivity predictions (e.g., gefitinib/dasatinib) derived from *oncoPredict* lack experimental validation in cell lines or patient-derived models. While GDSC2-based computational screening is widely used for hypothesis generation, its clinical applicability requires functional confirmation through *in vitro* assays (e.g., CCK-8/PI staining) and preclinical models. Additionally, genetic heterogeneity and treatment history were not fully addressed (35). While CIBERSORT provided valuable insights into immune infiltration patterns, we acknowledge its technical limitations: Resolution constraints: The algorithm cannot reliably quantify rare immune subsets (e.g., γδ T cells, pDCs) constituting <2% of the microenvironment, as its deconvolution accuracy drops substantially below this threshold (79). Matrix obsolescence: The LM22 signature matrix omits newly discovered immune phenotypes (e.g., TRM, ICOS+ Tregs) identified post-2015 (80). Critically, our core findings—elevated Tregs/M2 macrophages in high-risk patients and immunosuppressive TME features—were consistently replicated across three orthogonal methods (ESTIMATE, ssGSEA, and TIDE; **Figure 7**). This multi-algorithm convergence mitigates CIBERSORT-specific biases and reinforces conclusion validity (29). Sixth, although siRNA-mediated knockdown of AP000695.2 achieved high efficiency (>80%, **Figure 11A**) and consistently attenuated malignant phenotypes (proliferation, migration, invasion), the absence of rescue experiments (e.g., re-expression of AP000695.2) prevents definitive causal inference regarding on-target effects. While high-efficiency siRNA knockdown substantially reduces off-target artifacts (81) and phenotypic concordance with bioinformatic predictions supports biological relevance, future studies using CRISPR-based genetic rescue models are warranted for causal validation. Finally, the prognostic model was validated internally but not in external cohorts due to limited availability of independent gastric cancer datasets with comprehensive CIC-related annotations.

Future research should prioritize expanding sample diversity, integrating multiomics data (e.g., proteomics, epigenetics), and conducting mechanistic studies (e.g., CRISPR-based functional assays) to uncover causal relationships. Prospective clinical trials are needed to validate the prognostic model and explore its utility in guiding immunotherapy strategies. Additionally, developing standardized assays for CICRlncRNA detection could enhance their clinical adoption as biomarkers. Addressing these gaps will advance personalized prognosis prediction and therapeutic targeting in patients with GC.

## 5 Conclusion

In summary, this study is the first to report the prognostic role of CICRlncRNAs in GC. A risk model integrating three CICRlncRNAs (AP003392.1, AP000695.2, AL161785.1) was constructed using TCGA-GC data, effectively stratifying patients into distinct risk groups with divergent survival outcomes. These findings provide novel insights into immune-related mechanisms and highlight the potential of CICRlncRNAs as biomarkers for prognosis and tailored therapeutic strategies in patients with GC. Future research should prioritize expanding sample diversity, integrating multiomics data (e.g., proteomics, epigenetics), and conducting mechanistic studies (e.g., CRISPR-based functional assays) to uncover causal relationships. Direct experimental validation is needed to determine the specific roles of CICRlncRNAs in CIC formation and immune evasion.

## Data Availability

The original contributions presented in the study are included in the article/**Supplementary Material**. Further inquiries can be directed to the corresponding author.
