# Supplementary material for: Cell-in-cell associated lncRNA signature predicts prognosis and immunotherapy response in gastric cancer
Source: Front Oncol. 2025 Jul 8;15:1597187. doi: 10.3389/fonc.2025.1597187 (PMC12280372; doi:10.3389/fonc.2025.1597187)
Supplement: Supplementary file 1 [file Table1.docx]

**Table S1.** Correlation of cell-in-cell related genes and cell-in-cell-related lncRNA in TCGA database.

| **Cell-in-cell** | **LncRNA** | **Cor** | ***P*** | **Regulation** |
| --- | --- | --- | --- | --- |
| MLANA | AC068790.4 | 0.405609614 | 9.53E-18 | positive |
| APP | AL356364.1 | 0.418707517 | 6.42E-19 | positive |
| MLANA | AC005776.2 | 0.423783996 | 2.19E-19 | positive |
| CYBB | MIAT | 0.508287779 | 1.89E-28 | positive |
| GZMB | MIAT | 0.431060991 | 4.51E-20 | positive |
| MLANA | AC009120.3 | 0.517001306 | 1.55E-29 | positive |
| RASA2 | AC009120.3 | 0.518849652 | 9.03E-30 | positive |
| AR | AC010226.1 | 0.620131894 | 3.83E-45 | positive |
| CDC20 | AC010226.1 | -0.407584117 | 6.39E-18 | negative |
| KIF2C | AC010226.1 | -0.462374265 | 3.25E-23 | negative |
| MAD2L1 | AC010226.1 | -0.401192151 | 2.30E-17 | negative |
| MAPT | AC010226.1 | 0.521233132 | 4.48E-30 | positive |
| MYLK | AC010226.1 | 0.679950907 | 3.29E-57 | positive |
| PCDH7 | AC010226.1 | 0.52553541 | 1.25E-30 | positive |
| PRKAA2 | AC010226.1 | 0.471389735 | 3.51E-24 | positive |
| PRNP | AC010226.1 | 0.568183688 | 1.35E-36 | positive |
| RNF146 | AC010226.1 | 0.451540998 | 4.33E-22 | positive |
| ROCK1 | AC010226.1 | 0.404576418 | 1.17E-17 | positive |
| S100B | AC010226.1 | 0.446937716 | 1.26E-21 | positive |
| SRF | AC010226.1 | 0.481746422 | 2.51E-25 | positive |
| TUBA1A | AC010226.1 | 0.599496035 | 1.47E-41 | positive |
| VCL | AC010226.1 | 0.564608311 | 4.62E-36 | positive |
| VIM | AC010226.1 | 0.407160886 | 6.96E-18 | positive |
| CTNNB1 | AL683807.1 | 0.418867609 | 6.21E-19 | positive |
| TP63 | AL683807.1 | 0.406320783 | 8.25E-18 | positive |
| KRT7 | LINC01605 | 0.463281508 | 2.61E-23 | positive |
| PIKFYVE | COX10-AS1 | 0.405020474 | 1.07E-17 | positive |
| RASA2 | COX10-AS1 | 0.415741512 | 1.19E-18 | positive |
| MLANA | AC093690.1 | 0.414850737 | 1.44E-18 | positive |
| MLANA | U73169.1 | 0.456132184 | 1.46E-22 | positive |
| RASA2 | U73169.1 | 0.461930007 | 3.62E-23 | positive |
| MLANA | C1RL-AS1 | 0.481007374 | 3.03E-25 | positive |
| RASA2 | C1RL-AS1 | 0.427826598 | 9.14E-20 | positive |
| CUEDC2 | AC098869.2 | -0.42969783 | 6.08E-20 | negative |
| CYBB | AC124014.1 | 0.481362071 | 2.77E-25 | positive |
| MLANA | AC124014.1 | 0.405824519 | 9.12E-18 | positive |
| PIKFYVE | AC124014.1 | 0.406334569 | 8.23E-18 | positive |
| RASA2 | AC124014.1 | 0.495826279 | 5.98E-27 | positive |
| RASA2 | SEC62-AS1 | 0.412699405 | 2.25E-18 | positive |
| PIKFYVE | AC005104.1 | 0.444202602 | 2.37E-21 | positive |
| RASA2 | AC005104.1 | 0.51625411 | 1.92E-29 | positive |
| MTMR7 | AC002511.2 | 0.425424853 | 1.54E-19 | positive |
| MTUS2 | AC002511.2 | 0.445744758 | 1.66E-21 | positive |
| ROCK1 | AL139099.2 | 0.404800804 | 1.12E-17 | positive |
| CALB2 | AC002401.4 | 0.502723067 | 8.99E-28 | positive |
| CDH3 | AC002401.4 | 0.42379953 | 2.18E-19 | positive |
| KRT7 | AC002401.4 | 0.488460569 | 4.31E-26 | positive |
| AR | AC011472.4 | 0.703811477 | 7.24E-63 | positive |
| CAV1 | AC011472.4 | 0.470599389 | 4.28E-24 | positive |
| ITGB1 | AC011472.4 | 0.421625053 | 3.46E-19 | positive |
| MAPT | AC011472.4 | 0.687098983 | 7.57E-59 | positive |
| MYLK | AC011472.4 | 0.798031863 | 3.23E-92 | positive |
| PCDH7 | AC011472.4 | 0.528590172 | 4.96E-31 | positive |
| PRKAA2 | AC011472.4 | 0.622656665 | 1.34E-45 | positive |
| PRNP | AC011472.4 | 0.680796693 | 2.12E-57 | positive |
| RNF146 | AC011472.4 | 0.486341579 | 7.54E-26 | positive |
| ROCK1 | AC011472.4 | 0.442117365 | 3.82E-21 | positive |
| S100B | AC011472.4 | 0.418992411 | 6.05E-19 | positive |
| SRF | AC011472.4 | 0.571662982 | 4.03E-37 | positive |
| TUBA1A | AC011472.4 | 0.751814517 | 3.65E-76 | positive |
| VCL | AC011472.4 | 0.713335717 | 2.76E-65 | positive |
| RNF146 | STARD4-AS1 | 0.497491348 | 3.80E-27 | positive |
| CYBB | AC011899.3 | 0.504684457 | 5.20E-28 | positive |
| MLANA | CDC42-IT1 | 0.437286693 | 1.14E-20 | positive |
| AR | AC007193.2 | 0.42342168 | 2.36E-19 | positive |
| PRKAA2 | AC007193.2 | 0.424260365 | 1.97E-19 | positive |
| MYLK | AC027575.2 | 0.46243395 | 3.20E-23 | positive |
| ROCK1 | AC027575.2 | 0.41183173 | 2.68E-18 | positive |
| TUBA1A | AC027575.2 | 0.482641887 | 1.99E-25 | positive |
| MLANA | AC010260.1 | 0.423819037 | 2.17E-19 | positive |
| RASA2 | AL133410.1 | 0.405424557 | 9.89E-18 | positive |
| MLANA | AL157871.5 | 0.406245733 | 8.38E-18 | positive |
| MYL2 | AC011498.1 | 0.464145006 | 2.11E-23 | positive |
| MLANA | AC016747.3 | 0.472741496 | 2.50E-24 | positive |
| RASA2 | AC016747.3 | 0.429007908 | 7.07E-20 | positive |
| ROCK1 | AC016747.3 | 0.473298218 | 2.17E-24 | positive |
| MYL2 | AL133243.1 | 0.434507837 | 2.11E-20 | positive |
| CTSB | AC068587.4 | 0.417349254 | 8.54E-19 | positive |
| AR | AC010478.1 | 0.702866555 | 1.24E-62 | positive |
| CAV1 | AC010478.1 | 0.414246613 | 1.63E-18 | positive |
| CDH1 | AC010478.1 | -0.435067 | 1.86E-20 | negative |
| ITGB1 | AC010478.1 | 0.478150328 | 6.33E-25 | positive |
| KIF2C | AC010478.1 | -0.445381993 | 1.81E-21 | negative |
| MAD2L1 | AC010478.1 | -0.418740888 | 6.37E-19 | negative |
| MAPT | AC010478.1 | 0.564511314 | 4.78E-36 | positive |
| MYLK | AC010478.1 | 0.81253929 | 3.81E-98 | positive |
| PCDH7 | AC010478.1 | 0.615411825 | 2.67E-44 | positive |
| PRKAA2 | AC010478.1 | 0.624805167 | 5.43E-46 | positive |
| PRNP | AC010478.1 | 0.697503578 | 2.57E-61 | positive |
| RNF146 | AC010478.1 | 0.455663393 | 1.63E-22 | positive |
| SRF | AC010478.1 | 0.580034985 | 2.07E-38 | positive |
| STMN2 | AC010478.1 | 0.475183787 | 1.35E-24 | positive |
| TUBA1A | AC010478.1 | 0.796735015 | 1.04E-91 | positive |
| VCL | AC010478.1 | 0.707982042 | 6.49E-64 | positive |
| VIM | AC010478.1 | 0.41593542 | 1.15E-18 | positive |
| CTSB | AF131216.4 | 0.511754584 | 7.04E-29 | positive |
| CTSS | AL591468.1 | 0.478215667 | 6.22E-25 | positive |
| CYBB | AL591468.1 | 0.519848742 | 6.73E-30 | positive |
| GZMB | AL591468.1 | 0.57305458 | 2.48E-37 | positive |
| AR | RAP2C-AS1 | 0.724721222 | 2.60E-68 | positive |
| CAV1 | RAP2C-AS1 | 0.427893307 | 9.01E-20 | positive |
| ITGB1 | RAP2C-AS1 | 0.517753861 | 1.24E-29 | positive |
| KIF2C | RAP2C-AS1 | -0.434230089 | 2.24E-20 | negative |
| MAD2L1 | RAP2C-AS1 | -0.421454526 | 3.59E-19 | negative |
| MAPT | RAP2C-AS1 | 0.621899166 | 1.84E-45 | positive |
| MYLK | RAP2C-AS1 | 0.822186193 | 2.21E-102 | positive |
| PCDH7 | RAP2C-AS1 | 0.61272192 | 7.96E-44 | positive |
| PRKAA2 | RAP2C-AS1 | 0.64140507 | 3.98E-49 | positive |
| PRNP | RAP2C-AS1 | 0.691063991 | 8.92E-60 | positive |
| RNF146 | RAP2C-AS1 | 0.467623826 | 8.96E-24 | positive |
| ROCK1 | RAP2C-AS1 | 0.454715049 | 2.05E-22 | positive |
| SRF | RAP2C-AS1 | 0.633868161 | 1.11E-47 | positive |
| TUBA1A | RAP2C-AS1 | 0.732291285 | 2.08E-70 | positive |
| VCL | RAP2C-AS1 | 0.723415023 | 5.88E-68 | positive |
| VIM | Z97200.1 | 0.499984675 | 1.92E-27 | positive |
| AR | AL109741.1 | 0.440530964 | 5.47E-21 | positive |
| CDH1 | AL109741.1 | -0.41768347 | 7.96E-19 | negative |
| PRND | AL109741.1 | 0.471408293 | 3.49E-24 | positive |
| TGFB1 | AL109741.1 | 0.434971194 | 1.90E-20 | positive |
| TUBA1A | AL109741.1 | 0.40957953 | 4.26E-18 | positive |
| VIM | AL109741.1 | 0.443526382 | 2.77E-21 | positive |
| AR | AC025280.3 | 0.555134071 | 1.12E-34 | positive |
| IL6 | AC025280.3 | 0.410110803 | 3.82E-18 | positive |
| MYLK | AC025280.3 | 0.540291976 | 1.34E-32 | positive |
| PDPN | AC025280.3 | 0.475899768 | 1.12E-24 | positive |
| PRKAA2 | AC025280.3 | 0.431124626 | 4.45E-20 | positive |
| PRNP | AC025280.3 | 0.466995929 | 1.05E-23 | positive |
| SRF | AC025280.3 | 0.43301934 | 2.93E-20 | positive |
| TUBA1A | AC025280.3 | 0.528137352 | 5.69E-31 | positive |
| VCL | AC025280.3 | 0.556625148 | 6.81E-35 | positive |
| AR | AP001347.1 | 0.671763386 | 2.17E-55 | positive |
| MAPT | AP001347.1 | 0.618366814 | 7.95E-45 | positive |
| MYLK | AP001347.1 | 0.652001849 | 3.13E-51 | positive |
| PRKAA2 | AP001347.1 | 0.562291421 | 1.02E-35 | positive |
| PRNP | AP001347.1 | 0.601607656 | 6.48E-42 | positive |
| S100B | AP001347.1 | 0.513541943 | 4.22E-29 | positive |
| SRF | AP001347.1 | 0.531824367 | 1.86E-31 | positive |
| TUBA1A | AP001347.1 | 0.674567666 | 5.24E-56 | positive |
| VCL | AP001347.1 | 0.57105956 | 4.98E-37 | positive |
| AR | AP002884.1 | 0.48222974 | 2.21E-25 | positive |
| MAPT | AP002884.1 | 0.45948329 | 6.54E-23 | positive |
| MYLK | AP002884.1 | 0.634050375 | 1.03E-47 | positive |
| PCDH7 | AP002884.1 | 0.445289258 | 1.85E-21 | positive |
| PRKAA2 | AP002884.1 | 0.561854353 | 1.18E-35 | positive |
| PRNP | AP002884.1 | 0.514901005 | 2.85E-29 | positive |
| RNF146 | AP002884.1 | 0.415041829 | 1.38E-18 | positive |
| ROCK1 | AP002884.1 | 0.40141037 | 2.21E-17 | positive |
| SRF | AP002884.1 | 0.445393924 | 1.80E-21 | positive |
| TUBA1A | AP002884.1 | 0.546007033 | 2.18E-33 | positive |
| VCL | AP002884.1 | 0.539057373 | 1.98E-32 | positive |
| MLANA | ASH1L-IT1 | 0.505585799 | 4.04E-28 | positive |
| RAC1 | AC018648.1 | 0.432076434 | 3.61E-20 | positive |
| NTS | AL049836.1 | 0.488987728 | 3.75E-26 | positive |
| CALB2 | AL513123.1 | 0.434315262 | 2.20E-20 | positive |
| AR | CARMN | 0.710618395 | 1.38E-64 | positive |
| CAV1 | CARMN | 0.463686211 | 2.36E-23 | positive |
| ITGB1 | CARMN | 0.544624442 | 3.40E-33 | positive |
| MAPT | CARMN | 0.63131261 | 3.38E-47 | positive |
| MYLK | CARMN | 0.870962734 | 1.33E-128 | positive |
| PCDH7 | CARMN | 0.651777507 | 3.47E-51 | positive |
| PRKAA2 | CARMN | 0.692192857 | 4.82E-60 | positive |
| PRNP | CARMN | 0.706280009 | 1.75E-63 | positive |
| RNF146 | CARMN | 0.449972187 | 6.25E-22 | positive |
| ROCK1 | CARMN | 0.448611126 | 8.58E-22 | positive |
| SRF | CARMN | 0.658273661 | 1.62E-52 | positive |
| TUBA1A | CARMN | 0.720463374 | 3.66E-67 | positive |
| VCL | CARMN | 0.78447487 | 4.23E-87 | positive |
| CAV1 | GAS6-DT | 0.454268553 | 2.27E-22 | positive |
| PRNP | GAS6-DT | 0.441308355 | 4.59E-21 | positive |
| TUBA1A | GAS6-DT | 0.43020946 | 5.44E-20 | positive |
| MLANA | AC011933.4 | 0.413940741 | 1.74E-18 | positive |
| RASA2 | AC022973.4 | 0.465185909 | 1.63E-23 | positive |
| CDC20 | AC012073.1 | 0.430594821 | 5.00E-20 | positive |
| KIF2C | AC012073.1 | 0.52493081 | 1.49E-30 | positive |
| CUEDC2 | TRIM52-AS1 | 0.47272545 | 2.51E-24 | positive |
| MAP1LC3A | TRIM52-AS1 | 0.429025076 | 7.05E-20 | positive |
| TGFB1 | PDCD4-AS1 | 0.40098461 | 2.40E-17 | positive |
| CXCL8 | GK-AS1 | 0.423316157 | 2.42E-19 | positive |
| IL6 | GK-AS1 | 0.581358314 | 1.28E-38 | positive |
| PDPN | GK-AS1 | 0.595255657 | 7.43E-41 | positive |
| TF | AC012065.3 | 0.445555444 | 1.74E-21 | positive |
| MLANA | AC008537.4 | 0.415113791 | 1.36E-18 | positive |
| RASA2 | AC008537.4 | 0.428682658 | 7.59E-20 | positive |
| DIAPH1 | BOLA3-AS1 | -0.402240591 | 1.87E-17 | negative |
| PRKAA2 | BOLA3-AS1 | 0.444728141 | 2.10E-21 | positive |
| PRNP | BOLA3-AS1 | 0.412914049 | 2.15E-18 | positive |
| TM9SF4 | SNHG17 | 0.439723574 | 6.57E-21 | positive |
| AR | AC005180.2 | 0.614137509 | 4.49E-44 | positive |
| MAPT | AC005180.2 | 0.690098904 | 1.51E-59 | positive |
| MYLK | AC005180.2 | 0.74208732 | 3.13E-73 | positive |
| PCDH7 | AC005180.2 | 0.600748654 | 9.05E-42 | positive |
| PRKAA2 | AC005180.2 | 0.531296844 | 2.18E-31 | positive |
| PRNP | AC005180.2 | 0.593668349 | 1.35E-40 | positive |
| SRF | AC005180.2 | 0.591199771 | 3.43E-40 | positive |
| TUBA1A | AC005180.2 | 0.640813221 | 5.19E-49 | positive |
| VCL | AC005180.2 | 0.650830899 | 5.39E-51 | positive |
| MLANA | AL117381.1 | 0.409907525 | 3.98E-18 | positive |
| MLANA | AC118344.1 | 0.464110037 | 2.13E-23 | positive |
| ROCK1 | AC145422.1 | 0.432707549 | 3.14E-20 | positive |
| CYBB | LINC00892 | 0.496309852 | 5.24E-27 | positive |
| MAD2L1 | AC016205.1 | 0.438263777 | 9.13E-21 | positive |
| AR | AL356599.1 | 0.631464875 | 3.16E-47 | positive |
| MAPT | AL356599.1 | 0.577888499 | 4.46E-38 | positive |
| MYLK | AL356599.1 | 0.695863599 | 6.39E-61 | positive |
| PCDH7 | AL356599.1 | 0.482173208 | 2.24E-25 | positive |
| PRKAA2 | AL356599.1 | 0.565869473 | 3.00E-36 | positive |
| PRNP | AL356599.1 | 0.578477846 | 3.61E-38 | positive |
| RNF146 | AL356599.1 | 0.593491548 | 1.45E-40 | positive |
| ROCK1 | AL356599.1 | 0.446303483 | 1.46E-21 | positive |
| SRF | AL356599.1 | 0.455806106 | 1.58E-22 | positive |
| TUBA1A | AL356599.1 | 0.605093361 | 1.66E-42 | positive |
| VCL | AL356599.1 | 0.600387848 | 1.04E-41 | positive |
| PIKFYVE | NFYC-AS1 | 0.413316268 | 1.98E-18 | positive |
| RASA2 | NFYC-AS1 | 0.45909285 | 7.19E-23 | positive |
| RASA2 | AC092119.2 | 0.444206949 | 2.37E-21 | positive |
| MTMR7 | AL139383.1 | 0.520345715 | 5.82E-30 | positive |
| MTUS2 | AL139383.1 | 0.578378058 | 3.75E-38 | positive |
| CTSK | LINC00519 | 0.464079829 | 2.14E-23 | positive |
| TGFB1 | LINC00519 | 0.429513363 | 6.33E-20 | positive |
| AR | LINC02106 | 0.67569726 | 2.95E-56 | positive |
| CAV1 | LINC02106 | 0.427080298 | 1.07E-19 | positive |
| ITGB1 | LINC02106 | 0.429814703 | 5.93E-20 | positive |
| MAPT | LINC02106 | 0.619909413 | 4.20E-45 | positive |
| MYLK | LINC02106 | 0.808659561 | 1.64E-96 | positive |
| PCDH7 | LINC02106 | 0.614340207 | 4.13E-44 | positive |
| PRKAA2 | LINC02106 | 0.604115409 | 2.44E-42 | positive |
| PRNP | LINC02106 | 0.661584092 | 3.29E-53 | positive |
| SRF | LINC02106 | 0.625351908 | 4.31E-46 | positive |
| STMN2 | LINC02106 | 0.400670757 | 2.56E-17 | positive |
| TUBA1A | LINC02106 | 0.720034631 | 4.77E-67 | positive |
| VCL | LINC02106 | 0.701636501 | 2.50E-62 | positive |
| GZMB | LINC01943 | 0.572346517 | 3.17E-37 | positive |
| MLANA | ACAP2-IT1 | 0.463995791 | 2.19E-23 | positive |
| PIKFYVE | ACAP2-IT1 | 0.52429056 | 1.81E-30 | positive |
| RASA2 | ACAP2-IT1 | 0.596404347 | 4.80E-41 | positive |
| MLANA | AC006017.1 | 0.446664041 | 1.35E-21 | positive |
| RASA2 | AC006017.1 | 0.449000462 | 7.83E-22 | positive |
| MAP1LC3A | AC120036.4 | 0.425712411 | 1.44E-19 | positive |
| MYLK | AC120036.4 | 0.422656198 | 2.78E-19 | positive |
| PCDH7 | AC120036.4 | 0.424514191 | 1.87E-19 | positive |
| PRKAA2 | AC120036.4 | 0.421552916 | 3.52E-19 | positive |
| AR | AC018521.6 | 0.507416883 | 2.42E-28 | positive |
| MAPT | AC018521.6 | 0.485243631 | 1.01E-25 | positive |
| MYLK | AC018521.6 | 0.603257377 | 3.41E-42 | positive |
| PCDH7 | AC018521.6 | 0.473501103 | 2.06E-24 | positive |
| PRKAA2 | AC018521.6 | 0.485591559 | 9.19E-26 | positive |
| PRNP | AC018521.6 | 0.446496566 | 1.40E-21 | positive |
| SRF | AC018521.6 | 0.471175568 | 3.70E-24 | positive |
| TUBA1A | AC018521.6 | 0.431948908 | 3.71E-20 | positive |
| VCL | AC018521.6 | 0.53515245 | 6.67E-32 | positive |
| MLANA | AC016590.2 | 0.449222537 | 7.44E-22 | positive |
| CTSL | AC006033.2 | 0.43103028 | 4.55E-20 | positive |
| CTSS | AC006033.2 | 0.529406004 | 3.88E-31 | positive |
| CYBB | AC006033.2 | 0.714167291 | 1.68E-65 | positive |
| GZMB | AC006033.2 | 0.448152608 | 9.54E-22 | positive |
| MLANA | AC009686.1 | 0.461474821 | 4.04E-23 | positive |
| MLANA | LIX1L-AS1 | 0.409015918 | 4.78E-18 | positive |
| MYL2 | LIX1L-AS1 | 0.412897016 | 2.16E-18 | positive |
| RNF146 | LIX1L-AS1 | 0.404090082 | 1.29E-17 | positive |
| RASA2 | MUC20-OT1 | 0.490198125 | 2.72E-26 | positive |
| MLANA | AC022558.3 | 0.414807603 | 1.45E-18 | positive |
| RASA2 | AC022558.3 | 0.42664013 | 1.18E-19 | positive |
| RASA2 | ARAP1-AS2 | 0.405264277 | 1.02E-17 | positive |
| TP63 | AC103563.2 | 0.445253258 | 1.86E-21 | positive |
| MLANA | AL031666.2 | 0.408540973 | 5.26E-18 | positive |
| AR | AL132642.1 | 0.611072428 | 1.55E-43 | positive |
| CAV1 | AL132642.1 | 0.420696169 | 4.22E-19 | positive |
| ITGB1 | AL132642.1 | 0.549933246 | 6.14E-34 | positive |
| MAPT | AL132642.1 | 0.54627236 | 2.00E-33 | positive |
| MYLK | AL132642.1 | 0.85391271 | 2.33E-118 | positive |
| PCDH7 | AL132642.1 | 0.624612096 | 5.89E-46 | positive |
| PRKAA2 | AL132642.1 | 0.687860219 | 5.04E-59 | positive |
| PRNP | AL132642.1 | 0.65817189 | 1.70E-52 | positive |
| RNF146 | AL132642.1 | 0.430889514 | 4.69E-20 | positive |
| ROCK1 | AL132642.1 | 0.477802962 | 6.92E-25 | positive |
| SRF | AL132642.1 | 0.646749256 | 3.54E-50 | positive |
| TUBA1A | AL132642.1 | 0.668154257 | 1.32E-54 | positive |
| VCL | AL132642.1 | 0.757956323 | 4.36E-78 | positive |
| MLANA | AL080317.2 | 0.425799826 | 1.42E-19 | positive |
| MYL2 | AC097376.2 | 0.453423038 | 2.78E-22 | positive |
| MLANA | FTX | 0.481121481 | 2.95E-25 | positive |
| RASA2 | FTX | 0.441851472 | 4.06E-21 | positive |
| RNF146 | FTX | 0.55389421 | 1.68E-34 | positive |
| ROCK1 | FTX | 0.446760542 | 1.32E-21 | positive |
| AR | Z99289.1 | 0.569671422 | 8.08E-37 | positive |
| ITGB1 | Z99289.1 | 0.559151765 | 2.93E-35 | positive |
| KIF2C | Z99289.1 | -0.414373955 | 1.59E-18 | negative |
| MAPT | Z99289.1 | 0.426765963 | 1.15E-19 | positive |
| MYLK | Z99289.1 | 0.713338495 | 2.75E-65 | positive |
| PCDH7 | Z99289.1 | 0.531129121 | 2.29E-31 | positive |
| PRKAA2 | Z99289.1 | 0.574029567 | 1.76E-37 | positive |
| PRNP | Z99289.1 | 0.56371121 | 6.28E-36 | positive |
| RNF146 | Z99289.1 | 0.504958402 | 4.82E-28 | positive |
| ROCK1 | Z99289.1 | 0.505143285 | 4.58E-28 | positive |
| SRF | Z99289.1 | 0.479260527 | 4.76E-25 | positive |
| TUBA1A | Z99289.1 | 0.621238802 | 2.42E-45 | positive |
| VCL | Z99289.1 | 0.693781258 | 2.02E-60 | positive |
| VIM | Z99289.1 | 0.401327525 | 2.24E-17 | positive |
| RASA2 | AC025034.1 | 0.489501385 | 3.27E-26 | positive |
| KIF2C | AC091057.1 | 0.487235407 | 5.96E-26 | positive |
| MAD2L1 | AC091057.1 | 0.417172722 | 8.86E-19 | positive |
| CALB2 | AL365356.5 | 0.630816039 | 4.18E-47 | positive |
| CDH3 | AL365356.5 | 0.401472805 | 2.18E-17 | positive |
| MLANA | AC004771.1 | 0.410243975 | 3.72E-18 | positive |
| RASA2 | AC004771.1 | 0.468018337 | 8.13E-24 | positive |
| MLANA | AC090772.1 | 0.493152092 | 1.23E-26 | positive |
| RASA2 | AC090772.1 | 0.450868945 | 5.06E-22 | positive |
| MTMR7 | AC010247.2 | 0.590662943 | 4.19E-40 | positive |
| MTUS2 | AC010247.2 | 0.740948757 | 6.76E-73 | positive |
| MTMR7 | HCG18 | 0.43854552 | 8.57E-21 | positive |
| RASA2 | PTOV1-AS2 | 0.424332962 | 1.94E-19 | positive |
| MLANA | AC090617.4 | 0.481496635 | 2.67E-25 | positive |
| RASA2 | AC090617.4 | 0.41700268 | 9.18E-19 | positive |
| RASA2 | AC004253.1 | 0.470739056 | 4.13E-24 | positive |
| ROCK1 | AC004253.1 | 0.432568341 | 3.24E-20 | positive |
| AURKA | AC108451.2 | 0.438114212 | 9.44E-21 | positive |
| MAPT | MRPS30-DT | 0.412301978 | 2.44E-18 | positive |
| NTS | MRPS30-DT | 0.90915415 | 4.59E-158 | positive |
| SNCA | MRPS30-DT | 0.440457842 | 5.56E-21 | positive |
| CALB2 | AC245041.2 | 0.428122372 | 8.57E-20 | positive |
| CDH3 | AC245041.2 | 0.431522633 | 4.08E-20 | positive |
| KRT7 | AC245041.2 | 0.517541742 | 1.32E-29 | positive |
| TP63 | AC245041.2 | 0.43244249 | 3.33E-20 | positive |
| AR | AC104825.1 | 0.609960515 | 2.41E-43 | positive |
| CDC20 | AC104825.1 | -0.413606461 | 1.86E-18 | negative |
| ITGB1 | AC104825.1 | 0.407458672 | 6.56E-18 | positive |
| KIF2C | AC104825.1 | -0.456892356 | 1.22E-22 | negative |
| MAD2L1 | AC104825.1 | -0.422160104 | 3.09E-19 | negative |
| MAPT | AC104825.1 | 0.502966953 | 8.40E-28 | positive |
| MYLK | AC104825.1 | 0.726447195 | 8.77E-69 | positive |
| PCDH7 | AC104825.1 | 0.570077424 | 7.01E-37 | positive |
| PRKAA2 | AC104825.1 | 0.614366322 | 4.09E-44 | positive |
| PRNP | AC104825.1 | 0.577742394 | 4.70E-38 | positive |
| RARB | AC104825.1 | 0.423166887 | 2.49E-19 | positive |
| RNF146 | AC104825.1 | 0.412433718 | 2.37E-18 | positive |
| ROCK1 | AC104825.1 | 0.400874018 | 2.45E-17 | positive |
| SRF | AC104825.1 | 0.495157854 | 7.16E-27 | positive |
| TUBA1A | AC104825.1 | 0.66516064 | 5.77E-54 | positive |
| VCL | AC104825.1 | 0.652075475 | 3.02E-51 | positive |
| MLANA | AC055855.1 | 0.432048244 | 3.63E-20 | positive |
| RASA2 | AC055855.1 | 0.460369671 | 5.28E-23 | positive |
| RNF146 | AC010201.2 | 0.407615411 | 6.35E-18 | positive |
| ROCK1 | AC010201.2 | 0.418970125 | 6.07E-19 | positive |
| MLANA | AL513365.2 | 0.572272074 | 3.26E-37 | positive |
| PIKFYVE | AL513365.2 | 0.449160186 | 7.55E-22 | positive |
| RASA2 | AL513365.2 | 0.49668861 | 4.73E-27 | positive |
| SPRN | AL513365.2 | 0.417577851 | 8.14E-19 | positive |
| RASA2 | AC092375.2 | 0.423451982 | 2.35E-19 | positive |
| MLANA | AL049637.1 | 0.441365819 | 4.53E-21 | positive |
| MLANA | AL442003.1 | 0.527734065 | 6.43E-31 | positive |
| PIKFYVE | AL442003.1 | 0.434903095 | 1.93E-20 | positive |
| RASA2 | AL442003.1 | 0.506807592 | 2.87E-28 | positive |
| CALB2 | LINC00460 | 0.462511078 | 3.14E-23 | positive |
| KRT7 | LINC00460 | 0.523190844 | 2.51E-30 | positive |
| MLANA | AC099811.1 | 0.479555388 | 4.41E-25 | positive |
| RASA2 | AC099811.1 | 0.4329632 | 2.97E-20 | positive |
| MLANA | AC007038.1 | 0.49826615 | 3.07E-27 | positive |
| PIKFYVE | AC007038.1 | 0.404514554 | 1.19E-17 | positive |
| RASA2 | AC007038.1 | 0.483279578 | 1.68E-25 | positive |
| AR | AL596244.1 | 0.590444067 | 4.55E-40 | positive |
| CAV1 | AL596244.1 | 0.426349603 | 1.26E-19 | positive |
| ITGB1 | AL596244.1 | 0.522589523 | 3.00E-30 | positive |
| MAPT | AL596244.1 | 0.569636577 | 8.18E-37 | positive |
| MYLK | AL596244.1 | 0.813693663 | 1.22E-98 | positive |
| PCDH7 | AL596244.1 | 0.591339303 | 3.25E-40 | positive |
| PRKAA2 | AL596244.1 | 0.634120582 | 9.98E-48 | positive |
| PRNP | AL596244.1 | 0.658091151 | 1.77E-52 | positive |
| RNF146 | AL596244.1 | 0.42367224 | 2.24E-19 | positive |
| SRF | AL596244.1 | 0.615693937 | 2.38E-44 | positive |
| TUBA1A | AL596244.1 | 0.688319416 | 3.94E-59 | positive |
| VCL | AL596244.1 | 0.722877523 | 8.22E-68 | positive |
| RASA2 | AL157786.1 | 0.440542783 | 5.46E-21 | positive |
| MLANA | AL117350.1 | 0.422291569 | 3.01E-19 | positive |
| MLANA | NCBP2-AS1 | 0.403670842 | 1.41E-17 | positive |
| RASA2 | NCBP2-AS1 | 0.415019979 | 1.39E-18 | positive |
| MYLK | RGMB-AS1 | 0.43765824 | 1.05E-20 | positive |
| TUBA1A | RGMB-AS1 | 0.430452788 | 5.16E-20 | positive |
| MLANA | AC011462.4 | 0.418677447 | 6.46E-19 | positive |
| RASA2 | AC011462.4 | 0.412304282 | 2.44E-18 | positive |
| MLANA | AC087501.2 | 0.415457781 | 1.27E-18 | positive |
| CEBPB | CYTOR | 0.43604721 | 1.50E-20 | positive |
| TUBA1A | AC009506.1 | 0.413166886 | 2.04E-18 | positive |
| MAP1LC3A | AC009414.2 | 0.406624322 | 7.76E-18 | positive |
| TUBA1A | AC009414.2 | 0.401471762 | 2.18E-17 | positive |
| MTMR7 | AC009974.1 | 0.40182159 | 2.03E-17 | positive |
| AR | A2M-AS1 | 0.736579196 | 1.25E-71 | positive |
| CAV1 | A2M-AS1 | 0.468962523 | 6.43E-24 | positive |
| ITGB1 | A2M-AS1 | 0.512313537 | 6.00E-29 | positive |
| MAPT | A2M-AS1 | 0.620485954 | 3.31E-45 | positive |
| MYLK | A2M-AS1 | 0.867662535 | 1.65E-126 | positive |
| PCDH7 | A2M-AS1 | 0.564189065 | 5.33E-36 | positive |
| PRKAA2 | A2M-AS1 | 0.701745064 | 2.35E-62 | positive |
| PRNP | A2M-AS1 | 0.766740138 | 6.13E-81 | positive |
| RNF146 | A2M-AS1 | 0.456956223 | 1.20E-22 | positive |
| ROCK1 | A2M-AS1 | 0.416846357 | 9.49E-19 | positive |
| S100B | A2M-AS1 | 0.412979636 | 2.12E-18 | positive |
| SRF | A2M-AS1 | 0.601648657 | 6.38E-42 | positive |
| TUBA1A | A2M-AS1 | 0.775675766 | 5.68E-84 | positive |
| VCL | A2M-AS1 | 0.734496504 | 4.94E-71 | positive |
| TM9SF4 | LINC01719 | 0.400473916 | 2.66E-17 | positive |
| NTS | AC021028.1 | 0.429362943 | 6.55E-20 | positive |
| AR | AC008808.2 | 0.675750198 | 2.87E-56 | positive |
| CDH1 | AC008808.2 | -0.430405512 | 5.21E-20 | negative |
| KIF2C | AC008808.2 | -0.477692332 | 7.12E-25 | negative |
| MAD2L1 | AC008808.2 | -0.453455475 | 2.76E-22 | negative |
| MAPT | AC008808.2 | 0.508418383 | 1.82E-28 | positive |
| MYLK | AC008808.2 | 0.703824244 | 7.19E-63 | positive |
| PCDH7 | AC008808.2 | 0.524017218 | 1.96E-30 | positive |
| PRKAA2 | AC008808.2 | 0.483026289 | 1.80E-25 | positive |
| PRNP | AC008808.2 | 0.639085334 | 1.12E-48 | positive |
| RNF146 | AC008808.2 | 0.419716772 | 5.19E-19 | positive |
| S100B | AC008808.2 | 0.42323599 | 2.46E-19 | positive |
| SRF | AC008808.2 | 0.505297941 | 4.38E-28 | positive |
| STMN2 | AC008808.2 | 0.440210678 | 5.88E-21 | positive |
| TUBA1A | AC008808.2 | 0.727753541 | 3.83E-69 | positive |
| VCL | AC008808.2 | 0.613141262 | 6.72E-44 | positive |
| VIM | AC008808.2 | 0.486797913 | 6.69E-26 | positive |
| MLANA | SIDT1-AS1 | 0.416577757 | 1.00E-18 | positive |
| CDKN2A | G2E3-AS1 | 0.412973976 | 2.12E-18 | positive |
| CDC20 | AC006329.1 | 0.42334452 | 2.40E-19 | positive |
| MYC | AC006329.1 | 0.59575551 | 6.14E-41 | positive |
| MLANA | AL139120.1 | 0.443809086 | 2.60E-21 | positive |
| RASA2 | AL139120.1 | 0.464769078 | 1.81E-23 | positive |
| RND3 | LINC02487 | 0.444462796 | 2.23E-21 | positive |
| TP63 | LINC02487 | 0.491167839 | 2.10E-26 | positive |
| MLANA | AC010976.1 | 0.439385818 | 7.09E-21 | positive |
| RASA2 | AC010976.1 | 0.465742868 | 1.43E-23 | positive |
| NTS | GAS5 | 0.628485076 | 1.14E-46 | positive |
| MTMR7 | WASHC5-AS1 | 0.538014363 | 2.74E-32 | positive |
| MTUS2 | WASHC5-AS1 | 0.56168486 | 1.25E-35 | positive |
| MLANA | AC114939.1 | 0.417563914 | 8.16E-19 | positive |
| RASA2 | AC114939.1 | 0.411569952 | 2.83E-18 | positive |
| MLANA | CTBP1-AS | 0.498362557 | 2.99E-27 | positive |
| RASA2 | CTBP1-AS | 0.450689147 | 5.28E-22 | positive |
| CDKN2A | LINC01239 | 0.412640488 | 2.27E-18 | positive |
| ATG5 | PVT1 | 0.487682101 | 5.30E-26 | positive |
| MYC | PVT1 | 0.473057524 | 2.31E-24 | positive |
| MLANA | PSMA3-AS1 | 0.455176034 | 1.83E-22 | positive |
| RASA2 | PSMA3-AS1 | 0.514857476 | 2.89E-29 | positive |
| RNF146 | PSMA3-AS1 | 0.481104035 | 2.96E-25 | positive |
| ROCK1 | PSMA3-AS1 | 0.430443678 | 5.17E-20 | positive |
| CYBB | AP002954.1 | 0.54374764 | 4.49E-33 | positive |
| GZMB | AP002954.1 | 0.413748551 | 1.81E-18 | positive |
| AR | U91328.1 | 0.452013847 | 3.87E-22 | positive |
| MAPT | U91328.1 | 0.443475626 | 2.80E-21 | positive |
| MYLK | U91328.1 | 0.481914428 | 2.40E-25 | positive |
| PRKAA2 | U91328.1 | 0.489881141 | 2.96E-26 | positive |
| PRNP | U91328.1 | 0.431810095 | 3.83E-20 | positive |
| SRF | U91328.1 | 0.418889671 | 6.18E-19 | positive |
| TUBA1A | U91328.1 | 0.400563871 | 2.61E-17 | positive |
| RASA2 | MCCC1-AS1 | 0.492589772 | 1.43E-26 | positive |
| MTMR7 | AC002511.1 | 0.472362825 | 2.75E-24 | positive |
| MTUS2 | AC002511.1 | 0.496817342 | 4.56E-27 | positive |
| AR | AC011472.1 | 0.645225634 | 7.09E-50 | positive |
| CAV1 | AC011472.1 | 0.407425254 | 6.60E-18 | positive |
| MAPT | AC011472.1 | 0.638155755 | 1.69E-48 | positive |
| MYLK | AC011472.1 | 0.766083028 | 1.01E-80 | positive |
| PCDH7 | AC011472.1 | 0.531258102 | 2.21E-31 | positive |
| PRKAA2 | AC011472.1 | 0.608851073 | 3.76E-43 | positive |
| PRNP | AC011472.1 | 0.589573129 | 6.29E-40 | positive |
| RNF146 | AC011472.1 | 0.460428061 | 5.21E-23 | positive |
| ROCK1 | AC011472.1 | 0.46220697 | 3.39E-23 | positive |
| SRF | AC011472.1 | 0.591113437 | 3.54E-40 | positive |
| TUBA1A | AC011472.1 | 0.658184692 | 1.69E-52 | positive |
| VCL | AC011472.1 | 0.697895078 | 2.06E-61 | positive |
| DIAPH1 | AC090114.2 | -0.41740083 | 8.45E-19 | negative |
| MYLK | AC090114.2 | 0.405880701 | 9.02E-18 | positive |
| AR | AC005165.1 | 0.453026205 | 3.05E-22 | positive |
| ARHGAP36 | AC005165.1 | 0.536646384 | 4.20E-32 | positive |
| MYLK | AC005165.1 | 0.55718727 | 5.65E-35 | positive |
| PCDH7 | AC005165.1 | 0.528501618 | 5.10E-31 | positive |
| PRKAA2 | AC005165.1 | 0.418244824 | 7.08E-19 | positive |
| PRNP | AC005165.1 | 0.438555078 | 8.55E-21 | positive |
| SRF | AC005165.1 | 0.460126958 | 5.60E-23 | positive |
| TUBA1A | AC005165.1 | 0.44612091 | 1.53E-21 | positive |
| VCL | AC005165.1 | 0.446579074 | 1.37E-21 | positive |
| MAPT | AL359504.2 | 0.482746022 | 1.93E-25 | positive |
| PRKAA2 | AL359504.2 | 0.522723316 | 2.88E-30 | positive |
| PRNP | AL359504.2 | 0.420195218 | 4.69E-19 | positive |
| TUBA1A | AL359504.2 | 0.41827902 | 7.02E-19 | positive |
| RASA2 | AP002907.1 | 0.469089352 | 6.23E-24 | positive |
| MLANA | AC092902.2 | 0.412035938 | 2.57E-18 | positive |
| AR | AL031587.3 | 0.516776713 | 1.65E-29 | positive |
| CAV1 | AL031587.3 | 0.42149065 | 3.56E-19 | positive |
| ITGB1 | AL031587.3 | 0.424897286 | 1.72E-19 | positive |
| MAPT | AL031587.3 | 0.515338319 | 2.51E-29 | positive |
| MYLK | AL031587.3 | 0.679555697 | 4.04E-57 | positive |
| PCDH7 | AL031587.3 | 0.477782066 | 6.96E-25 | positive |
| PRKAA2 | AL031587.3 | 0.578830108 | 3.19E-38 | positive |
| PRNP | AL031587.3 | 0.603234538 | 3.44E-42 | positive |
| SRF | AL031587.3 | 0.520807738 | 5.08E-30 | positive |
| TUBA1A | AL031587.3 | 0.643589989 | 1.49E-49 | positive |
| VCL | AL031587.3 | 0.628572095 | 1.10E-46 | positive |
| NTS | FZD10-DT | 0.70687786 | 1.23E-63 | positive |
| RASA2 | AC006480.2 | 0.402922125 | 1.63E-17 | positive |
| MLANA | LINC00216 | 0.463542853 | 2.44E-23 | positive |
| RASA2 | LINC00216 | 0.458842372 | 7.64E-23 | positive |
| RASA2 | Z98884.2 | 0.408646903 | 5.15E-18 | positive |
| RASA2 | AC023825.2 | 0.423559234 | 2.29E-19 | positive |
| ATG5 | Z98200.1 | 0.441515153 | 4.38E-21 | positive |
| MTMR7 | AL590705.3 | 0.520886352 | 4.96E-30 | positive |
| MTUS2 | AL590705.3 | 0.537537745 | 3.18E-32 | positive |
| ROCK1 | AC104170.2 | 0.409316992 | 4.49E-18 | positive |
| AR | AP001065.1 | 0.592979095 | 1.76E-40 | positive |
| MAPT | AP001065.1 | 0.526159587 | 1.03E-30 | positive |
| MYLK | AP001065.1 | 0.644331865 | 1.06E-49 | positive |
| PRKAA2 | AP001065.1 | 0.495362738 | 6.78E-27 | positive |
| PRNP | AP001065.1 | 0.521275689 | 4.42E-30 | positive |
| SRF | AP001065.1 | 0.483813638 | 1.46E-25 | positive |
| TUBA1A | AP001065.1 | 0.492249372 | 1.57E-26 | positive |
| VCL | AP001065.1 | 0.536813433 | 3.98E-32 | positive |
| GZMB | AL157871.2 | 0.411290679 | 3.00E-18 | positive |
| MAPT | SOCS2-AS1 | 0.402046114 | 1.94E-17 | positive |
| NTS | SOCS2-AS1 | 0.564397643 | 4.97E-36 | positive |
| MLANA | AL133245.1 | 0.524728926 | 1.59E-30 | positive |
| ROCK1 | AL133245.1 | 0.432904495 | 3.01E-20 | positive |
| RASA2 | AC068790.7 | 0.506257204 | 3.35E-28 | positive |
| RASA2 | AC096586.2 | 0.438798245 | 8.09E-21 | positive |
| MYL2 | AC006273.1 | 0.401826352 | 2.03E-17 | positive |
| MLANA | AC016027.3 | 0.415812349 | 1.18E-18 | positive |
| MLANA | AC127024.6 | 0.432835223 | 3.06E-20 | positive |
| RASA2 | AC127024.6 | 0.41515019 | 1.35E-18 | positive |
| SFTPB | AL121820.1 | 0.543342109 | 5.11E-33 | positive |
| MLANA | AL078581.3 | 0.469605832 | 5.48E-24 | positive |
| RASA2 | AL078581.3 | 0.439567357 | 6.80E-21 | positive |
| AR | AC090044.1 | 0.685298947 | 1.98E-58 | positive |
| MAPT | AC090044.1 | 0.585799882 | 2.54E-39 | positive |
| MYLK | AC090044.1 | 0.655165221 | 7.08E-52 | positive |
| PCDH7 | AC090044.1 | 0.553518135 | 1.90E-34 | positive |
| PRKAA2 | AC090044.1 | 0.524024561 | 1.96E-30 | positive |
| PRNP | AC090044.1 | 0.55557482 | 9.65E-35 | positive |
| SRF | AC090044.1 | 0.491795146 | 1.77E-26 | positive |
| TUBA1A | AC090044.1 | 0.48961724 | 3.17E-26 | positive |
| VCL | AC090044.1 | 0.585967013 | 2.39E-39 | positive |
| AR | PGM5-AS1 | 0.656025722 | 4.72E-52 | positive |
| CAV1 | PGM5-AS1 | 0.495285935 | 6.92E-27 | positive |
| ITGB1 | PGM5-AS1 | 0.486249447 | 7.73E-26 | positive |
| MAPT | PGM5-AS1 | 0.644049203 | 1.21E-49 | positive |
| MYLK | PGM5-AS1 | 0.887552694 | 4.47E-140 | positive |
| PCDH7 | PGM5-AS1 | 0.567140265 | 1.94E-36 | positive |
| PRKAA2 | PGM5-AS1 | 0.629823826 | 6.41E-47 | positive |
| PRNP | PGM5-AS1 | 0.776258708 | 3.56E-84 | positive |
| S100B | PGM5-AS1 | 0.443023862 | 3.11E-21 | positive |
| SRF | PGM5-AS1 | 0.669734276 | 6.00E-55 | positive |
| TUBA1A | PGM5-AS1 | 0.803816881 | 1.60E-94 | positive |
| VCL | PGM5-AS1 | 0.751109518 | 6.02E-76 | positive |
| NTS | HOXC-AS1 | 0.433501894 | 2.64E-20 | positive |
| STMN2 | AC139792.1 | 0.428353636 | 8.15E-20 | positive |
| AR | ADAMTS9-AS2 | 0.767834586 | 2.65E-81 | positive |
| CAV1 | ADAMTS9-AS2 | 0.461287853 | 4.23E-23 | positive |
| CDH1 | ADAMTS9-AS2 | -0.404662021 | 1.15E-17 | negative |
| ITGB1 | ADAMTS9-AS2 | 0.522469464 | 3.11E-30 | positive |
| KIF2C | ADAMTS9-AS2 | -0.443732661 | 2.64E-21 | negative |
| MAD2L1 | ADAMTS9-AS2 | -0.403168731 | 1.55E-17 | negative |
| MAPT | ADAMTS9-AS2 | 0.659513249 | 8.94E-53 | positive |
| MYLK | ADAMTS9-AS2 | 0.875833818 | 8.47E-132 | positive |
| PCDH7 | ADAMTS9-AS2 | 0.667630927 | 1.71E-54 | positive |
| PRKAA2 | ADAMTS9-AS2 | 0.700799235 | 4.03E-62 | positive |
| PRNP | ADAMTS9-AS2 | 0.733908912 | 7.25E-71 | positive |
| RNF146 | ADAMTS9-AS2 | 0.479411602 | 4.58E-25 | positive |
| ROCK1 | ADAMTS9-AS2 | 0.477314337 | 7.84E-25 | positive |
| SRF | ADAMTS9-AS2 | 0.654269559 | 1.08E-51 | positive |
| TUBA1A | ADAMTS9-AS2 | 0.725782676 | 1.33E-68 | positive |
| VCL | ADAMTS9-AS2 | 0.799731975 | 6.91E-93 | positive |
| CTSB | AC025171.1 | 0.421744482 | 3.38E-19 | positive |
| AR | AL136084.3 | 0.57919259 | 2.80E-38 | positive |
| CAV1 | AL136084.3 | 0.46105104 | 4.48E-23 | positive |
| ITGB1 | AL136084.3 | 0.594807811 | 8.81E-41 | positive |
| MAPT | AL136084.3 | 0.538942865 | 2.05E-32 | positive |
| MYLK | AL136084.3 | 0.889023566 | 3.52E-141 | positive |
| PCDH7 | AL136084.3 | 0.609215566 | 3.25E-43 | positive |
| PRKAA2 | AL136084.3 | 0.664675846 | 7.32E-54 | positive |
| PRNP | AL136084.3 | 0.704788051 | 4.13E-63 | positive |
| SRF | AL136084.3 | 0.711617313 | 7.66E-65 | positive |
| TUBA1A | AL136084.3 | 0.768068768 | 2.21E-81 | positive |
| VCL | AL136084.3 | 0.759154619 | 1.81E-78 | positive |
| TP63 | C5orf66-AS1 | 0.583991926 | 4.92E-39 | positive |
| AR | AC104117.3 | 0.670956102 | 3.25E-55 | positive |
| CDH1 | AC104117.3 | -0.421862484 | 3.29E-19 | negative |
| ITGB1 | AC104117.3 | 0.46509537 | 1.67E-23 | positive |
| KIF2C | AC104117.3 | -0.439521662 | 6.88E-21 | negative |
| MAD2L1 | AC104117.3 | -0.451366764 | 4.51E-22 | negative |
| MAPT | AC104117.3 | 0.532891144 | 1.34E-31 | positive |
| MYLK | AC104117.3 | 0.666553429 | 2.91E-54 | positive |
| PCDH7 | AC104117.3 | 0.505113315 | 4.62E-28 | positive |
| PRKAA2 | AC104117.3 | 0.511013247 | 8.71E-29 | positive |
| PRNP | AC104117.3 | 0.586757325 | 1.78E-39 | positive |
| RNF146 | AC104117.3 | 0.414096397 | 1.68E-18 | positive |
| SRF | AC104117.3 | 0.504974194 | 4.80E-28 | positive |
| TUBA1A | AC104117.3 | 0.687018178 | 7.91E-59 | positive |
| VCL | AC104117.3 | 0.619084415 | 5.92E-45 | positive |
| VIM | AC104117.3 | 0.435201899 | 1.81E-20 | positive |
| MLANA | AC092436.4 | 0.477622527 | 7.24E-25 | positive |
| RASA2 | AC092436.4 | 0.459930185 | 5.88E-23 | positive |
| MLANA | AL022328.1 | 0.478515922 | 5.76E-25 | positive |
| RASA2 | AL022328.1 | 0.49035817 | 2.60E-26 | positive |
| MLANA | AC127024.4 | 0.507519654 | 2.35E-28 | positive |
| ROCK1 | AC127024.4 | 0.404261013 | 1.25E-17 | positive |
| RAC1 | LINC01133 | 0.42607128 | 1.34E-19 | positive |
| MLANA | AC114760.2 | 0.486063879 | 8.12E-26 | positive |
| RASA2 | AC114760.2 | 0.510953734 | 8.86E-29 | positive |
| CDKN2A | AL606970.1 | 0.428070835 | 8.67E-20 | positive |
| NTS | AC092794.2 | 0.519248671 | 8.03E-30 | positive |
| AR | AC100803.2 | 0.406490224 | 7.98E-18 | positive |
| MLANA | ZNF460-AS1 | 0.405931957 | 8.93E-18 | positive |
| RAC1 | MPRIP-AS1 | 0.457299678 | 1.11E-22 | positive |
| RASA2 | AC006378.1 | 0.460286527 | 5.39E-23 | positive |
| MLANA | MIR600HG | 0.45830778 | 8.68E-23 | positive |
| MLANA | HCG25 | 0.47335746 | 2.14E-24 | positive |
| RASA2 | HCG25 | 0.42132069 | 3.69E-19 | positive |
| ROCK1 | HCG25 | 0.423141928 | 2.51E-19 | positive |
| CXCL8 | AC112496.1 | 0.418319938 | 6.96E-19 | positive |
| IL6 | AC112496.1 | 0.511060567 | 8.59E-29 | positive |
| PDPN | AC112496.1 | 0.532116675 | 1.70E-31 | positive |
| AR | LINC02256 | 0.570440484 | 6.18E-37 | positive |
| KIF2C | LINC02256 | -0.451155198 | 4.74E-22 | negative |
| MAD2L1 | LINC02256 | -0.413938616 | 1.74E-18 | negative |
| MAPT | LINC02256 | 0.525699986 | 1.19E-30 | positive |
| MYLK | LINC02256 | 0.645603255 | 5.97E-50 | positive |
| PCDH7 | LINC02256 | 0.5476833 | 1.27E-33 | positive |
| PRKAA2 | LINC02256 | 0.492869987 | 1.33E-26 | positive |
| PRNP | LINC02256 | 0.532908726 | 1.33E-31 | positive |
| SRF | LINC02256 | 0.512028327 | 6.51E-29 | positive |
| TUBA1A | LINC02256 | 0.572992574 | 2.53E-37 | positive |
| VCL | LINC02256 | 0.585610507 | 2.72E-39 | positive |
| AR | NALT1 | 0.730097921 | 8.57E-70 | positive |
| CAV1 | NALT1 | 0.482433272 | 2.10E-25 | positive |
| ITGB1 | NALT1 | 0.464905082 | 1.75E-23 | positive |
| MAPT | NALT1 | 0.691664247 | 6.43E-60 | positive |
| MYLK | NALT1 | 0.90269722 | 2.99E-152 | positive |
| PCDH7 | NALT1 | 0.552222203 | 2.91E-34 | positive |
| PRKAA2 | NALT1 | 0.69898539 | 1.12E-61 | positive |
| PRNP | NALT1 | 0.758907894 | 2.17E-78 | positive |
| S100B | NALT1 | 0.457147494 | 1.15E-22 | positive |
| SRF | NALT1 | 0.706717154 | 1.35E-63 | positive |
| TUBA1A | NALT1 | 0.811560668 | 9.92E-98 | positive |
| VCL | NALT1 | 0.749032413 | 2.60E-75 | positive |
| MLANA | AC007216.4 | 0.427327708 | 1.02E-19 | positive |
| ERI3 | AC108010.1 | -0.421363312 | 3.66E-19 | negative |
| MLANA | AC108010.1 | 0.480361474 | 3.58E-25 | positive |
| PIKFYVE | AC108010.1 | 0.4764614 | 9.74E-25 | positive |
| RASA2 | AC108010.1 | 0.47365744 | 1.98E-24 | positive |
| CTSS | AC083949.1 | 0.444468347 | 2.23E-21 | positive |
| CYBB | AC083949.1 | 0.587129887 | 1.56E-39 | positive |
| GZMB | AC083949.1 | 0.403924927 | 1.34E-17 | positive |
| AR | FENDRR | 0.741382434 | 5.04E-73 | positive |
| CAV1 | FENDRR | 0.481410987 | 2.73E-25 | positive |
| ITGB1 | FENDRR | 0.568138866 | 1.37E-36 | positive |
| KIF2C | FENDRR | -0.44361624 | 2.71E-21 | negative |
| MAD2L1 | FENDRR | -0.413028742 | 2.10E-18 | negative |
| MAPT | FENDRR | 0.635185467 | 6.26E-48 | positive |
| MYLK | FENDRR | 0.9313845 | 4.96E-182 | positive |
| PCDH7 | FENDRR | 0.641166558 | 4.43E-49 | positive |
| PRKAA2 | FENDRR | 0.703069497 | 1.11E-62 | positive |
| PRNP | FENDRR | 0.781458614 | 5.19E-86 | positive |
| RNF146 | FENDRR | 0.44231186 | 3.65E-21 | positive |
| ROCK1 | FENDRR | 0.429399241 | 6.49E-20 | positive |
| S100B | FENDRR | 0.410077476 | 3.85E-18 | positive |
| SRF | FENDRR | 0.69317399 | 2.82E-60 | positive |
| STMN2 | FENDRR | 0.413430222 | 1.93E-18 | positive |
| TUBA1A | FENDRR | 0.824901914 | 1.27E-103 | positive |
| VCL | FENDRR | 0.821980407 | 2.74E-102 | positive |
| CALB2 | LINC02156 | 0.456894214 | 1.22E-22 | positive |
| MLANA | ANKRD44-IT1 | 0.448378996 | 9.05E-22 | positive |
| RASA2 | ANKRD44-IT1 | 0.437404066 | 1.11E-20 | positive |
| RASA2 | AL139022.1 | 0.421705073 | 3.40E-19 | positive |
| ROCK1 | AL133243.2 | 0.407703858 | 6.24E-18 | positive |
| RARB | AC254633.1 | 0.490007357 | 2.86E-26 | positive |
| MLANA | AC005072.1 | 0.414715086 | 1.48E-18 | positive |
| ROCK1 | AC005072.1 | 0.441366435 | 4.53E-21 | positive |
| RASA2 | AL731567.1 | 0.420247764 | 4.64E-19 | positive |
| MLANA | AC063965.1 | 0.412457261 | 2.36E-18 | positive |
| RASA2 | AC063965.1 | 0.5047652 | 5.09E-28 | positive |
| MLANA | AC025171.4 | 0.419325057 | 5.64E-19 | positive |
| RASA2 | AC025171.4 | 0.409826003 | 4.05E-18 | positive |
| AR | AP001922.6 | 0.454229384 | 2.30E-22 | positive |
| IL6 | AP001107.3 | 0.42757898 | 9.65E-20 | positive |
| WT1 | AP001107.3 | 0.603626688 | 2.95E-42 | positive |
| MLANA | AC018607.1 | 0.501523454 | 1.25E-27 | positive |
| RASA2 | AC018607.1 | 0.402372782 | 1.82E-17 | positive |
| MYL2 | AC010422.2 | 0.412089447 | 2.55E-18 | positive |
| CDKN2A | AC080129.2 | 0.454446583 | 2.18E-22 | positive |
| MLANA | AC002128.1 | 0.436360057 | 1.40E-20 | positive |
| RASA2 | AC002128.1 | 0.413834821 | 1.78E-18 | positive |
| MLANA | AL354989.1 | 0.410107327 | 3.82E-18 | positive |
| RASA2 | AC096992.2 | 0.433771305 | 2.48E-20 | positive |
| MLANA | AC008937.2 | 0.451735972 | 4.13E-22 | positive |
| RASA2 | AC008937.2 | 0.446400893 | 1.43E-21 | positive |
| CTSK | AC110995.1 | 0.493232706 | 1.20E-26 | positive |
| CYBB | AC110995.1 | 0.434733984 | 2.01E-20 | positive |
| VIM | AC110995.1 | 0.482235641 | 2.21E-25 | positive |
| CDC20 | AL163051.1 | 0.408378721 | 5.44E-18 | positive |
| MLANA | AC087501.1 | 0.527752286 | 6.39E-31 | positive |
| RASA2 | AC087501.1 | 0.455795852 | 1.58E-22 | positive |
| MLANA | AC007533.1 | 0.479189444 | 4.85E-25 | positive |
| RASA2 | AC007533.1 | 0.429525419 | 6.32E-20 | positive |
| CDKN2A | AC092171.5 | 0.407246614 | 6.84E-18 | positive |
| RAC1 | AC092171.5 | 0.421617938 | 3.47E-19 | positive |
| AR | AP001528.2 | 0.628206021 | 1.28E-46 | positive |
| KIF2C | AP001528.2 | -0.458166337 | 8.98E-23 | negative |
| MAD2L1 | AP001528.2 | -0.427107608 | 1.07E-19 | negative |
| MYLK | AP001528.2 | 0.502245293 | 1.03E-27 | positive |
| PCDH7 | AP001528.2 | 0.422302459 | 3.00E-19 | positive |
| PRND | AP001528.2 | 0.417082051 | 9.03E-19 | positive |
| PRNP | AP001528.2 | 0.455302804 | 1.78E-22 | positive |
| RNF146 | AP001528.2 | 0.407209971 | 6.90E-18 | positive |
| TUBA1A | AP001528.2 | 0.507757658 | 2.19E-28 | positive |
| VCL | AP001528.2 | 0.46458771 | 1.89E-23 | positive |
| VIM | AP001528.2 | 0.432273074 | 3.46E-20 | positive |
| MLANA | AC023509.6 | 0.431052228 | 4.52E-20 | positive |
| RASA2 | LINC00894 | 0.435805259 | 1.58E-20 | positive |
| AURKA | AC130456.8 | 0.56622248 | 2.66E-36 | positive |
| MCOLN1 | AC130456.8 | 0.496929805 | 4.43E-27 | positive |
| PIKFYVE | AP000866.6 | 0.42012506 | 4.76E-19 | positive |
| RASA2 | AP000866.6 | 0.418516413 | 6.68E-19 | positive |
| AR | AP001363.2 | 0.538446116 | 2.39E-32 | positive |
| CAV1 | AP001363.2 | 0.406592973 | 7.81E-18 | positive |
| MAP1LC3A | AP001363.2 | 0.40575049 | 9.26E-18 | positive |
| MAPT | AP001363.2 | 0.464950273 | 1.73E-23 | positive |
| MYLK | AP001363.2 | 0.691681044 | 6.38E-60 | positive |
| PCDH7 | AP001363.2 | 0.525245697 | 1.36E-30 | positive |
| PRKAA2 | AP001363.2 | 0.514744133 | 2.98E-29 | positive |
| PRNP | AP001363.2 | 0.581858756 | 1.07E-38 | positive |
| SRF | AP001363.2 | 0.526865012 | 8.36E-31 | positive |
| TUBA1A | AP001363.2 | 0.643698319 | 1.42E-49 | positive |
| VCL | AP001363.2 | 0.621224777 | 2.43E-45 | positive |
| RASA2 | AC091185.1 | 0.438433717 | 8.78E-21 | positive |
| CYBB | AC015911.3 | 0.521348273 | 4.33E-30 | positive |
| MLANA | AC099811.5 | 0.407810851 | 6.10E-18 | positive |
| CALB2 | LINC01711 | 0.642059365 | 2.97E-49 | positive |
| RASA2 | OPA1-AS1 | 0.401281284 | 2.26E-17 | positive |
| CALB2 | AC015660.2 | 0.502397948 | 9.84E-28 | positive |
| AR | GSN-AS1 | 0.557677307 | 4.80E-35 | positive |
| CDC20 | GSN-AS1 | -0.428259646 | 8.32E-20 | negative |
| KIF2C | GSN-AS1 | -0.471090813 | 3.78E-24 | negative |
| MAD2L1 | GSN-AS1 | -0.443390161 | 2.86E-21 | negative |
| MLANA | GSN-AS1 | 0.472762446 | 2.49E-24 | positive |
| MYLK | GSN-AS1 | 0.544224339 | 3.86E-33 | positive |
| PCDH7 | GSN-AS1 | 0.444903418 | 2.02E-21 | positive |
| PRKAA2 | GSN-AS1 | 0.433995464 | 2.36E-20 | positive |
| PRNP | GSN-AS1 | 0.413500841 | 1.90E-18 | positive |
| RNF146 | GSN-AS1 | 0.440798076 | 5.15E-21 | positive |
| ROCK1 | GSN-AS1 | 0.40363132 | 1.42E-17 | positive |
| SRF | GSN-AS1 | 0.444927367 | 2.01E-21 | positive |
| TUBA1A | GSN-AS1 | 0.482802129 | 1.90E-25 | positive |
| VCL | GSN-AS1 | 0.538733832 | 2.19E-32 | positive |
| MLANA | AC114730.3 | 0.420421725 | 4.47E-19 | positive |
| RASA2 | AC008870.2 | 0.547640186 | 1.29E-33 | positive |
| CUEDC2 | ZNF561-AS1 | 0.435944307 | 1.53E-20 | positive |
| DIAPH1 | ZNF561-AS1 | -0.435224888 | 1.80E-20 | negative |
| MLANA | AC017116.2 | 0.435766706 | 1.60E-20 | positive |
| MLANA | AL139407.1 | 0.437193781 | 1.16E-20 | positive |
| PIKFYVE | AL139407.1 | 0.449296579 | 7.31E-22 | positive |
| RASA2 | AL139407.1 | 0.547922762 | 1.18E-33 | positive |
| AR | AL356489.2 | 0.561060803 | 1.54E-35 | positive |
| ITGB1 | AL356489.2 | 0.4477271 | 1.05E-21 | positive |
| MAPT | AL356489.2 | 0.639243332 | 1.04E-48 | positive |
| MTMR7 | AL356489.2 | 0.627064432 | 2.09E-46 | positive |
| MTUS2 | AL356489.2 | 0.698565487 | 1.42E-61 | positive |
| MYLK | AL356489.2 | 0.724233472 | 3.53E-68 | positive |
| PCDH7 | AL356489.2 | 0.451586059 | 4.28E-22 | positive |
| PRKAA2 | AL356489.2 | 0.654776694 | 8.51E-52 | positive |
| PRNP | AL356489.2 | 0.596733183 | 4.23E-41 | positive |
| SRF | AL356489.2 | 0.547273462 | 1.45E-33 | positive |
| STMN2 | AL356489.2 | 0.467911958 | 8.35E-24 | positive |
| TUBA1A | AL356489.2 | 0.675679089 | 2.97E-56 | positive |
| VCL | AL356489.2 | 0.606493703 | 9.57E-43 | positive |
| IL6 | HIF1A-AS2 | 0.411179058 | 3.07E-18 | positive |
| PDPN | HIF1A-AS2 | 0.484637581 | 1.18E-25 | positive |
| RASA2 | MORF4L2-AS1 | 0.433036931 | 2.92E-20 | positive |
| VIM | AC138207.5 | 0.482478352 | 2.07E-25 | positive |
| TP63 | AL360091.1 | 0.401624227 | 2.11E-17 | positive |
| MLANA | AC025287.3 | 0.409047918 | 4.75E-18 | positive |
| PIKFYVE | AC025287.3 | 0.425192388 | 1.62E-19 | positive |
| RASA2 | AC025287.3 | 0.462829811 | 2.91E-23 | positive |
| CTSK | AL139393.2 | 0.540038001 | 1.45E-32 | positive |
| PDPN | AL139393.2 | 0.435805103 | 1.58E-20 | positive |
| TGFB1 | AL139393.2 | 0.451988713 | 3.89E-22 | positive |
| VIM | AL139393.2 | 0.467510443 | 9.22E-24 | positive |
| SPRN | LINC01521 | 0.404840102 | 1.11E-17 | positive |
| KIF2C | SNHG1 | 0.407822175 | 6.09E-18 | positive |
| MAD2L1 | SNHG1 | 0.512986024 | 4.95E-29 | positive |
| TUBA1A | LINC01082 | 0.419450154 | 5.49E-19 | positive |
| MLANA | NARF-IT1 | 0.423863436 | 2.15E-19 | positive |
| RASA2 | NARF-IT1 | 0.46600128 | 1.34E-23 | positive |
| RASA2 | AC021078.1 | 0.562180458 | 1.06E-35 | positive |
| MLANA | AC084824.3 | 0.556655412 | 6.74E-35 | positive |
| RASA2 | AC084824.3 | 0.493118467 | 1.24E-26 | positive |
| RNF146 | AC084824.3 | 0.403143184 | 1.56E-17 | positive |
| ROCK1 | AC084824.3 | 0.400719191 | 2.53E-17 | positive |
| MLANA | AC020908.3 | 0.43962609 | 6.72E-21 | positive |
| PDPN | AC114488.2 | 0.408556785 | 5.25E-18 | positive |
| RASA2 | FMR1-IT1 | 0.417402082 | 8.44E-19 | positive |
| AR | ZNF667-AS1 | 0.596863339 | 4.03E-41 | positive |
| MAPT | ZNF667-AS1 | 0.513418876 | 4.37E-29 | positive |
| MYLK | ZNF667-AS1 | 0.636402355 | 3.67E-48 | positive |
| PCDH7 | ZNF667-AS1 | 0.421161979 | 3.82E-19 | positive |
| PRKAA2 | ZNF667-AS1 | 0.539695474 | 1.62E-32 | positive |
| PRNP | ZNF667-AS1 | 0.590152596 | 5.07E-40 | positive |
| RNF146 | ZNF667-AS1 | 0.406129673 | 8.58E-18 | positive |
| SRF | ZNF667-AS1 | 0.443911588 | 2.54E-21 | positive |
| TUBA1A | ZNF667-AS1 | 0.635378379 | 5.76E-48 | positive |
| VCL | ZNF667-AS1 | 0.534442657 | 8.30E-32 | positive |
| CTSK | LINC01094 | 0.578045514 | 4.22E-38 | positive |
| CTSL | LINC01094 | 0.615136185 | 2.99E-44 | positive |
| CTSS | LINC01094 | 0.430767959 | 4.81E-20 | positive |
| CYBB | LINC01094 | 0.640583803 | 5.75E-49 | positive |
| PDPN | LINC01094 | 0.402109678 | 1.92E-17 | positive |
| VIM | LINC01094 | 0.434580982 | 2.08E-20 | positive |
| NTS | AC106900.2 | 0.441705964 | 4.19E-21 | positive |
| MLANA | AC022558.1 | 0.422518047 | 2.86E-19 | positive |
| MLANA | AC084018.1 | 0.494118466 | 9.49E-27 | positive |
| PIKFYVE | AC084018.1 | 0.439305651 | 7.22E-21 | positive |
| RASA2 | AC084018.1 | 0.428922154 | 7.21E-20 | positive |
| AR | FGF14-AS2 | 0.5741824 | 1.66E-37 | positive |
| DIAPH1 | FGF14-AS2 | -0.412097506 | 2.54E-18 | negative |
| MAPT | FGF14-AS2 | 0.550203269 | 5.63E-34 | positive |
| MYLK | FGF14-AS2 | 0.667866783 | 1.52E-54 | positive |
| PCDH7 | FGF14-AS2 | 0.415921877 | 1.15E-18 | positive |
| PRKAA2 | FGF14-AS2 | 0.585924928 | 2.42E-39 | positive |
| PRNP | FGF14-AS2 | 0.587510945 | 1.35E-39 | positive |
| SRF | FGF14-AS2 | 0.514687151 | 3.03E-29 | positive |
| STMN2 | FGF14-AS2 | 0.443737725 | 2.64E-21 | positive |
| TUBA1A | FGF14-AS2 | 0.69331554 | 2.61E-60 | positive |
| VCL | FGF14-AS2 | 0.508427497 | 1.82E-28 | positive |
| MLANA | AC025430.1 | 0.403252354 | 1.53E-17 | positive |
| TF | AL121832.1 | 0.408569725 | 5.23E-18 | positive |
| AR | AC004825.2 | 0.42366663 | 2.24E-19 | positive |
| MYLK | AC004825.2 | 0.454280792 | 2.27E-22 | positive |
| PRKAA2 | AC004825.2 | 0.430134506 | 5.53E-20 | positive |
| PRNP | AC004825.2 | 0.48742028 | 5.68E-26 | positive |
| TUBA1A | AC004825.2 | 0.464870201 | 1.77E-23 | positive |
| AR | AC027807.2 | 0.569849736 | 7.59E-37 | positive |
| ARHGAP36 | AC027807.2 | 0.424173705 | 2.01E-19 | positive |
| MAPT | AC027807.2 | 0.45060436 | 5.39E-22 | positive |
| MYLK | AC027807.2 | 0.57737015 | 5.37E-38 | positive |
| PCDH7 | AC027807.2 | 0.455079833 | 1.88E-22 | positive |
| PRKAA2 | AC027807.2 | 0.449647813 | 6.74E-22 | positive |
| PRNP | AC027807.2 | 0.505498152 | 4.14E-28 | positive |
| TUBA1A | AC027807.2 | 0.557963641 | 4.36E-35 | positive |
| VCL | AC027807.2 | 0.470402369 | 4.49E-24 | positive |
| AR | PAXIP1-AS2 | 0.403936811 | 1.33E-17 | positive |
| CDC20 | PAXIP1-AS2 | -0.438202375 | 9.25E-21 | negative |
| MYLK | PAXIP1-AS2 | 0.400704242 | 2.54E-17 | positive |
| RNF146 | AL109955.1 | 0.436270823 | 1.43E-20 | positive |
| ARHGAP36 | AC017104.3 | 0.4376987 | 1.04E-20 | positive |
| MYL2 | AC017104.3 | 0.479568872 | 4.40E-25 | positive |
| MLANA | AC048341.1 | 0.444858351 | 2.04E-21 | positive |
| RASA2 | AC048341.1 | 0.434133542 | 2.29E-20 | positive |
| RNF146 | AC048341.1 | 0.436717561 | 1.29E-20 | positive |
| RASA2 | AC069257.1 | 0.436040999 | 1.50E-20 | positive |
| AR | SENCR | 0.456034804 | 1.50E-22 | positive |
| CDH1 | SENCR | -0.46426447 | 2.05E-23 | negative |
| CTSK | SENCR | 0.46648836 | 1.19E-23 | positive |
| S100B | SENCR | 0.433589082 | 2.59E-20 | positive |
| TGFB1 | SENCR | 0.583954804 | 4.99E-39 | positive |
| TUBA1A | SENCR | 0.469440196 | 5.71E-24 | positive |
| VIM | SENCR | 0.657796138 | 2.03E-52 | positive |
| AR | LOH12CR2 | 0.445851532 | 1.62E-21 | positive |
| DIAPH1 | LOH12CR2 | -0.437791038 | 1.01E-20 | negative |
| MAPT | LOH12CR2 | 0.511605179 | 7.35E-29 | positive |
| MYLK | LOH12CR2 | 0.534259638 | 8.78E-32 | positive |
| PRKAA2 | LOH12CR2 | 0.497479932 | 3.81E-27 | positive |
| PRNP | LOH12CR2 | 0.469517406 | 5.60E-24 | positive |
| TUBA1A | LOH12CR2 | 0.529910164 | 3.33E-31 | positive |
| ARHGAP36 | AC092422.1 | 0.428890696 | 7.25E-20 | positive |
| MLANA | AL450998.2 | 0.515195669 | 2.62E-29 | positive |
| PIKFYVE | AL450998.2 | 0.432433277 | 3.34E-20 | positive |
| RASA2 | AL450998.2 | 0.526980488 | 8.07E-31 | positive |
| RASA2 | AL031600.1 | 0.430836697 | 4.74E-20 | positive |
| TF | AL049612.1 | 0.400028276 | 2.90E-17 | positive |
| RASA2 | AC245060.2 | 0.429710008 | 6.07E-20 | positive |
| AR | AL049838.1 | 0.56324737 | 7.35E-36 | positive |
| KIF2C | AL049838.1 | -0.405786138 | 9.19E-18 | negative |
| MYLK | AL049838.1 | 0.546242446 | 2.02E-33 | positive |
| PCDH7 | AL049838.1 | 0.427240083 | 1.04E-19 | positive |
| PRKAA2 | AL049838.1 | 0.458675776 | 7.95E-23 | positive |
| PRNP | AL049838.1 | 0.520287027 | 5.92E-30 | positive |
| RNF146 | AL049838.1 | 0.440881182 | 5.06E-21 | positive |
| TUBA1A | AL049838.1 | 0.636651675 | 3.29E-48 | positive |
| VCL | AL049838.1 | 0.473041198 | 2.32E-24 | positive |
| VIM | AL049838.1 | 0.604899114 | 1.79E-42 | positive |
| WT1 | AL049838.1 | 0.544322578 | 3.74E-33 | positive |
| CTSL | SMIM25 | 0.523287474 | 2.44E-30 | positive |
| CYBB | SMIM25 | 0.459119599 | 7.14E-23 | positive |
| CTSK | AL356417.2 | 0.56812651 | 1.38E-36 | positive |
| PDPN | AL356417.2 | 0.47355591 | 2.04E-24 | positive |
| PRND | AL356417.2 | 0.452145433 | 3.75E-22 | positive |
| TGFB1 | AL356417.2 | 0.419370292 | 5.58E-19 | positive |
| VIM | AL356417.2 | 0.568778182 | 1.10E-36 | positive |
| ROCK1 | AC127024.3 | 0.419176713 | 5.82E-19 | positive |
| AR | AC080038.1 | 0.500347193 | 1.73E-27 | positive |
| CTSK | AC080038.1 | 0.500800793 | 1.53E-27 | positive |
| MAD2L1 | AC080038.1 | -0.401704645 | 2.08E-17 | negative |
| MRTFA | AC080038.1 | 0.42144224 | 3.60E-19 | positive |
| PDPN | AC080038.1 | 0.417118778 | 8.96E-19 | positive |
| PRND | AC080038.1 | 0.437550299 | 1.07E-20 | positive |
| PRNP | AC080038.1 | 0.443730939 | 2.64E-21 | positive |
| TGFB1 | AC080038.1 | 0.614307659 | 4.19E-44 | positive |
| TUBA1A | AC080038.1 | 0.563570941 | 6.58E-36 | positive |
| VIM | AC080038.1 | 0.515567286 | 2.35E-29 | positive |
| MYLK | AC116667.1 | 0.417422603 | 8.41E-19 | positive |
| PRKAA2 | AC116667.1 | 0.41088417 | 3.26E-18 | positive |
| PRNP | AC116667.1 | 0.404950661 | 1.09E-17 | positive |
| RNF146 | AC116667.1 | 0.507801716 | 2.17E-28 | positive |
| TUBA1A | AC116667.1 | 0.432317297 | 3.43E-20 | positive |
| PRKAA2 | LINC01160 | 0.405869152 | 9.04E-18 | positive |
| MLANA | AC005519.1 | 0.442535672 | 3.47E-21 | positive |
| RASA2 | AC005519.1 | 0.485834501 | 8.62E-26 | positive |
| MLANA | Z83843.1 | 0.480536508 | 3.43E-25 | positive |
| RASA2 | Z83843.1 | 0.553934439 | 1.66E-34 | positive |
| RNF146 | Z83843.1 | 0.500800775 | 1.53E-27 | positive |
| ROCK1 | Z83843.1 | 0.450724213 | 5.24E-22 | positive |
| MLANA | AC073569.1 | 0.479963628 | 3.97E-25 | positive |
| RNF146 | AC073569.1 | 0.472028521 | 2.99E-24 | positive |
| ROCK1 | AC073569.1 | 0.497946658 | 3.35E-27 | positive |
| NTS | AC007566.1 | 0.699993258 | 6.35E-62 | positive |
| MLANA | AC020913.3 | 0.42555338 | 1.49E-19 | positive |
| AR | NR2F1-AS1 | 0.718974751 | 9.13E-67 | positive |
| CAV1 | NR2F1-AS1 | 0.44709818 | 1.22E-21 | positive |
| CDC20 | NR2F1-AS1 | -0.400689216 | 2.55E-17 | negative |
| CDH1 | NR2F1-AS1 | -0.454942428 | 1.94E-22 | negative |
| ITGB1 | NR2F1-AS1 | 0.504166205 | 6.01E-28 | positive |
| KIF2C | NR2F1-AS1 | -0.472318298 | 2.78E-24 | negative |
| MAD2L1 | NR2F1-AS1 | -0.411722617 | 2.75E-18 | negative |
| MAPT | NR2F1-AS1 | 0.515585849 | 2.34E-29 | positive |
| MYLK | NR2F1-AS1 | 0.765179548 | 2.01E-80 | positive |
| PCDH7 | NR2F1-AS1 | 0.629304494 | 8.01E-47 | positive |
| PRKAA2 | NR2F1-AS1 | 0.574601877 | 1.44E-37 | positive |
| PRNP | NR2F1-AS1 | 0.707176779 | 1.04E-63 | positive |
| RNF146 | NR2F1-AS1 | 0.49792724 | 3.37E-27 | positive |
| SRF | NR2F1-AS1 | 0.532465471 | 1.52E-31 | positive |
| STMN2 | NR2F1-AS1 | 0.414913799 | 1.42E-18 | positive |
| TUBA1A | NR2F1-AS1 | 0.746006217 | 2.14E-74 | positive |
| VCL | NR2F1-AS1 | 0.705288986 | 3.10E-63 | positive |
| VIM | NR2F1-AS1 | 0.518998036 | 8.64E-30 | positive |
| LPAR2 | LINC01978 | 0.408613482 | 5.19E-18 | positive |
| TM9SF4 | SNHG11 | 0.518249155 | 1.08E-29 | positive |
| MLANA | AC073517.1 | 0.402016729 | 1.96E-17 | positive |
| CUEDC2 | AL391121.1 | 0.448617936 | 8.56E-22 | positive |
| MAD2L1 | AL391121.1 | -0.452205455 | 3.70E-22 | negative |
| TGFB1 | AL391121.1 | 0.424990368 | 1.69E-19 | positive |
| RASA2 | AC018653.3 | 0.40369036 | 1.40E-17 | positive |
| NTS | HAGLROS | 0.618914931 | 6.34E-45 | positive |
| MLANA | AC023389.1 | 0.445533307 | 1.75E-21 | positive |
| RASA2 | AC108727.1 | 0.495915613 | 5.83E-27 | positive |
| RASA2 | AC090948.3 | 0.4965511 | 4.91E-27 | positive |
| PIKFYVE | AP002784.1 | 0.43190742 | 3.75E-20 | positive |
| RASA2 | AC132192.1 | 0.410266729 | 3.70E-18 | positive |
| RASA2 | AL354733.3 | 0.408548803 | 5.25E-18 | positive |
| PIKFYVE | AP001432.1 | 0.411019695 | 3.17E-18 | positive |
| RASA2 | AP001432.1 | 0.401255747 | 2.28E-17 | positive |
| CALB2 | MIR31HG | 0.509841539 | 1.22E-28 | positive |
| CDH3 | MIR31HG | 0.426086369 | 1.33E-19 | positive |
| KRT7 | MIR31HG | 0.46179089 | 3.75E-23 | positive |
| CYBB | AC008105.3 | 0.408645783 | 5.15E-18 | positive |
| MLANA | AC008105.3 | 0.440495795 | 5.52E-21 | positive |
| RASA2 | AC008105.3 | 0.479589118 | 4.37E-25 | positive |
| MLANA | AC011120.1 | 0.432271333 | 3.46E-20 | positive |
| MYL2 | AC103691.1 | 0.491443159 | 1.95E-26 | positive |
| AR | AC005180.1 | 0.612441334 | 8.91E-44 | positive |
| ITGB1 | AC005180.1 | 0.409716158 | 4.14E-18 | positive |
| MAPT | AC005180.1 | 0.674275075 | 6.08E-56 | positive |
| MYLK | AC005180.1 | 0.732637007 | 1.66E-70 | positive |
| PCDH7 | AC005180.1 | 0.601699928 | 6.26E-42 | positive |
| PRKAA2 | AC005180.1 | 0.527211251 | 7.53E-31 | positive |
| PRNP | AC005180.1 | 0.585690333 | 2.64E-39 | positive |
| SRF | AC005180.1 | 0.583387216 | 6.14E-39 | positive |
| TUBA1A | AC005180.1 | 0.605661105 | 1.33E-42 | positive |
| VCL | AC005180.1 | 0.652271096 | 2.76E-51 | positive |
| GZMB | LINC02195 | 0.748609686 | 3.50E-75 | positive |
| TNFSF10 | LINC02195 | 0.435122378 | 1.84E-20 | positive |
| AR | AC087477.2 | 0.553694327 | 1.80E-34 | positive |
| MYLK | AC087477.2 | 0.46450921 | 1.93E-23 | positive |
| PCDH7 | AC087477.2 | 0.40060935 | 2.59E-17 | positive |
| PRKAA2 | AC087477.2 | 0.421924335 | 3.25E-19 | positive |
| TUBA1A | AC087477.2 | 0.416573039 | 1.00E-18 | positive |
| VCL | AC087477.2 | 0.422523657 | 2.86E-19 | positive |
| AR | MBNL1-AS1 | 0.61910555 | 5.86E-45 | positive |
| CAV1 | MBNL1-AS1 | 0.494495894 | 8.57E-27 | positive |
| ITGB1 | MBNL1-AS1 | 0.650625487 | 5.93E-51 | positive |
| MAPT | MBNL1-AS1 | 0.61007188 | 2.31E-43 | positive |
| MYLK | MBNL1-AS1 | 0.926596373 | 3.04E-176 | positive |
| PCDH7 | MBNL1-AS1 | 0.594161579 | 1.12E-40 | positive |
| PRKAA2 | MBNL1-AS1 | 0.697371403 | 2.76E-61 | positive |
| PRNP | MBNL1-AS1 | 0.756442916 | 1.31E-77 | positive |
| RNF146 | MBNL1-AS1 | 0.419145662 | 5.85E-19 | positive |
| ROCK1 | MBNL1-AS1 | 0.437708118 | 1.03E-20 | positive |
| SRF | MBNL1-AS1 | 0.66880123 | 9.55E-55 | positive |
| TUBA1A | MBNL1-AS1 | 0.791640892 | 9.31E-90 | positive |
| VCL | MBNL1-AS1 | 0.811828669 | 7.63E-98 | positive |
| NTS | AC004908.1 | 0.43372891 | 2.51E-20 | positive |
| MLANA | AL354892.3 | 0.415996686 | 1.13E-18 | positive |
| STMN2 | AC006504.5 | 0.401234654 | 2.29E-17 | positive |
| CDKN2A | KCNMB2-AS1 | 0.402225345 | 1.88E-17 | positive |
| AR | AL357054.4 | 0.682942669 | 6.87E-58 | positive |
| CAV1 | AL357054.4 | 0.412160014 | 2.51E-18 | positive |
| CDH1 | AL357054.4 | -0.412653357 | 2.27E-18 | negative |
| ITGB1 | AL357054.4 | 0.493871964 | 1.01E-26 | positive |
| MAPT | AL357054.4 | 0.546151932 | 2.08E-33 | positive |
| MYLK | AL357054.4 | 0.829324885 | 1.10E-105 | positive |
| PCDH7 | AL357054.4 | 0.589654384 | 6.10E-40 | positive |
| PRKAA2 | AL357054.4 | 0.63620082 | 4.01E-48 | positive |
| PRNP | AL357054.4 | 0.693099149 | 2.94E-60 | positive |
| RNF146 | AL357054.4 | 0.428198599 | 8.43E-20 | positive |
| SRF | AL357054.4 | 0.584212163 | 4.54E-39 | positive |
| TUBA1A | AL357054.4 | 0.734318008 | 5.55E-71 | positive |
| VCL | AL357054.4 | 0.708138522 | 5.92E-64 | positive |
| NTS | LINC00239 | 0.681141969 | 1.77E-57 | positive |
| MLANA | AC016405.1 | 0.466351495 | 1.23E-23 | positive |
| RASA2 | AC016405.1 | 0.403625777 | 1.42E-17 | positive |
| RNF146 | AF117829.1 | 0.417597288 | 8.11E-19 | positive |
| MLANA | GARS-DT | 0.468194942 | 7.78E-24 | positive |
| RASA2 | GARS-DT | 0.444050684 | 2.46E-21 | positive |
| TM9SF4 | ZNF337-AS1 | 0.421175944 | 3.81E-19 | positive |
| NTS | HOXC-AS3 | 0.431018601 | 4.56E-20 | positive |
| TM9SF4 | RALY-AS1 | 0.537453482 | 3.26E-32 | positive |
| MYLK | AL137003.1 | 0.401157117 | 2.32E-17 | positive |
| PRNP | AL137003.1 | 0.42180546 | 3.33E-19 | positive |
| RASA2 | AL117209.1 | 0.405790337 | 9.19E-18 | positive |
| RASA2 | AC004148.2 | 0.486319541 | 7.59E-26 | positive |
| MYL2 | AC005529.1 | 0.463495692 | 2.47E-23 | positive |
| MLANA | AC007878.1 | 0.489597283 | 3.19E-26 | positive |
| RASA2 | AC007878.1 | 0.479645733 | 4.31E-25 | positive |
| ROCK1 | AC007878.1 | 0.412934549 | 2.14E-18 | positive |
| MLANA | AC027796.5 | 0.436883323 | 1.24E-20 | positive |
| PIK3C3 | AC012254.1 | 0.400819133 | 2.48E-17 | positive |
| RASA2 | INE1 | 0.490484009 | 2.52E-26 | positive |
| AR | AP001189.3 | 0.443211527 | 2.98E-21 | positive |
| CTSK | AP001189.3 | 0.508854026 | 1.61E-28 | positive |
| KIF2C | AP001189.3 | -0.407245983 | 6.85E-18 | negative |
| MAD2L1 | AP001189.3 | -0.452750378 | 3.26E-22 | negative |
| PRND | AP001189.3 | 0.432105427 | 3.59E-20 | positive |
| TGFB1 | AP001189.3 | 0.490690891 | 2.38E-26 | positive |
| RASA2 | AL022328.3 | 0.484518438 | 1.22E-25 | positive |
| MTMR7 | AL137798.1 | 0.532470974 | 1.52E-31 | positive |
| MLANA | AC020612.3 | 0.443203392 | 2.98E-21 | positive |
| AR | LINC01579 | 0.555591501 | 9.60E-35 | positive |
| ARHGAP36 | LINC01579 | 0.40284445 | 1.66E-17 | positive |
| MAPT | LINC01579 | 0.426345522 | 1.26E-19 | positive |
| MYLK | LINC01579 | 0.683467686 | 5.21E-58 | positive |
| PCDH7 | LINC01579 | 0.438021608 | 9.64E-21 | positive |
| PRKAA2 | LINC01579 | 0.578707683 | 3.33E-38 | positive |
| PRNP | LINC01579 | 0.622414953 | 1.48E-45 | positive |
| SRF | LINC01579 | 0.504493813 | 5.49E-28 | positive |
| STMN2 | LINC01579 | 0.413581832 | 1.87E-18 | positive |
| TUBA1A | LINC01579 | 0.630067565 | 5.78E-47 | positive |
| VCL | LINC01579 | 0.535757956 | 5.53E-32 | positive |
| MTMR7 | AC026333.4 | 0.404366769 | 1.22E-17 | positive |
| AR | AC120498.10 | 0.433452657 | 2.67E-20 | positive |
| MYLK | AC120498.10 | 0.520306975 | 5.88E-30 | positive |
| PRKAA2 | AC120498.10 | 0.423486776 | 2.33E-19 | positive |
| PRNP | AC120498.10 | 0.407010942 | 7.18E-18 | positive |
| TUBA1A | AC120498.10 | 0.50186452 | 1.14E-27 | positive |
| VCL | AC120498.10 | 0.427524331 | 9.76E-20 | positive |
| CXCL8 | LUCAT1 | 0.497422911 | 3.87E-27 | positive |
| ROCK1 | AC133550.2 | 0.419400254 | 5.55E-19 | positive |
| CTSK | AC009093.1 | 0.400407683 | 2.69E-17 | positive |
| RAC1 | AL049835.1 | 0.403601897 | 1.43E-17 | positive |
| PDPN | AL137186.2 | 0.477864042 | 6.81E-25 | positive |
| AR | AC004637.1 | 0.672138985 | 1.79E-55 | positive |
| CAV1 | AC004637.1 | 0.464224374 | 2.07E-23 | positive |
| ITGB1 | AC004637.1 | 0.434518297 | 2.11E-20 | positive |
| MAPT | AC004637.1 | 0.610899964 | 1.66E-43 | positive |
| MYLK | AC004637.1 | 0.798665601 | 1.82E-92 | positive |
| PCDH7 | AC004637.1 | 0.540826846 | 1.13E-32 | positive |
| PRKAA2 | AC004637.1 | 0.57786009 | 4.51E-38 | positive |
| PRNP | AC004637.1 | 0.688527121 | 3.52E-59 | positive |
| SRF | AC004637.1 | 0.646797964 | 3.46E-50 | positive |
| TUBA1A | AC004637.1 | 0.716429838 | 4.29E-66 | positive |
| VCL | AC004637.1 | 0.703523503 | 8.54E-63 | positive |
| RASA2 | LINC01355 | 0.406792949 | 7.50E-18 | positive |
| MLANA | AC012615.6 | 0.474585009 | 1.57E-24 | positive |
| RASA2 | AC012615.6 | 0.503699095 | 6.85E-28 | positive |
| MLANA | FP325332.1 | 0.441662453 | 4.23E-21 | positive |
| MLANA | AC005086.1 | 0.467718622 | 8.76E-24 | positive |
| ROCK1 | AC005086.1 | 0.425146121 | 1.63E-19 | positive |
| AURKA | AC007950.1 | 0.570185619 | 6.76E-37 | positive |
| MCOLN1 | AC007950.1 | 0.489676708 | 3.12E-26 | positive |
| CYBB | AL135818.1 | 0.536985719 | 3.78E-32 | positive |
| AR | AC015922.2 | 0.463958373 | 2.21E-23 | positive |
| PRKAA2 | AC015922.2 | 0.40498788 | 1.08E-17 | positive |
| RASA2 | AL355075.2 | 0.452177049 | 3.73E-22 | positive |
| CDKN2A | AC243964.2 | 0.44857839 | 8.64E-22 | positive |
| ATG5 | LINC02526 | 0.477022341 | 8.45E-25 | positive |
| KIF2C | SNHG3 | 0.417128378 | 8.94E-19 | positive |
| MAD2L1 | SNHG3 | 0.412848578 | 2.18E-18 | positive |
| MLANA | AC002558.3 | 0.402381928 | 1.82E-17 | positive |
| ROCK1 | AC002558.3 | 0.47397872 | 1.83E-24 | positive |
| MYL2 | AC068189.1 | 0.419609827 | 5.31E-19 | positive |
| AR | BARX1-DT | 0.691875296 | 5.74E-60 | positive |
| CAV1 | BARX1-DT | 0.41286221 | 2.17E-18 | positive |
| ITGB1 | BARX1-DT | 0.471574297 | 3.35E-24 | positive |
| MAPT | BARX1-DT | 0.562254424 | 1.03E-35 | positive |
| MYLK | BARX1-DT | 0.842619031 | 2.88E-112 | positive |
| PCDH7 | BARX1-DT | 0.637655697 | 2.11E-48 | positive |
| PRKAA2 | BARX1-DT | 0.680502011 | 2.47E-57 | positive |
| PRNP | BARX1-DT | 0.709983221 | 2.01E-64 | positive |
| ROCK1 | BARX1-DT | 0.402295089 | 1.85E-17 | positive |
| SRF | BARX1-DT | 0.650039847 | 7.78E-51 | positive |
| TUBA1A | BARX1-DT | 0.689495067 | 2.09E-59 | positive |
| VCL | BARX1-DT | 0.744006646 | 8.45E-74 | positive |
| MLANA | AC211433.1 | 0.467343518 | 9.61E-24 | positive |
| RASA2 | AC211433.1 | 0.414256473 | 1.63E-18 | positive |
| ROCK1 | AC004477.3 | 0.434728474 | 2.01E-20 | positive |
| AR | DNAH10OS | 0.445303915 | 1.84E-21 | positive |
| MYLK | DNAH10OS | 0.507503422 | 2.36E-28 | positive |
| PRKAA2 | DNAH10OS | 0.429589328 | 6.23E-20 | positive |
| PRNP | DNAH10OS | 0.405259528 | 1.02E-17 | positive |
| SRF | DNAH10OS | 0.41807845 | 7.33E-19 | positive |
| TUBA1A | DNAH10OS | 0.445402724 | 1.80E-21 | positive |
| VCL | DNAH10OS | 0.478691815 | 5.51E-25 | positive |
| MLANA | AC104984.6 | 0.411611078 | 2.81E-18 | positive |
| MLANA | GUSBP11 | 0.413657576 | 1.84E-18 | positive |
| RASA2 | GUSBP11 | 0.41653449 | 1.01E-18 | positive |
| RHOA | GUSBP11 | -0.411436765 | 2.91E-18 | negative |
| MLANA | AC011939.2 | 0.454331472 | 2.24E-22 | positive |
| MAPT | AC007541.1 | 0.549173631 | 7.86E-34 | positive |
| MTUS2 | AC007541.1 | 0.411593681 | 2.82E-18 | positive |
| MYLK | AC007541.1 | 0.431581698 | 4.03E-20 | positive |
| PRKAA2 | AC007541.1 | 0.454720483 | 2.04E-22 | positive |
| PRNP | AC007541.1 | 0.479353354 | 4.65E-25 | positive |
| TUBA1A | AC007541.1 | 0.491859503 | 1.74E-26 | positive |
| AR | PART1 | 0.629277821 | 8.11E-47 | positive |
| CAV1 | PART1 | 0.441325814 | 4.57E-21 | positive |
| ITGB1 | PART1 | 0.50812964 | 1.98E-28 | positive |
| MAPT | PART1 | 0.554011602 | 1.62E-34 | positive |
| MYLK | PART1 | 0.886467495 | 2.85E-139 | positive |
| PCDH7 | PART1 | 0.616128329 | 1.99E-44 | positive |
| PRKAA2 | PART1 | 0.705041463 | 3.57E-63 | positive |
| PRNP | PART1 | 0.71944489 | 6.85E-67 | positive |
| SRF | PART1 | 0.682332331 | 9.47E-58 | positive |
| TUBA1A | PART1 | 0.781170037 | 6.58E-86 | positive |
| VCL | PART1 | 0.727340584 | 4.98E-69 | positive |
| ROCK1 | AC016949.1 | 0.427442133 | 9.94E-20 | positive |
| MLANA | AC254562.3 | 0.409448227 | 4.37E-18 | positive |
| SPRN | AC254562.3 | 0.402967087 | 1.62E-17 | positive |
| MLANA | SAP30L-AS1 | 0.44331763 | 2.90E-21 | positive |
| RASA2 | SAP30L-AS1 | 0.403641187 | 1.41E-17 | positive |
| AR | AC053503.4 | 0.65217089 | 2.89E-51 | positive |
| CAV1 | AC053503.4 | 0.489466426 | 3.30E-26 | positive |
| ITGB1 | AC053503.4 | 0.572048688 | 3.52E-37 | positive |
| MAPT | AC053503.4 | 0.635602321 | 5.22E-48 | positive |
| MYLK | AC053503.4 | 0.885823052 | 8.49E-139 | positive |
| PCDH7 | AC053503.4 | 0.547242183 | 1.47E-33 | positive |
| PRKAA2 | AC053503.4 | 0.674932883 | 4.35E-56 | positive |
| PRNP | AC053503.4 | 0.742889954 | 1.81E-73 | positive |
| RNF146 | AC053503.4 | 0.418898486 | 6.17E-19 | positive |
| ROCK1 | AC053503.4 | 0.408305428 | 5.52E-18 | positive |
| S100B | AC053503.4 | 0.423117188 | 2.52E-19 | positive |
| SRF | AC053503.4 | 0.633481704 | 1.32E-47 | positive |
| TUBA1A | AC053503.4 | 0.811740659 | 8.32E-98 | positive |
| VCL | AC053503.4 | 0.778588748 | 5.43E-85 | positive |
| PDPN | AC114284.1 | 0.591162643 | 3.48E-40 | positive |
| MLANA | AC011465.1 | 0.435557751 | 1.67E-20 | positive |
| RASA2 | AC011465.1 | 0.400809252 | 2.49E-17 | positive |
| RASA2 | N4BP2L2-IT2 | 0.476337962 | 1.01E-24 | positive |
| CDKN2A | AC138150.2 | 0.426284054 | 1.28E-19 | positive |
| MLANA | AC015849.3 | 0.447827513 | 1.03E-21 | positive |
| RASA2 | AC015849.3 | 0.429760055 | 6.00E-20 | positive |
| MLANA | AC009120.2 | 0.464322418 | 2.02E-23 | positive |
| RASA2 | AC009120.2 | 0.532725801 | 1.41E-31 | positive |
| RNF146 | AC009120.2 | 0.406644707 | 7.73E-18 | positive |
| IL6 | AC018682.1 | 0.60194839 | 5.68E-42 | positive |
| PDPN | AC018682.1 | 0.575330227 | 1.11E-37 | positive |
| RNF146 | AP001107.4 | 0.401931141 | 1.99E-17 | positive |
| CTSK | LINC01614 | 0.445280353 | 1.85E-21 | positive |
| CTSL | LINC01614 | 0.456888536 | 1.22E-22 | positive |
| MLANA | AP001442.1 | 0.425833107 | 1.41E-19 | positive |
| MLANA | AL136980.1 | 0.459321012 | 6.80E-23 | positive |
| AR | LINC00921 | 0.418932187 | 6.12E-19 | positive |
| CDC20 | LINC00921 | -0.418758231 | 6.35E-19 | negative |
| KIF2C | LINC00921 | -0.440470116 | 5.55E-21 | negative |
| MAD2L1 | LINC00921 | -0.427886124 | 9.03E-20 | negative |
| MLANA | LINC00921 | 0.440991989 | 4.93E-21 | positive |
| RNF146 | LINC00921 | 0.429546342 | 6.29E-20 | positive |
| TUBA1A | LINC00921 | 0.400178059 | 2.82E-17 | positive |
| MYL2 | AC018761.1 | 0.494949757 | 7.58E-27 | positive |
| MLANA | AL021707.8 | 0.441833713 | 4.07E-21 | positive |
| RASA2 | AL021707.8 | 0.435683648 | 1.63E-20 | positive |
| CTSB | AC105001.1 | 0.558306465 | 3.89E-35 | positive |
| CXCL8 | GK-IT1 | 0.408204314 | 5.64E-18 | positive |
| IL6 | GK-IT1 | 0.455371183 | 1.75E-22 | positive |
| PDPN | GK-IT1 | 0.508677082 | 1.69E-28 | positive |
| MYL2 | AC008915.1 | 0.430733979 | 4.85E-20 | positive |
| MLANA | AL049869.3 | 0.511680206 | 7.20E-29 | positive |
| RASA2 | AL049869.3 | 0.482288971 | 2.18E-25 | positive |
| UVRAG | AP002340.1 | 0.438599359 | 8.46E-21 | positive |
| MLANA | AC011825.2 | 0.414995113 | 1.40E-18 | positive |
| RASA2 | AC011825.2 | 0.410565521 | 3.48E-18 | positive |
| MLANA | AC020978.3 | 0.446413081 | 1.43E-21 | positive |
| RASA2 | AC020978.3 | 0.463703688 | 2.35E-23 | positive |
| MTMR7 | AL121721.1 | 0.485356923 | 9.77E-26 | positive |
| MYL2 | AC002306.1 | 0.436494547 | 1.36E-20 | positive |
| MLANA | TRMT2B-AS1 | 0.43204413 | 3.64E-20 | positive |
| AR | AL928921.2 | 0.701763946 | 2.33E-62 | positive |
| CDH1 | AL928921.2 | -0.475208651 | 1.34E-24 | negative |
| ITGB1 | AL928921.2 | 0.442751234 | 3.31E-21 | positive |
| KIF2C | AL928921.2 | -0.407506178 | 6.49E-18 | negative |
| MAD2L1 | AL928921.2 | -0.459051589 | 7.26E-23 | negative |
| MAPT | AL928921.2 | 0.519538088 | 7.38E-30 | positive |
| MYLK | AL928921.2 | 0.710289396 | 1.68E-64 | positive |
| PCDH7 | AL928921.2 | 0.559971073 | 2.23E-35 | positive |
| PRKAA2 | AL928921.2 | 0.525794804 | 1.15E-30 | positive |
| PRND | AL928921.2 | 0.404027596 | 1.31E-17 | positive |
| PRNP | AL928921.2 | 0.632926311 | 1.68E-47 | positive |
| RNF146 | AL928921.2 | 0.452283181 | 3.63E-22 | positive |
| S100B | AL928921.2 | 0.415154062 | 1.35E-18 | positive |
| SRF | AL928921.2 | 0.557192047 | 5.64E-35 | positive |
| STMN2 | AL928921.2 | 0.421124361 | 3.85E-19 | positive |
| TGFB1 | AL928921.2 | 0.424504622 | 1.87E-19 | positive |
| TUBA1A | AL928921.2 | 0.720642039 | 3.28E-67 | positive |
| VCL | AL928921.2 | 0.628025488 | 1.39E-46 | positive |
| VIM | AL928921.2 | 0.479307046 | 4.70E-25 | positive |
| CUEDC2 | NAPA-AS1 | 0.410221712 | 3.74E-18 | positive |
| TP63 | MIR205HG | 0.798922825 | 1.44E-92 | positive |
| RASA2 | AC004837.2 | 0.432097051 | 3.60E-20 | positive |
| MLANA | AC020915.2 | 0.42500651 | 1.68E-19 | positive |
| ROCK1 | AC020915.2 | 0.405984832 | 8.83E-18 | positive |
| RASA2 | AL360181.2 | 0.46052762 | 5.09E-23 | positive |
| MLANA | AL132989.1 | 0.561336629 | 1.40E-35 | positive |
| PIKFYVE | AL132989.1 | 0.481984711 | 2.36E-25 | positive |
| RASA2 | AL132989.1 | 0.601599949 | 6.50E-42 | positive |
| RNF146 | AL132989.1 | 0.435763106 | 1.60E-20 | positive |
| ROCK1 | AL132989.1 | 0.425816386 | 1.41E-19 | positive |
| ROCK1 | CAHM | 0.42679445 | 1.14E-19 | positive |
| AR | AC106897.1 | 0.631365609 | 3.30E-47 | positive |
| CAV1 | AC106897.1 | 0.432749727 | 3.11E-20 | positive |
| ITGB1 | AC106897.1 | 0.515961199 | 2.10E-29 | positive |
| MAPT | AC106897.1 | 0.543123869 | 5.48E-33 | positive |
| MYLK | AC106897.1 | 0.751742212 | 3.84E-76 | positive |
| PCDH7 | AC106897.1 | 0.517767965 | 1.24E-29 | positive |
| PRKAA2 | AC106897.1 | 0.612378402 | 9.14E-44 | positive |
| PRNP | AC106897.1 | 0.650066097 | 7.69E-51 | positive |
| RNF146 | AC106897.1 | 0.485551098 | 9.29E-26 | positive |
| ROCK1 | AC106897.1 | 0.460713105 | 4.86E-23 | positive |
| SRF | AC106897.1 | 0.544695979 | 3.32E-33 | positive |
| TUBA1A | AC106897.1 | 0.704949026 | 3.77E-63 | positive |
| VCL | AC106897.1 | 0.691599931 | 6.66E-60 | positive |
| VIM | AC106897.1 | 0.410072905 | 3.85E-18 | positive |
| MYLK | LINC00865 | 0.417387167 | 8.47E-19 | positive |
| PRKAA2 | LINC00865 | 0.473322507 | 2.16E-24 | positive |
| AR | CTD-2201I18.1 | 0.50321391 | 7.84E-28 | positive |
| CDH1 | CTD-2201I18.1 | -0.401973767 | 1.97E-17 | negative |
| MYLK | CTD-2201I18.1 | 0.464829314 | 1.78E-23 | positive |
| PRNP | CTD-2201I18.1 | 0.41567022 | 1.21E-18 | positive |
| RNF146 | CTD-2201I18.1 | 0.414759291 | 1.47E-18 | positive |
| TUBA1A | CTD-2201I18.1 | 0.478120234 | 6.38E-25 | positive |
| VCL | CTD-2201I18.1 | 0.404733009 | 1.14E-17 | positive |
| MLANA | AC092574.2 | 0.566672031 | 2.28E-36 | positive |
| ROCK1 | AC092574.2 | 0.401901383 | 2.00E-17 | positive |
| RASA2 | AL031670.1 | 0.405000072 | 1.08E-17 | positive |
| AR | LINC02593 | 0.498231361 | 3.10E-27 | positive |
| RNF146 | AC010201.1 | 0.408659521 | 5.14E-18 | positive |
| MLANA | AC010530.1 | 0.428201673 | 8.43E-20 | positive |
| ROCK1 | AC010530.1 | 0.400700396 | 2.54E-17 | positive |
| TF | AP001065.2 | 0.583817849 | 5.24E-39 | positive |
| MLANA | AL353804.2 | 0.40950293 | 4.33E-18 | positive |
| AR | BNC2-AS1 | 0.627662082 | 1.62E-46 | positive |
| CAV1 | BNC2-AS1 | 0.431565335 | 4.04E-20 | positive |
| CDH1 | BNC2-AS1 | -0.447991245 | 9.90E-22 | negative |
| KIF2C | BNC2-AS1 | -0.417485061 | 8.30E-19 | negative |
| MAPT | BNC2-AS1 | 0.462107453 | 3.47E-23 | positive |
| MYLK | BNC2-AS1 | 0.657466211 | 2.38E-52 | positive |
| PCDH7 | BNC2-AS1 | 0.472364453 | 2.75E-24 | positive |
| PRKAA2 | BNC2-AS1 | 0.477049693 | 8.39E-25 | positive |
| PRNP | BNC2-AS1 | 0.639402185 | 9.73E-49 | positive |
| RNF146 | BNC2-AS1 | 0.42623314 | 1.29E-19 | positive |
| S100B | BNC2-AS1 | 0.402977992 | 1.61E-17 | positive |
| SRF | BNC2-AS1 | 0.494328136 | 8.97E-27 | positive |
| TUBA1A | BNC2-AS1 | 0.768502468 | 1.59E-81 | positive |
| VCL | BNC2-AS1 | 0.585293559 | 3.06E-39 | positive |
| VIM | BNC2-AS1 | 0.607375215 | 6.75E-43 | positive |
| RAC1 | LINC00853 | 0.430993448 | 4.58E-20 | positive |
| AR | AP001107.5 | 0.776994712 | 1.97E-84 | positive |
| CAV1 | AP001107.5 | 0.53057295 | 2.72E-31 | positive |
| ITGB1 | AP001107.5 | 0.511802563 | 6.95E-29 | positive |
| MAPT | AP001107.5 | 0.751953683 | 3.31E-76 | positive |
| MYLK | AP001107.5 | 0.937099392 | 1.64E-189 | positive |
| PCDH7 | AP001107.5 | 0.616889781 | 1.46E-44 | positive |
| PRKAA2 | AP001107.5 | 0.691775514 | 6.06E-60 | positive |
| PRNP | AP001107.5 | 0.800267138 | 4.24E-93 | positive |
| RNF146 | AP001107.5 | 0.403221236 | 1.54E-17 | positive |
| S100B | AP001107.5 | 0.5124802 | 5.72E-29 | positive |
| SRF | AP001107.5 | 0.754358185 | 5.93E-77 | positive |
| STMN2 | AP001107.5 | 0.426047169 | 1.34E-19 | positive |
| TUBA1A | AP001107.5 | 0.862341435 | 2.99E-123 | positive |
| VCL | AP001107.5 | 0.808725061 | 1.54E-96 | positive |
| MLANA | AC078778.2 | 0.431689764 | 3.93E-20 | positive |
| AR | NR2F2-AS1 | 0.545647346 | 2.45E-33 | positive |
| MYLK | NR2F2-AS1 | 0.520507649 | 5.55E-30 | positive |
| PCDH7 | NR2F2-AS1 | 0.494694827 | 8.12E-27 | positive |
| PRKAA2 | NR2F2-AS1 | 0.447441578 | 1.13E-21 | positive |
| PRNP | NR2F2-AS1 | 0.435862148 | 1.56E-20 | positive |
| RNF146 | NR2F2-AS1 | 0.441476398 | 4.42E-21 | positive |
| ROCK1 | NR2F2-AS1 | 0.426993846 | 1.10E-19 | positive |
| TUBA1A | NR2F2-AS1 | 0.478774343 | 5.39E-25 | positive |
| VCL | NR2F2-AS1 | 0.492867879 | 1.33E-26 | positive |
| VIM | NR2F2-AS1 | 0.403264486 | 1.53E-17 | positive |
| MLANA | AC007496.1 | 0.531352505 | 2.14E-31 | positive |
| RASA2 | AC007496.1 | 0.448005822 | 9.87E-22 | positive |
| ROCK2 | AC124067.4 | 0.42361603 | 2.27E-19 | positive |
| ROCK1 | AP003467.1 | 0.442835288 | 3.24E-21 | positive |
| MLANA | AL049840.1 | 0.457745604 | 9.93E-23 | positive |
| RASA2 | AL049840.1 | 0.421317156 | 3.70E-19 | positive |
| ROCK1 | AL049840.1 | 0.400290361 | 2.76E-17 | positive |
| MYL2 | PSORS1C3 | 0.482160417 | 2.25E-25 | positive |
| MYL2 | AP000692.1 | 0.416110978 | 1.11E-18 | positive |
| RASA2 | AP000692.1 | 0.471720619 | 3.23E-24 | positive |
| RUBCN | AC096733.2 | 0.439042074 | 7.66E-21 | positive |
| TGFB1 | AC096733.2 | 0.480060606 | 3.87E-25 | positive |
| RUBCN | AC090587.2 | 0.461626521 | 3.90E-23 | positive |
| MLANA | AC004223.2 | 0.402635551 | 1.73E-17 | positive |
| MLANA | AL031717.1 | 0.476985806 | 8.52E-25 | positive |
| RASA2 | AL031717.1 | 0.495995858 | 5.71E-27 | positive |
| MYL2 | AC126773.1 | 0.453270069 | 2.88E-22 | positive |
| MLANA | AC008737.1 | 0.503132573 | 8.02E-28 | positive |
| RNF146 | AC008737.1 | 0.40129367 | 2.26E-17 | positive |
| ROCK1 | AC008737.1 | 0.408510563 | 5.30E-18 | positive |
| MLANA | AC008735.4 | 0.452918391 | 3.13E-22 | positive |
| AR | AC079313.2 | 0.496897699 | 4.46E-27 | positive |
| CAV1 | AC079313.2 | 0.452588171 | 3.38E-22 | positive |
| ITGB1 | AC079313.2 | 0.481497363 | 2.67E-25 | positive |
| MAPT | AC079313.2 | 0.633135878 | 1.53E-47 | positive |
| MYLK | AC079313.2 | 0.700711803 | 4.23E-62 | positive |
| PRKAA2 | AC079313.2 | 0.47233856 | 2.77E-24 | positive |
| PRNP | AC079313.2 | 0.581767229 | 1.11E-38 | positive |
| S100B | AC079313.2 | 0.465716075 | 1.44E-23 | positive |
| SRF | AC079313.2 | 0.540724707 | 1.17E-32 | positive |
| TUBA1A | AC079313.2 | 0.696989743 | 3.42E-61 | positive |
| VCL | AC079313.2 | 0.630436739 | 4.93E-47 | positive |
| CUEDC2 | AC100810.1 | 0.468056224 | 8.05E-24 | positive |
| DIAPH1 | AC100810.1 | -0.438983233 | 7.76E-21 | negative |
| MAP1LC3A | AC100810.1 | 0.451770564 | 4.10E-22 | positive |
| TUBA1A | AC100810.1 | 0.447669358 | 1.07E-21 | positive |
| RNF146 | LAMC1-AS1 | 0.408666947 | 5.13E-18 | positive |
| CDKN2A | TDRKH-AS1 | 0.44560344 | 1.72E-21 | positive |
| MLANA | AC009090.6 | 0.417655146 | 8.01E-19 | positive |
| PIKFYVE | AC009090.6 | 0.41922349 | 5.76E-19 | positive |
| RASA2 | AC009090.6 | 0.518791247 | 9.18E-30 | positive |
| ROCK1 | AL034417.4 | 0.406703686 | 7.64E-18 | positive |
| CDC20 | AL157392.3 | -0.40519649 | 1.04E-17 | negative |
| MLANA | AL157392.3 | 0.544334594 | 3.73E-33 | positive |
| PIKFYVE | AL157392.3 | 0.451742031 | 4.13E-22 | positive |
| RASA2 | AL157392.3 | 0.517123682 | 1.49E-29 | positive |
| RNF146 | AL157392.3 | 0.460693376 | 4.89E-23 | positive |
| ROCK1 | AL157392.3 | 0.449070901 | 7.71E-22 | positive |
| MLANA | CCDC18-AS1 | 0.444277204 | 2.33E-21 | positive |
| RASA2 | CCDC18-AS1 | 0.509917846 | 1.19E-28 | positive |
| RNF146 | CCDC18-AS1 | 0.402323103 | 1.84E-17 | positive |
| ROCK1 | CCDC18-AS1 | 0.408489848 | 5.32E-18 | positive |
| AR | AC024075.1 | 0.453095276 | 3.00E-22 | positive |
| CDC20 | AC024075.1 | -0.401029557 | 2.38E-17 | negative |
| KIF2C | AC024075.1 | -0.45503358 | 1.90E-22 | negative |
| MAD2L1 | AC024075.1 | -0.410459147 | 3.56E-18 | negative |
| MLANA | AC024075.1 | 0.493031799 | 1.27E-26 | positive |
| MYLK | AC024075.1 | 0.480556541 | 3.41E-25 | positive |
| ROCK1 | AC024075.1 | 0.435795212 | 1.59E-20 | positive |
| SPRN | AC024075.1 | 0.401323347 | 2.25E-17 | positive |
| TUBA1A | AC024075.1 | 0.456980498 | 1.19E-22 | positive |
| VCL | AC024075.1 | 0.434810221 | 1.97E-20 | positive |
| RASA2 | AP000763.3 | 0.427647342 | 9.51E-20 | positive |
| CXCL8 | MIR3945HG | 0.430548887 | 5.05E-20 | positive |
| AR | AL445426.1 | 0.572418059 | 3.10E-37 | positive |
| CDC20 | AL445426.1 | -0.415324216 | 1.30E-18 | negative |
| KIF2C | AL445426.1 | -0.454111896 | 2.36E-22 | negative |
| MAD2L1 | AL445426.1 | -0.421223995 | 3.77E-19 | negative |
| MYLK | AL445426.1 | 0.491377598 | 1.98E-26 | positive |
| PRNP | AL445426.1 | 0.421163055 | 3.82E-19 | positive |
| RNF146 | AL445426.1 | 0.401476899 | 2.18E-17 | positive |
| TUBA1A | AL445426.1 | 0.483135906 | 1.75E-25 | positive |
| VCL | AL445426.1 | 0.459161568 | 7.07E-23 | positive |
| VIM | AL445426.1 | 0.404336587 | 1.23E-17 | positive |
| MLANA | LIMS1-AS1 | 0.447172875 | 1.20E-21 | positive |
| RASA2 | LIMS1-AS1 | 0.494647685 | 8.22E-27 | positive |
| RHOA | AC074117.1 | -0.408658865 | 5.14E-18 | negative |
| MLANA | AC005154.4 | 0.525651831 | 1.20E-30 | positive |
| RASA2 | AC005154.4 | 0.422489083 | 2.88E-19 | positive |
| MLANA | AL138787.2 | 0.525106671 | 1.42E-30 | positive |
| RASA2 | AL138787.2 | 0.444216298 | 2.36E-21 | positive |
| AR | AC036108.3 | 0.605799994 | 1.26E-42 | positive |
| CAV1 | AC036108.3 | 0.486830786 | 6.63E-26 | positive |
| ITGB1 | AC036108.3 | 0.614696875 | 3.57E-44 | positive |
| MAPT | AC036108.3 | 0.618381912 | 7.90E-45 | positive |
| MYLK | AC036108.3 | 0.864879007 | 8.70E-125 | positive |
| PCDH7 | AC036108.3 | 0.530252611 | 3.00E-31 | positive |
| PRKAA2 | AC036108.3 | 0.650917463 | 5.18E-51 | positive |
| PRNP | AC036108.3 | 0.691661014 | 6.45E-60 | positive |
| ROCK1 | AC036108.3 | 0.402492978 | 1.78E-17 | positive |
| SRF | AC036108.3 | 0.644565937 | 9.56E-50 | positive |
| TUBA1A | AC036108.3 | 0.703675111 | 7.83E-63 | positive |
| VCL | AC036108.3 | 0.764619048 | 3.07E-80 | positive |
| MLANA | AP005131.4 | 0.491573952 | 1.88E-26 | positive |
| MLANA | AC080162.1 | 0.459978158 | 5.81E-23 | positive |
| RASA2 | AC080162.1 | 0.472167419 | 2.89E-24 | positive |
| ROCK1 | AC080162.1 | 0.457631298 | 1.02E-22 | positive |
| ROCK1 | AC008124.1 | 0.41926883 | 5.70E-19 | positive |
| AR | AP003071.4 | 0.734545838 | 4.78E-71 | positive |
| CAV1 | AP003071.4 | 0.529850694 | 3.39E-31 | positive |
| ITGB1 | AP003071.4 | 0.589166403 | 7.32E-40 | positive |
| KIF2C | AP003071.4 | -0.428185696 | 8.46E-20 | negative |
| MAPT | AP003071.4 | 0.693715128 | 2.09E-60 | positive |
| MYLK | AP003071.4 | 0.93115283 | 9.66E-182 | positive |
| PCDH7 | AP003071.4 | 0.649273528 | 1.11E-50 | positive |
| PRKAA2 | AP003071.4 | 0.693358693 | 2.55E-60 | positive |
| PRNP | AP003071.4 | 0.794345578 | 8.68E-91 | positive |
| RNF146 | AP003071.4 | 0.427047148 | 1.08E-19 | positive |
| ROCK1 | AP003071.4 | 0.418356281 | 6.91E-19 | positive |
| S100B | AP003071.4 | 0.426798769 | 1.14E-19 | positive |
| SRF | AP003071.4 | 0.715283337 | 8.58E-66 | positive |
| TUBA1A | AP003071.4 | 0.872126203 | 2.36E-129 | positive |
| VCL | AP003071.4 | 0.836005135 | 6.41E-109 | positive |
| VIM | AP003071.4 | 0.415703066 | 1.20E-18 | positive |
| RAC1 | AC018557.2 | 0.429717129 | 6.06E-20 | positive |
| RNF146 | AC120053.1 | 0.437568015 | 1.07E-20 | positive |
| MLANA | AC078962.3 | 0.421877979 | 3.28E-19 | positive |
| AR | AL162231.2 | 0.429497818 | 6.36E-20 | positive |
| MYLK | AL162231.2 | 0.415357426 | 1.29E-18 | positive |
| TUBA1A | AL162231.2 | 0.421483315 | 3.57E-19 | positive |
| MLANA | AP4B1-AS1 | 0.405107197 | 1.05E-17 | positive |
| RASA2 | AP4B1-AS1 | 0.509810068 | 1.23E-28 | positive |
| IL6 | LINC01235 | 0.413424528 | 1.93E-18 | positive |
| PDPN | LINC01235 | 0.518082896 | 1.13E-29 | positive |
| VIM | LINC01235 | 0.511990259 | 6.58E-29 | positive |
| STMN2 | AC006504.8 | 0.449818435 | 6.47E-22 | positive |
| MLANA | AC011479.3 | 0.457553041 | 1.04E-22 | positive |
| RASA2 | AC022400.5 | 0.401065798 | 2.36E-17 | positive |
| NTS | LINC01376 | 0.408888059 | 4.90E-18 | positive |
| RND3 | SMILR | 0.433143034 | 2.86E-20 | positive |
| TP63 | SMILR | 0.540773666 | 1.15E-32 | positive |
| MLANA | AC137932.1 | 0.459688173 | 6.23E-23 | positive |
| RASA2 | AC137932.1 | 0.446493853 | 1.40E-21 | positive |
| AR | AC107959.1 | 0.711103133 | 1.04E-64 | positive |
| CDH1 | AC107959.1 | -0.485352675 | 9.78E-26 | negative |
| KIF2C | AC107959.1 | -0.419882106 | 5.01E-19 | negative |
| MAD2L1 | AC107959.1 | -0.433850232 | 2.44E-20 | negative |
| MAPT | AC107959.1 | 0.551084553 | 4.22E-34 | positive |
| MYLK | AC107959.1 | 0.704392675 | 5.19E-63 | positive |
| PCDH7 | AC107959.1 | 0.519581183 | 7.28E-30 | positive |
| PRKAA2 | AC107959.1 | 0.520732162 | 5.19E-30 | positive |
| PRNP | AC107959.1 | 0.616331563 | 1.83E-44 | positive |
| RNF146 | AC107959.1 | 0.458252916 | 8.80E-23 | positive |
| S100B | AC107959.1 | 0.450006752 | 6.20E-22 | positive |
| SRF | AC107959.1 | 0.532090814 | 1.71E-31 | positive |
| TUBA1A | AC107959.1 | 0.70217331 | 1.85E-62 | positive |
| VCL | AC107959.1 | 0.631389081 | 3.27E-47 | positive |
| VIM | AC107959.1 | 0.422662326 | 2.78E-19 | positive |
| AR | SNHG14 | 0.638449155 | 1.49E-48 | positive |
| ITGB1 | SNHG14 | 0.415893798 | 1.16E-18 | positive |
| MAPT | SNHG14 | 0.596168132 | 5.25E-41 | positive |
| MYLK | SNHG14 | 0.701726972 | 2.38E-62 | positive |
| PCDH7 | SNHG14 | 0.473280422 | 2.18E-24 | positive |
| PRKAA2 | SNHG14 | 0.624304757 | 6.71E-46 | positive |
| PRNP | SNHG14 | 0.602095395 | 5.37E-42 | positive |
| RNF146 | SNHG14 | 0.463143137 | 2.70E-23 | positive |
| ROCK1 | SNHG14 | 0.433582793 | 2.59E-20 | positive |
| SRF | SNHG14 | 0.516420831 | 1.83E-29 | positive |
| TUBA1A | SNHG14 | 0.591211112 | 3.41E-40 | positive |
| VCL | SNHG14 | 0.600289116 | 1.08E-41 | positive |
| AR | AC015922.3 | 0.442287839 | 3.67E-21 | positive |
| RNF146 | AC015922.3 | 0.401842231 | 2.03E-17 | positive |
| UVRAG | AC015922.3 | 0.412003055 | 2.59E-18 | positive |
| RASA2 | AC109460.2 | 0.471157477 | 3.72E-24 | positive |
| MLANA | SLC16A1-AS1 | 0.409314352 | 4.50E-18 | positive |
| ROCK1 | SLC16A1-AS1 | 0.406956694 | 7.26E-18 | positive |
| MYL2 | AC011445.2 | 0.402914475 | 1.64E-17 | positive |
| PIKFYVE | AC068790.3 | 0.407792521 | 6.13E-18 | positive |
| RASA2 | AC068790.3 | 0.4744253 | 1.63E-24 | positive |
| MLANA | AP003392.1 | 0.442470515 | 3.52E-21 | positive |
| MYLK | AC024075.2 | 0.425120926 | 1.64E-19 | positive |
| RNF146 | AC024075.2 | 0.436485201 | 1.36E-20 | positive |
| ROCK1 | AC024075.2 | 0.41153934 | 2.85E-18 | positive |
| TUBA1A | AC024075.2 | 0.409452616 | 4.37E-18 | positive |
| MLANA | AC087749.1 | 0.410231649 | 3.73E-18 | positive |
| RASA2 | AC087749.1 | 0.40083139 | 2.48E-17 | positive |
| TF | AC123912.4 | 0.443073009 | 3.07E-21 | positive |
| MAP1LC3A | LINC00957 | 0.429077865 | 6.96E-20 | positive |
| MAPT | LINC00957 | 0.458388531 | 8.52E-23 | positive |
| MYLK | LINC00957 | 0.410476478 | 3.55E-18 | positive |
| TUBA1A | LINC00957 | 0.428713325 | 7.54E-20 | positive |
| AR | AC022034.1 | 0.4378909 | 9.92E-21 | positive |
| MYLK | AC022034.1 | 0.554889219 | 1.21E-34 | positive |
| PCDH7 | AC022034.1 | 0.486869233 | 6.56E-26 | positive |
| PRKAA2 | AC022034.1 | 0.400416713 | 2.69E-17 | positive |
| PRNP | AC022034.1 | 0.468550844 | 7.12E-24 | positive |
| SRF | AC022034.1 | 0.420619867 | 4.29E-19 | positive |
| TUBA1A | AC022034.1 | 0.461838005 | 3.70E-23 | positive |
| VCL | AC022034.1 | 0.499002343 | 2.51E-27 | positive |
| MYL2 | AC092718.6 | 0.437580559 | 1.06E-20 | positive |
| IL6 | AC037198.1 | 0.426749428 | 1.15E-19 | positive |
| PDPN | AC037198.1 | 0.477356565 | 7.75E-25 | positive |
| AR | AC007637.1 | 0.428585954 | 7.75E-20 | positive |
| MAD2L1 | AC007637.1 | -0.493981201 | 9.85E-27 | negative |
| MYLK | AC007637.1 | 0.454586323 | 2.11E-22 | positive |
| VCL | AC007637.1 | 0.438612529 | 8.44E-21 | positive |
| RUBCN | AC145124.1 | 0.437695659 | 1.04E-20 | positive |
| MLANA | AC008735.1 | 0.464036585 | 2.17E-23 | positive |
| RASA2 | AC008735.1 | 0.500910267 | 1.49E-27 | positive |
| CYBB | AC112721.2 | 0.422412958 | 2.93E-19 | positive |
| AR | AC067750.1 | 0.651760356 | 3.50E-51 | positive |
| CAV1 | AC067750.1 | 0.411561059 | 2.84E-18 | positive |
| ITGB1 | AC067750.1 | 0.467172491 | 1.00E-23 | positive |
| MAPT | AC067750.1 | 0.651078875 | 4.81E-51 | positive |
| MYLK | AC067750.1 | 0.824131435 | 2.88E-103 | positive |
| PCDH7 | AC067750.1 | 0.581669513 | 1.14E-38 | positive |
| PRKAA2 | AC067750.1 | 0.670922204 | 3.31E-55 | positive |
| PRNP | AC067750.1 | 0.67240224 | 1.57E-55 | positive |
| RNF146 | AC067750.1 | 0.500302284 | 1.76E-27 | positive |
| ROCK1 | AC067750.1 | 0.466476418 | 1.19E-23 | positive |
| SRF | AC067750.1 | 0.613491875 | 5.83E-44 | positive |
| TUBA1A | AC067750.1 | 0.709972546 | 2.02E-64 | positive |
| VCL | AC067750.1 | 0.747787489 | 6.21E-75 | positive |
| RASA2 | AC002553.2 | 0.500683443 | 1.58E-27 | positive |
| MLANA | AC018638.7 | 0.441728566 | 4.17E-21 | positive |
| MLANA | LINC01550 | 0.427422817 | 9.98E-20 | positive |
| MYL2 | AC136469.1 | 0.495962762 | 5.76E-27 | positive |
| MLANA | IGBP1-AS1 | 0.455011461 | 1.91E-22 | positive |
| RASA2 | IGBP1-AS1 | 0.446850435 | 1.29E-21 | positive |
| NUPR1 | DKFZp779M0652 | 0.441139884 | 4.77E-21 | positive |
| CAV1 | CASC8 | 0.448244846 | 9.34E-22 | positive |
| KRT7 | CASC8 | 0.552894122 | 2.34E-34 | positive |
| NTS | TP53TG1 | 0.665500116 | 4.88E-54 | positive |
| MLANA | PHACTR2-AS1 | 0.531259513 | 2.21E-31 | positive |
| TM9SF4 | OSER1-DT | 0.402626185 | 1.73E-17 | positive |
| CDC20 | AC092718.4 | 0.449258687 | 7.38E-22 | positive |
| RAC1 | AC092718.4 | 0.451926086 | 3.95E-22 | positive |
| PRNP | MIR193BHG | 0.439192793 | 7.40E-21 | positive |
| AR | AC008759.3 | 0.410381786 | 3.61E-18 | positive |
| MYLK | AC008759.3 | 0.407940949 | 5.95E-18 | positive |
| RASA2 | AC018695.4 | 0.410013134 | 3.90E-18 | positive |
| RASA2 | AL031775.2 | 0.423919393 | 2.12E-19 | positive |
| CTSK | LINC01705 | 0.520115798 | 6.22E-30 | positive |
| PDPN | LINC01705 | 0.403723774 | 1.39E-17 | positive |
| VIM | LINC01705 | 0.400946541 | 2.42E-17 | positive |
| RAC1 | B4GALT1-AS1 | 0.425412827 | 1.54E-19 | positive |
| MLANA | AL031668.1 | 0.412308291 | 2.43E-18 | positive |
| ROCK2 | NORAD | 0.455559939 | 1.67E-22 | positive |
| TM9SF4 | NORAD | 0.543738446 | 4.51E-33 | positive |
| AR | AL390955.2 | 0.475790527 | 1.16E-24 | positive |
| MYLK | AL390955.2 | 0.507314535 | 2.49E-28 | positive |
| PRKAA2 | AL390955.2 | 0.4118266 | 2.69E-18 | positive |
| PRNP | AL390955.2 | 0.48271779 | 1.95E-25 | positive |
| SRF | AL390955.2 | 0.43932136 | 7.19E-21 | positive |
| TUBA1A | AL390955.2 | 0.496273343 | 5.29E-27 | positive |
| VCL | AL390955.2 | 0.428747616 | 7.48E-20 | positive |
| MLANA | AL022328.2 | 0.447719913 | 1.05E-21 | positive |
| RASA2 | AL022328.2 | 0.427557475 | 9.69E-20 | positive |
| ARHGAP36 | AC127070.2 | 0.402238839 | 1.87E-17 | positive |
| MYLK | AC127070.2 | 0.406632855 | 7.75E-18 | positive |
| RNF146 | AC127070.2 | 0.45149854 | 4.37E-22 | positive |
| ROCK1 | AC127070.2 | 0.444815711 | 2.06E-21 | positive |
| CTTN | AP000487.1 | 0.469591249 | 5.50E-24 | positive |
| MLANA | AL022238.2 | 0.420911881 | 4.03E-19 | positive |
| IL10 | DBH-AS1 | 0.597414578 | 3.26E-41 | positive |
| DIAPH1 | AC012306.2 | -0.41843706 | 6.80E-19 | negative |
| MLANA | AC007390.2 | 0.471798343 | 3.17E-24 | positive |
| RASA2 | AC007390.2 | 0.554101278 | 1.57E-34 | positive |
| AR | AC084033.3 | 0.526866152 | 8.35E-31 | positive |
| CDH1 | AC084033.3 | -0.402229027 | 1.88E-17 | negative |
| CUEDC2 | AC084033.3 | 0.432322787 | 3.42E-20 | positive |
| MAPT | AC084033.3 | 0.541998902 | 7.83E-33 | positive |
| MYLK | AC084033.3 | 0.528928551 | 4.48E-31 | positive |
| PRKAA2 | AC084033.3 | 0.516727214 | 1.68E-29 | positive |
| PRNP | AC084033.3 | 0.586931304 | 1.67E-39 | positive |
| RNF146 | AC084033.3 | 0.431200567 | 4.38E-20 | positive |
| SNCA | AC084033.3 | 0.422932701 | 2.62E-19 | positive |
| TUBA1A | AC084033.3 | 0.63212536 | 2.38E-47 | positive |
| VCL | AC084033.3 | 0.40468269 | 1.15E-17 | positive |
| VIM | AC084033.3 | 0.449462451 | 7.04E-22 | positive |
| AR | ZNF582-AS1 | 0.591892669 | 2.64E-40 | positive |
| MAPT | ZNF582-AS1 | 0.46470308 | 1.84E-23 | positive |
| MYLK | ZNF582-AS1 | 0.592569108 | 2.05E-40 | positive |
| PCDH7 | ZNF582-AS1 | 0.423955984 | 2.11E-19 | positive |
| PRKAA2 | ZNF582-AS1 | 0.494116743 | 9.49E-27 | positive |
| PRNP | ZNF582-AS1 | 0.532711793 | 1.41E-31 | positive |
| RNF146 | ZNF582-AS1 | 0.430491985 | 5.11E-20 | positive |
| SRF | ZNF582-AS1 | 0.443366206 | 2.87E-21 | positive |
| TUBA1A | ZNF582-AS1 | 0.593797135 | 1.29E-40 | positive |
| VCL | ZNF582-AS1 | 0.453127503 | 2.98E-22 | positive |
| VIM | ZNF582-AS1 | 0.426925481 | 1.11E-19 | positive |
| MLANA | AC020913.1 | 0.491896942 | 1.72E-26 | positive |
| RASA2 | AC020913.1 | 0.453679419 | 2.61E-22 | positive |
| MLANA | AC074138.1 | 0.41283103 | 2.19E-18 | positive |
| MLANA | AL450344.2 | 0.526537046 | 9.22E-31 | positive |
| RASA2 | AL450344.2 | 0.448375895 | 9.06E-22 | positive |
| CDC42 | AC079466.1 | 0.457753637 | 9.92E-23 | positive |
| CYBB | AL365361.1 | 0.472246939 | 2.83E-24 | positive |
| RASA2 | AL365361.1 | 0.409137692 | 4.66E-18 | positive |
| MLANA | AL512770.1 | 0.463049236 | 2.76E-23 | positive |
| RASA2 | AL512770.1 | 0.41963331 | 5.28E-19 | positive |
| RAC1 | AC103769.1 | 0.403406533 | 1.48E-17 | positive |
| AR | AC090152.1 | 0.429509056 | 6.34E-20 | positive |
| MYLK | AC090152.1 | 0.444657008 | 2.14E-21 | positive |
| SNCA | AC090152.1 | 0.431879466 | 3.77E-20 | positive |
| TUBA1A | AC090152.1 | 0.448585141 | 8.63E-22 | positive |
| DIAPH1 | LINC00476 | -0.415688578 | 1.21E-18 | negative |
| RASA2 | AC100778.2 | 0.405442386 | 9.85E-18 | positive |
| RASA2 | AC132872.3 | 0.437867309 | 9.98E-21 | positive |
| RASA2 | ZBTB40-IT1 | 0.427250355 | 1.04E-19 | positive |
| MLANA | AL049552.1 | 0.419563297 | 5.36E-19 | positive |
| PIKFYVE | AL049552.1 | 0.412757786 | 2.22E-18 | positive |
| RASA2 | AL049552.1 | 0.419389157 | 5.56E-19 | positive |
| AR | ZNF710-AS1 | 0.610258327 | 2.14E-43 | positive |
| CAV1 | ZNF710-AS1 | 0.417357085 | 8.52E-19 | positive |
| MAPT | ZNF710-AS1 | 0.610832609 | 1.70E-43 | positive |
| MYLK | ZNF710-AS1 | 0.779522874 | 2.54E-85 | positive |
| PCDH7 | ZNF710-AS1 | 0.404307364 | 1.24E-17 | positive |
| PRKAA2 | ZNF710-AS1 | 0.612122916 | 1.01E-43 | positive |
| PRNP | ZNF710-AS1 | 0.627670347 | 1.61E-46 | positive |
| SRF | ZNF710-AS1 | 0.58259663 | 8.18E-39 | positive |
| TUBA1A | ZNF710-AS1 | 0.701406807 | 2.85E-62 | positive |
| VCL | ZNF710-AS1 | 0.641060026 | 4.64E-49 | positive |
| MYL2 | AC087721.1 | 0.402737593 | 1.69E-17 | positive |
| AR | AC103740.1 | 0.476354294 | 1.00E-24 | positive |
| ITGB1 | AC103740.1 | 0.411825631 | 2.69E-18 | positive |
| MAPT | AC103740.1 | 0.439466023 | 6.96E-21 | positive |
| MYLK | AC103740.1 | 0.673506403 | 8.99E-56 | positive |
| PCDH7 | AC103740.1 | 0.509203866 | 1.46E-28 | positive |
| PRKAA2 | AC103740.1 | 0.492632958 | 1.42E-26 | positive |
| PRNP | AC103740.1 | 0.48470285 | 1.16E-25 | positive |
| SRF | AC103740.1 | 0.606809166 | 8.45E-43 | positive |
| TUBA1A | AC103740.1 | 0.503558421 | 7.13E-28 | positive |
| VCL | AC103740.1 | 0.57299693 | 2.53E-37 | positive |
| MLANA | AC245884.8 | 0.40081484 | 2.48E-17 | positive |
| CTSB | AF131215.6 | 0.41341612 | 1.94E-18 | positive |
| TF | AC115619.1 | 0.781975091 | 3.39E-86 | positive |
| MLANA | AC012358.1 | 0.405046791 | 1.07E-17 | positive |
| MYL2 | AC011462.3 | 0.464084742 | 2.14E-23 | positive |
| AR | AC012085.2 | 0.555248997 | 1.08E-34 | positive |
| MAPT | AC012085.2 | 0.574707054 | 1.38E-37 | positive |
| MYLK | AC012085.2 | 0.624176175 | 7.08E-46 | positive |
| PCDH7 | AC012085.2 | 0.42099463 | 3.96E-19 | positive |
| PRKAA2 | AC012085.2 | 0.442326377 | 3.64E-21 | positive |
| PRNP | AC012085.2 | 0.516243258 | 1.93E-29 | positive |
| S100B | AC012085.2 | 0.47042575 | 4.47E-24 | positive |
| SRF | AC012085.2 | 0.483664697 | 1.52E-25 | positive |
| TUBA1A | AC012085.2 | 0.607933026 | 5.41E-43 | positive |
| VCL | AC012085.2 | 0.552607343 | 2.57E-34 | positive |
| SPRN | AC102953.2 | 0.410067013 | 3.86E-18 | positive |
| MTMR7 | AC012213.3 | 0.423323655 | 2.41E-19 | positive |
| CYBB | MMP2-AS1 | 0.433617334 | 2.57E-20 | positive |
| NTS | AC093297.2 | 0.430407261 | 5.21E-20 | positive |
| ROCK1 | AC002558.2 | 0.404098945 | 1.29E-17 | positive |
| ROCK1 | AC067852.3 | 0.439019141 | 7.70E-21 | positive |
| LPAR2 | AL139246.5 | 0.425239372 | 1.60E-19 | positive |
| TF | HPN-AS1 | 0.51336679 | 4.43E-29 | positive |
| RASA2 | AP000442.2 | 0.408104135 | 5.75E-18 | positive |
| MLANA | DIP2A-IT1 | 0.445742118 | 1.67E-21 | positive |
| RASA2 | DIP2A-IT1 | 0.430796724 | 4.78E-20 | positive |
| TP63 | AC103563.7 | 0.514484702 | 3.21E-29 | positive |
| MLANA | Z99127.1 | 0.411343594 | 2.97E-18 | positive |
| AR | MIR100HG | 0.73674591 | 1.12E-71 | positive |
| CAV1 | MIR100HG | 0.48535478 | 9.78E-26 | positive |
| CDC20 | MIR100HG | -0.407911915 | 5.98E-18 | negative |
| CDH1 | MIR100HG | -0.467795918 | 8.59E-24 | negative |
| ITGB1 | MIR100HG | 0.571395346 | 4.43E-37 | positive |
| KIF2C | MIR100HG | -0.478643749 | 5.58E-25 | negative |
| MAD2L1 | MIR100HG | -0.409505654 | 4.32E-18 | negative |
| MAPT | MIR100HG | 0.58846806 | 9.48E-40 | positive |
| MYLK | MIR100HG | 0.85739681 | 2.42E-120 | positive |
| PCDH7 | MIR100HG | 0.66896652 | 8.79E-55 | positive |
| PRKAA2 | MIR100HG | 0.629277313 | 8.11E-47 | positive |
| PRNP | MIR100HG | 0.786965471 | 5.18E-88 | positive |
| RNF146 | MIR100HG | 0.50241189 | 9.80E-28 | positive |
| S100B | MIR100HG | 0.421060928 | 3.90E-19 | positive |
| SRF | MIR100HG | 0.60377046 | 2.79E-42 | positive |
| STMN2 | MIR100HG | 0.444322958 | 2.31E-21 | positive |
| TUBA1A | MIR100HG | 0.82027432 | 1.60E-101 | positive |
| VCL | MIR100HG | 0.761724208 | 2.70E-79 | positive |
| VIM | MIR100HG | 0.509779821 | 1.24E-28 | positive |
| RASA2 | AC135050.3 | 0.423584849 | 2.28E-19 | positive |
| MLANA | AC098484.4 | 0.402791998 | 1.68E-17 | positive |
| PIKFYVE | AC098484.4 | 0.443885671 | 2.55E-21 | positive |
| RASA2 | AC098484.4 | 0.487223282 | 5.98E-26 | positive |
| MLANA | AC012435.2 | 0.451042756 | 4.86E-22 | positive |
| KIF2C | C6orf99 | 0.404917818 | 1.09E-17 | positive |
| MLANA | SKAP1-AS1 | 0.401384354 | 2.22E-17 | positive |
| RASA2 | SKAP1-AS1 | 0.419416492 | 5.53E-19 | positive |
| ROCK1 | THUMPD3-AS1 | 0.410298528 | 3.68E-18 | positive |
| CYBB | HLA-DQB1-AS1 | 0.459331196 | 6.79E-23 | positive |
| RASA2 | HLA-DQB1-AS1 | 0.414073239 | 1.69E-18 | positive |
| RAC1 | AL355353.1 | 0.444139606 | 2.41E-21 | positive |
| RASA2 | AL049840.4 | 0.405008637 | 1.08E-17 | positive |
| MLANA | AC137932.3 | 0.402787476 | 1.68E-17 | positive |
| RASA2 | AC137932.3 | 0.424770283 | 1.77E-19 | positive |
| MYL2 | AL160163.1 | 0.400719209 | 2.53E-17 | positive |
| MLANA | AC090519.2 | 0.450822883 | 5.12E-22 | positive |
| ROCK1 | AC002553.1 | 0.417565765 | 8.16E-19 | positive |
| RAC1 | ARHGAP27P1-BPTFP1-KPNA2P3 | 0.419825383 | 5.07E-19 | positive |
| CDC42 | Z98257.1 | 0.418051596 | 7.37E-19 | positive |
| CTSK | MSC-AS1 | 0.614175734 | 4.42E-44 | positive |
| PDPN | MSC-AS1 | 0.57059118 | 5.87E-37 | positive |
| PRND | MSC-AS1 | 0.471900927 | 3.09E-24 | positive |
| TGFB1 | MSC-AS1 | 0.403233413 | 1.53E-17 | positive |
| VIM | MSC-AS1 | 0.61602957 | 2.08E-44 | positive |
| MLANA | PCCA-AS1 | 0.408684388 | 5.11E-18 | positive |
| RASA2 | AL359921.1 | 0.423610809 | 2.27E-19 | positive |
| AR | LINC00702 | 0.417163902 | 8.88E-19 | positive |
| ITGB1 | LINC00702 | 0.577765742 | 4.66E-38 | positive |
| MAPT | LINC00702 | 0.423987643 | 2.09E-19 | positive |
| MYLK | LINC00702 | 0.735984234 | 1.86E-71 | positive |
| PCDH7 | LINC00702 | 0.512451573 | 5.77E-29 | positive |
| PRKAA2 | LINC00702 | 0.626991196 | 2.15E-46 | positive |
| PRNP | LINC00702 | 0.584559193 | 4.00E-39 | positive |
| SRF | LINC00702 | 0.517645938 | 1.28E-29 | positive |
| TUBA1A | LINC00702 | 0.667297918 | 2.01E-54 | positive |
| VCL | LINC00702 | 0.66381002 | 1.12E-53 | positive |
| AR | AC120049.1 | 0.644872102 | 8.32E-50 | positive |
| CDH1 | AC120049.1 | -0.471773658 | 3.19E-24 | negative |
| KIF2C | AC120049.1 | -0.481322164 | 2.80E-25 | negative |
| MAD2L1 | AC120049.1 | -0.482456853 | 2.08E-25 | negative |
| MAPT | AC120049.1 | 0.500374515 | 1.72E-27 | positive |
| MYLK | AC120049.1 | 0.643380158 | 1.64E-49 | positive |
| PCDH7 | AC120049.1 | 0.519372898 | 7.74E-30 | positive |
| PRKAA2 | AC120049.1 | 0.485393175 | 9.68E-26 | positive |
| PRNP | AC120049.1 | 0.563082473 | 7.77E-36 | positive |
| RNF146 | AC120049.1 | 0.44506416 | 1.95E-21 | positive |
| SRF | AC120049.1 | 0.487959128 | 4.92E-26 | positive |
| TGFB1 | AC120049.1 | 0.400269425 | 2.77E-17 | positive |
| TUBA1A | AC120049.1 | 0.663291133 | 1.44E-53 | positive |
| VCL | AC120049.1 | 0.553627899 | 1.84E-34 | positive |
| VIM | AC120049.1 | 0.466996521 | 1.05E-23 | positive |
| RNF146 | AC005332.3 | 0.414893729 | 1.43E-18 | positive |
| RASA2 | AC007546.1 | 0.497533739 | 3.75E-27 | positive |
| CTSS | DLGAP1-AS5 | 0.417442953 | 8.37E-19 | positive |
| GZMB | DLGAP1-AS5 | 0.599019971 | 1.76E-41 | positive |
| SRF | AL731577.2 | 0.408558696 | 5.24E-18 | positive |
| AR | AP000941.1 | 0.47217706 | 2.88E-24 | positive |
| TUBA1A | AP000941.1 | 0.418530075 | 6.66E-19 | positive |
| VCL | AP000941.1 | 0.46285081 | 2.89E-23 | positive |
| VIM | AP000941.1 | 0.433982556 | 2.37E-20 | positive |
| CTSK | AC093627.6 | 0.454782348 | 2.01E-22 | positive |
| MTMR7 | ASAP1-IT2 | 0.47785356 | 6.83E-25 | positive |
| MTUS2 | ASAP1-IT2 | 0.474383876 | 1.65E-24 | positive |
| RNF146 | AL157392.4 | 0.408562447 | 5.24E-18 | positive |
| ROCK1 | AL157392.4 | 0.411391049 | 2.94E-18 | positive |
| MLANA | AC104564.5 | 0.495517176 | 6.50E-27 | positive |
| RASA2 | AC104564.5 | 0.494947245 | 7.58E-27 | positive |
| KRT7 | AP000695.2 | 0.462164703 | 3.42E-23 | positive |
| KRT7 | LINC01615 | 0.504914584 | 4.88E-28 | positive |
| MLANA | AC104982.1 | 0.447248789 | 1.18E-21 | positive |
| MLANA | AC004492.1 | 0.439355486 | 7.14E-21 | positive |
| RASA2 | AC004492.1 | 0.427915989 | 8.97E-20 | positive |
| MLANA | FO393401.1 | 0.494368177 | 8.87E-27 | positive |
| RASA2 | FO393401.1 | 0.460017243 | 5.75E-23 | positive |
| CTSK | HHIP-AS1 | 0.414552899 | 1.53E-18 | positive |
| VIM | HHIP-AS1 | 0.413752158 | 1.81E-18 | positive |
| MTMR7 | AC022973.3 | 0.484840755 | 1.12E-25 | positive |
| MTUS2 | AC022973.3 | 0.470345652 | 4.56E-24 | positive |
| RND3 | LINC02560 | 0.425887254 | 1.39E-19 | positive |
| TP63 | LINC02560 | 0.620203553 | 3.72E-45 | positive |
| MLANA | AL592295.3 | 0.40039298 | 2.70E-17 | positive |
| RASA2 | AL592295.3 | 0.44916199 | 7.55E-22 | positive |
| AR | AC012313.5 | 0.447978769 | 9.93E-22 | positive |
| MAPT | AC012313.5 | 0.450950562 | 4.97E-22 | positive |
| MYLK | AC012313.5 | 0.494628767 | 8.27E-27 | positive |
| PRKAA2 | AC012313.5 | 0.444145327 | 2.40E-21 | positive |
| PRNP | AC012313.5 | 0.405781003 | 9.20E-18 | positive |
| TUBA1A | AC012313.5 | 0.417266049 | 8.69E-19 | positive |
| VCL | AC012313.5 | 0.433870258 | 2.43E-20 | positive |
| MTMR7 | ADGRD1-AS1 | 0.593052055 | 1.71E-40 | positive |
| MTUS2 | ADGRD1-AS1 | 0.412393409 | 2.39E-18 | positive |
| MLANA | AL357140.5 | 0.441671432 | 4.23E-21 | positive |
| AR | AC008764.2 | 0.519823919 | 6.78E-30 | positive |
| CDC20 | AC008764.2 | -0.410258037 | 3.71E-18 | negative |
| KIF2C | AC008764.2 | -0.463175792 | 2.67E-23 | negative |
| MAD2L1 | AC008764.2 | -0.411015134 | 3.17E-18 | negative |
| MAPT | AC008764.2 | 0.441060611 | 4.85E-21 | positive |
| MLANA | AC008764.2 | 0.472598268 | 2.59E-24 | positive |
| MYLK | AC008764.2 | 0.534404808 | 8.40E-32 | positive |
| PCDH7 | AC008764.2 | 0.415855433 | 1.17E-18 | positive |
| PRKAA2 | AC008764.2 | 0.411414155 | 2.93E-18 | positive |
| SPRN | AC008764.2 | 0.423668428 | 2.24E-19 | positive |
| SRF | AC008764.2 | 0.413827925 | 1.78E-18 | positive |
| TUBA1A | AC008764.2 | 0.478296803 | 6.10E-25 | positive |
| VCL | AC008764.2 | 0.493378142 | 1.16E-26 | positive |
| MLANA | AC024267.3 | 0.505740309 | 3.87E-28 | positive |
| RASA2 | AC024267.3 | 0.433991267 | 2.37E-20 | positive |
| ROCK1 | AC024267.3 | 0.415089401 | 1.37E-18 | positive |
| RASA2 | ANKRD10-IT1 | 0.441476566 | 4.42E-21 | positive |
| AR | MAGI2-AS3 | 0.755117593 | 3.43E-77 | positive |
| CAV1 | MAGI2-AS3 | 0.444018848 | 2.47E-21 | positive |
| CDC20 | MAGI2-AS3 | -0.492671386 | 1.40E-26 | negative |
| CDH1 | MAGI2-AS3 | -0.48321109 | 1.71E-25 | negative |
| CTSK | MAGI2-AS3 | 0.45356903 | 2.68E-22 | positive |
| ITGB1 | MAGI2-AS3 | 0.591771233 | 2.77E-40 | positive |
| KIF2C | MAGI2-AS3 | -0.553485827 | 1.92E-34 | negative |
| MAD2L1 | MAGI2-AS3 | -0.477962917 | 6.64E-25 | negative |
| MAPT | MAGI2-AS3 | 0.534460972 | 8.26E-32 | positive |
| MYLK | MAGI2-AS3 | 0.783960895 | 6.51E-87 | positive |
| PCDH7 | MAGI2-AS3 | 0.610782413 | 1.74E-43 | positive |
| PDPN | MAGI2-AS3 | 0.400136196 | 2.84E-17 | positive |
| PRKAA2 | MAGI2-AS3 | 0.575813722 | 9.34E-38 | positive |
| PRND | MAGI2-AS3 | 0.433803041 | 2.47E-20 | positive |
| PRNP | MAGI2-AS3 | 0.718397431 | 1.30E-66 | positive |
| RARB | MAGI2-AS3 | 0.40182768 | 2.03E-17 | positive |
| RNF146 | MAGI2-AS3 | 0.515598214 | 2.33E-29 | positive |
| ROCK1 | MAGI2-AS3 | 0.416005191 | 1.13E-18 | positive |
| S100B | MAGI2-AS3 | 0.408098146 | 5.76E-18 | positive |
| SRF | MAGI2-AS3 | 0.545655613 | 2.44E-33 | positive |
| STMN2 | MAGI2-AS3 | 0.405572998 | 9.60E-18 | positive |
| TUBA1A | MAGI2-AS3 | 0.780679836 | 9.85E-86 | positive |
| VCL | MAGI2-AS3 | 0.72802505 | 3.22E-69 | positive |
| VIM | MAGI2-AS3 | 0.592716823 | 1.94E-40 | positive |
| MLANA | AL008721.2 | 0.431593613 | 4.02E-20 | positive |
| MLANA | AC138207.4 | 0.415201979 | 1.34E-18 | positive |
| PTK2 | AC083843.2 | 0.447443466 | 1.12E-21 | positive |
| ROCK1 | AC106037.2 | 0.420349041 | 4.54E-19 | positive |
| MLANA | FOXP1-AS1 | 0.418572484 | 6.60E-19 | positive |
| AR | HAND2-AS1 | 0.757345621 | 6.82E-78 | positive |
| CAV1 | HAND2-AS1 | 0.507066971 | 2.67E-28 | positive |
| ITGB1 | HAND2-AS1 | 0.587588106 | 1.31E-39 | positive |
| MAPT | HAND2-AS1 | 0.653914405 | 1.28E-51 | positive |
| MYLK | HAND2-AS1 | 0.959654268 | 4.99E-228 | positive |
| PCDH7 | HAND2-AS1 | 0.621986451 | 1.77E-45 | positive |
| PRKAA2 | HAND2-AS1 | 0.748836398 | 2.98E-75 | positive |
| PRNP | HAND2-AS1 | 0.80047322 | 3.51E-93 | positive |
| RNF146 | HAND2-AS1 | 0.4141579 | 1.66E-18 | positive |
| S100B | HAND2-AS1 | 0.418881764 | 6.19E-19 | positive |
| SRF | HAND2-AS1 | 0.73029587 | 7.54E-70 | positive |
| TUBA1A | HAND2-AS1 | 0.824058778 | 3.11E-103 | positive |
| VCL | HAND2-AS1 | 0.826696927 | 1.88E-104 | positive |
| PIKFYVE | NADK2-AS1 | 0.436912061 | 1.24E-20 | positive |
| MYL2 | AC011471.2 | 0.437052334 | 1.20E-20 | positive |
| RASA2 | LINC01176 | 0.460580026 | 5.02E-23 | positive |
| MLANA | AL021707.4 | 0.402854296 | 1.66E-17 | positive |
| ROCK1 | ZNF790-AS1 | 0.426685807 | 1.17E-19 | positive |
| AR | AL158212.3 | 0.417414698 | 8.42E-19 | positive |
| MAPT | AL158212.3 | 0.49930679 | 2.31E-27 | positive |
| MLANA | AL158212.3 | 0.426301603 | 1.27E-19 | positive |
| MYLK | AL158212.3 | 0.532866636 | 1.35E-31 | positive |
| PRKAA2 | AL158212.3 | 0.479522902 | 4.45E-25 | positive |
| PRNP | AL158212.3 | 0.471295476 | 3.59E-24 | positive |
| RNF146 | AL158212.3 | 0.456238489 | 1.42E-22 | positive |
| SRF | AL158212.3 | 0.409642508 | 4.20E-18 | positive |
| TUBA1A | AL158212.3 | 0.49402749 | 9.72E-27 | positive |
| VCL | AL158212.3 | 0.485624897 | 9.11E-26 | positive |
| ROCK1 | AC103703.1 | 0.405334791 | 1.01E-17 | positive |
| MLANA | AC073487.1 | 0.465470976 | 1.52E-23 | positive |
| MLANA | LINC01772 | 0.501376324 | 1.31E-27 | positive |
| RASA2 | LINC01772 | 0.425407406 | 1.54E-19 | positive |
| RNF146 | LINC01772 | 0.476806752 | 8.92E-25 | positive |
| ROCK1 | LINC01772 | 0.492081163 | 1.64E-26 | positive |
| ROCK1 | AC093827.4 | 0.511365884 | 7.87E-29 | positive |
| MAP1LC3A | AL022328.4 | 0.402316396 | 1.84E-17 | positive |
| ARHGAP36 | AC022960.1 | 0.42306759 | 2.55E-19 | positive |
| MYL2 | AC022960.1 | 0.41348355 | 1.91E-18 | positive |
| MLANA | AC004832.5 | 0.416501112 | 1.02E-18 | positive |
| KRT7 | KRT7-AS | 0.629379678 | 7.76E-47 | positive |
| PIKFYVE | AC004918.3 | 0.441000382 | 4.92E-21 | positive |
| MLANA | AC134407.1 | 0.501797002 | 1.16E-27 | positive |
| RASA2 | AC134407.1 | 0.411422454 | 2.92E-18 | positive |
| RNF146 | AC134407.1 | 0.425511595 | 1.51E-19 | positive |
| ROCK1 | AC134407.1 | 0.42430202 | 1.96E-19 | positive |
| AR | AC105942.1 | 0.636011926 | 4.36E-48 | positive |
| CAV1 | AC105942.1 | 0.409780729 | 4.09E-18 | positive |
| CDH1 | AC105942.1 | -0.419605236 | 5.31E-19 | negative |
| DIAPH1 | AC105942.1 | -0.453298026 | 2.86E-22 | negative |
| ITGB1 | AC105942.1 | 0.481695646 | 2.54E-25 | positive |
| MAPT | AC105942.1 | 0.558652695 | 3.46E-35 | positive |
| MYLK | AC105942.1 | 0.774200002 | 1.84E-83 | positive |
| PCDH7 | AC105942.1 | 0.542220189 | 7.30E-33 | positive |
| PRKAA2 | AC105942.1 | 0.64575907 | 5.56E-50 | positive |
| PRNP | AC105942.1 | 0.687550649 | 5.95E-59 | positive |
| RNF146 | AC105942.1 | 0.411667164 | 2.78E-18 | positive |
| ROCK1 | AC105942.1 | 0.401827612 | 2.03E-17 | positive |
| SRF | AC105942.1 | 0.659709836 | 8.13E-53 | positive |
| STMN2 | AC105942.1 | 0.452007601 | 3.88E-22 | positive |
| TUBA1A | AC105942.1 | 0.740030269 | 1.25E-72 | positive |
| VCL | AC105942.1 | 0.631573286 | 3.02E-47 | positive |
| SPRN | AC009032.1 | 0.430235219 | 5.41E-20 | positive |
| CALB2 | AC245041.1 | 0.589832641 | 5.71E-40 | positive |
| CDH3 | AC245041.1 | 0.471931289 | 3.06E-24 | positive |
| KRT7 | AC245041.1 | 0.405730561 | 9.30E-18 | positive |
| PIKFYVE | AC138956.2 | 0.457042225 | 1.18E-22 | positive |
| RASA2 | AC138956.2 | 0.495507042 | 6.52E-27 | positive |
| MLANA | AC004494.1 | 0.52212656 | 3.44E-30 | positive |
| RASA2 | AC004494.1 | 0.53706879 | 3.68E-32 | positive |
| MLANA | CFLAR-AS1 | 0.45792374 | 9.52E-23 | positive |
| RASA2 | CFLAR-AS1 | 0.434217855 | 2.25E-20 | positive |
| MLANA | AC024267.5 | 0.466868766 | 1.08E-23 | positive |
| AR | AC093702.1 | 0.578007848 | 4.28E-38 | positive |
| CAV1 | AC093702.1 | 0.405278597 | 1.02E-17 | positive |
| ITGB1 | AC093702.1 | 0.452224594 | 3.68E-22 | positive |
| MAPT | AC093702.1 | 0.449070881 | 7.71E-22 | positive |
| MYLK | AC093702.1 | 0.785018506 | 2.68E-87 | positive |
| PCDH7 | AC093702.1 | 0.439277388 | 7.26E-21 | positive |
| PRKAA2 | AC093702.1 | 0.654716771 | 8.75E-52 | positive |
| PRNP | AC093702.1 | 0.725141686 | 2.00E-68 | positive |
| SRF | AC093702.1 | 0.536344373 | 4.61E-32 | positive |
| TUBA1A | AC093702.1 | 0.757667204 | 5.39E-78 | positive |
| VCL | AC093702.1 | 0.663485179 | 1.31E-53 | positive |
| MLANA | RAD51-AS1 | 0.429355749 | 6.56E-20 | positive |
| RASA2 | RAD51-AS1 | 0.464667377 | 1.86E-23 | positive |
| MYL2 | LINC02345 | 0.470273101 | 4.64E-24 | positive |
| MLANA | AP002812.5 | 0.416516012 | 1.02E-18 | positive |
| RASA2 | AP002812.5 | 0.406555844 | 7.87E-18 | positive |
| UVRAG | AP002812.5 | 0.443504913 | 2.78E-21 | positive |
| MLANA | AC008569.2 | 0.492714619 | 1.38E-26 | positive |
| RASA2 | AC008569.2 | 0.479115505 | 4.94E-25 | positive |
| RNF146 | AC008569.2 | 0.417250155 | 8.72E-19 | positive |
| CALB2 | LINC00973 | 0.448945515 | 7.94E-22 | positive |
| KRT7 | LINC00973 | 0.46269115 | 3.01E-23 | positive |
| TM9SF4 | AL118506.1 | 0.469506942 | 5.62E-24 | positive |
| AR | AC009126.1 | 0.429995511 | 5.70E-20 | positive |
| MYLK | AC009126.1 | 0.493760238 | 1.05E-26 | positive |
| PRKAA2 | AC009126.1 | 0.424455196 | 1.89E-19 | positive |
| TUBA1A | AC009126.1 | 0.447720143 | 1.05E-21 | positive |
| MLANA | AC018410.2 | 0.469657024 | 5.41E-24 | positive |
| SPRN | AC018410.2 | 0.402909811 | 1.64E-17 | positive |
| RNF146 | AL513327.3 | 0.404277095 | 1.25E-17 | positive |
| AR | LINC01081 | 0.426303305 | 1.27E-19 | positive |
| MYLK | LINC01081 | 0.451154933 | 4.74E-22 | positive |
| PDPN | LINC01081 | 0.473543673 | 2.04E-24 | positive |
| PRND | LINC01081 | 0.422190566 | 3.07E-19 | positive |
| TUBA1A | LINC01081 | 0.489103876 | 3.63E-26 | positive |
| VCL | LINC01081 | 0.445555982 | 1.74E-21 | positive |
| RNF146 | AC107375.1 | 0.442432725 | 3.55E-21 | positive |
| ROCK1 | AC107375.1 | 0.43541099 | 1.73E-20 | positive |
| MLANA | AC079336.2 | 0.402281863 | 1.86E-17 | positive |
| MLANA | AL450344.3 | 0.403954996 | 1.33E-17 | positive |
| NTS | AC147651.3 | 0.569008226 | 1.02E-36 | positive |
| GZMB | MMP25-AS1 | 0.442282891 | 3.68E-21 | positive |
| CALB2 | MYO16-AS1 | 0.531550558 | 2.02E-31 | positive |
| CDH3 | MYO16-AS1 | 0.438938028 | 7.84E-21 | positive |
| RASA2 | AC008760.1 | 0.440034087 | 6.12E-21 | positive |
| RNF146 | AL020997.3 | 0.413736601 | 1.81E-18 | positive |
| RNF146 | AC010834.3 | 0.435929663 | 1.54E-20 | positive |
| ROCK1 | AC010834.3 | 0.401404662 | 2.21E-17 | positive |
| RASA2 | AC124319.2 | 0.422539208 | 2.85E-19 | positive |
| MLANA | AC090589.3 | 0.434886603 | 1.94E-20 | positive |
| RASA2 | AC090589.3 | 0.402520759 | 1.77E-17 | positive |
| MRTFA | AC090587.1 | 0.416517448 | 1.02E-18 | positive |
| TGFB1 | AC090587.1 | 0.421494906 | 3.56E-19 | positive |
| AR | SERTAD4-AS1 | 0.595529049 | 6.70E-41 | positive |
| CAV1 | SERTAD4-AS1 | 0.4428669 | 3.22E-21 | positive |
| ITGB1 | SERTAD4-AS1 | 0.500564624 | 1.63E-27 | positive |
| MAPT | SERTAD4-AS1 | 0.524402935 | 1.75E-30 | positive |
| MYLK | SERTAD4-AS1 | 0.831747692 | 7.66E-107 | positive |
| PCDH7 | SERTAD4-AS1 | 0.619664613 | 4.65E-45 | positive |
| PRKAA2 | SERTAD4-AS1 | 0.638309873 | 1.58E-48 | positive |
| PRNP | SERTAD4-AS1 | 0.710002932 | 1.99E-64 | positive |
| SRF | SERTAD4-AS1 | 0.603045403 | 3.71E-42 | positive |
| STMN2 | SERTAD4-AS1 | 0.404832127 | 1.11E-17 | positive |
| TUBA1A | SERTAD4-AS1 | 0.807995294 | 3.10E-96 | positive |
| VCL | SERTAD4-AS1 | 0.705108554 | 3.43E-63 | positive |
| PIKFYVE | AC138956.1 | 0.427531243 | 9.75E-20 | positive |
| RASA2 | AC138956.1 | 0.545378849 | 2.67E-33 | positive |
| MAD2L1 | AC100861.1 | 0.437856038 | 1.00E-20 | positive |
| KRT7 | NKILA | 0.493753194 | 1.05E-26 | positive |
| RASA2 | AC090739.1 | 0.428331689 | 8.19E-20 | positive |
| MLANA | AC010761.3 | 0.463198668 | 2.66E-23 | positive |
| PIKFYVE | AC010761.3 | 0.410463235 | 3.56E-18 | positive |
| RASA2 | AC010761.3 | 0.464002401 | 2.19E-23 | positive |
| TF | AC007497.1 | 0.490567517 | 2.46E-26 | positive |
| AR | LINC00899 | 0.43137029 | 4.22E-20 | positive |
| DIAPH1 | LINC00899 | -0.44860224 | 8.59E-22 | negative |
| MYLK | LINC00899 | 0.447248844 | 1.18E-21 | positive |
| PRKAA2 | LINC00899 | 0.480635011 | 3.34E-25 | positive |
| PRNP | LINC00899 | 0.45053482 | 5.48E-22 | positive |
| TUBA1A | LINC00899 | 0.451462426 | 4.41E-22 | positive |
| KIF2C | AC021016.2 | -0.40671473 | 7.62E-18 | negative |
| TUBA1A | AC021016.2 | 0.44872493 | 8.35E-22 | positive |
| VIM | AC021016.2 | 0.43494313 | 1.92E-20 | positive |
| MLANA | AL133243.3 | 0.418415466 | 6.83E-19 | positive |
| RASA2 | AL133243.3 | 0.452958775 | 3.10E-22 | positive |
| ROCK1 | AL133243.3 | 0.473448394 | 2.09E-24 | positive |
| MLANA | AC117500.2 | 0.542273156 | 7.18E-33 | positive |
| RASA2 | AC117500.2 | 0.48226468 | 2.19E-25 | positive |
| AR | AF111167.2 | 0.499305906 | 2.31E-27 | positive |
| MAPT | AF111167.2 | 0.423829882 | 2.16E-19 | positive |
| MYLK | AF111167.2 | 0.539686925 | 1.62E-32 | positive |
| PRKAA2 | AF111167.2 | 0.416217948 | 1.08E-18 | positive |
| PRNP | AF111167.2 | 0.415616766 | 1.23E-18 | positive |
| ROCK1 | AF111167.2 | 0.411569823 | 2.83E-18 | positive |
| SRF | AF111167.2 | 0.4321367 | 3.56E-20 | positive |
| TUBA1A | AF111167.2 | 0.442007427 | 3.92E-21 | positive |
| UVRAG | AF111167.2 | 0.425030994 | 1.67E-19 | positive |
| VCL | AF111167.2 | 0.433405822 | 2.69E-20 | positive |
| CDC20 | VPS9D1-AS1 | 0.469760288 | 5.27E-24 | positive |
| AR | AC025280.1 | 0.575072687 | 1.22E-37 | positive |
| MAPT | AC025280.1 | 0.439781545 | 6.48E-21 | positive |
| MYLK | AC025280.1 | 0.581998554 | 1.02E-38 | positive |
| PRKAA2 | AC025280.1 | 0.465530585 | 1.50E-23 | positive |
| PRNP | AC025280.1 | 0.516411238 | 1.84E-29 | positive |
| SRF | AC025280.1 | 0.449279575 | 7.34E-22 | positive |
| TUBA1A | AC025280.1 | 0.609869369 | 2.50E-43 | positive |
| VCL | AC025280.1 | 0.548486295 | 9.82E-34 | positive |
| VIM | AC025280.1 | 0.421676566 | 3.43E-19 | positive |
| MLANA | AC022973.2 | 0.467878919 | 8.42E-24 | positive |
| RNF146 | AC020907.4 | 0.408648753 | 5.15E-18 | positive |
| RASA2 | PSPC1-AS2 | 0.418980596 | 6.06E-19 | positive |
| MLANA | RHOA-IT1 | 0.441541711 | 4.35E-21 | positive |
| RASA2 | RHOA-IT1 | 0.402156618 | 1.90E-17 | positive |
| ROCK1 | RHOA-IT1 | 0.40417775 | 1.27E-17 | positive |
| RASA2 | AC090971.2 | 0.441305979 | 4.59E-21 | positive |
| MLANA | AC008750.5 | 0.41911622 | 5.89E-19 | positive |
| CDC20 | TMPO-AS1 | 0.402469239 | 1.79E-17 | positive |
| RASA2 | AC008115.3 | 0.419358485 | 5.60E-19 | positive |
| AR | AL513217.1 | 0.715835176 | 6.15E-66 | positive |
| CAV1 | AL513217.1 | 0.466077593 | 1.31E-23 | positive |
| ITGB1 | AL513217.1 | 0.477267103 | 7.93E-25 | positive |
| MAPT | AL513217.1 | 0.663964262 | 1.04E-53 | positive |
| MYLK | AL513217.1 | 0.882339095 | 2.77E-136 | positive |
| PCDH7 | AL513217.1 | 0.6119588 | 1.08E-43 | positive |
| PRKAA2 | AL513217.1 | 0.685809471 | 1.51E-58 | positive |
| PRNP | AL513217.1 | 0.719234363 | 7.79E-67 | positive |
| SRF | AL513217.1 | 0.73335588 | 1.04E-70 | positive |
| TUBA1A | AL513217.1 | 0.732642282 | 1.66E-70 | positive |
| VCL | AL513217.1 | 0.757747522 | 5.08E-78 | positive |
| RAC1 | AC087620.1 | 0.403996562 | 1.32E-17 | positive |
| AR | MIR1-1HG-AS1 | 0.776711188 | 2.47E-84 | positive |
| CAV1 | MIR1-1HG-AS1 | 0.498816893 | 2.64E-27 | positive |
| ITGB1 | MIR1-1HG-AS1 | 0.52694822 | 8.15E-31 | positive |
| MAPT | MIR1-1HG-AS1 | 0.736069732 | 1.75E-71 | positive |
| MYLK | MIR1-1HG-AS1 | 0.949495955 | 1.71E-208 | positive |
| PCDH7 | MIR1-1HG-AS1 | 0.6406408 | 5.60E-49 | positive |
| PRKAA2 | MIR1-1HG-AS1 | 0.752242885 | 2.69E-76 | positive |
| PRNP | MIR1-1HG-AS1 | 0.774790591 | 1.15E-83 | positive |
| RNF146 | MIR1-1HG-AS1 | 0.403105396 | 1.57E-17 | positive |
| ROCK1 | MIR1-1HG-AS1 | 0.41021703 | 3.74E-18 | positive |
| S100B | MIR1-1HG-AS1 | 0.405733834 | 9.29E-18 | positive |
| SRF | MIR1-1HG-AS1 | 0.759700787 | 1.21E-78 | positive |
| TUBA1A | MIR1-1HG-AS1 | 0.784388214 | 4.55E-87 | positive |
| VCL | MIR1-1HG-AS1 | 0.821128576 | 6.62E-102 | positive |
| MLANA | AC007347.1 | 0.419949446 | 4.94E-19 | positive |
| PRKAA2 | AC009812.3 | 0.431998885 | 3.67E-20 | positive |
| ROCK1 | AC009812.3 | 0.443230706 | 2.96E-21 | positive |
| AR | ZSCAN16-AS1 | 0.452019928 | 3.87E-22 | positive |
| DIAPH1 | ZSCAN16-AS1 | -0.413948015 | 1.73E-18 | negative |
| MAPT | ZSCAN16-AS1 | 0.464464662 | 1.95E-23 | positive |
| MYLK | ZSCAN16-AS1 | 0.500192985 | 1.81E-27 | positive |
| PRKAA2 | ZSCAN16-AS1 | 0.449487819 | 6.99E-22 | positive |
| PRNP | ZSCAN16-AS1 | 0.45677837 | 1.25E-22 | positive |
| TUBA1A | ZSCAN16-AS1 | 0.529515277 | 3.75E-31 | positive |
| MLANA | AC112512.1 | 0.40165854 | 2.10E-17 | positive |
| GZMB | AC090912.2 | 0.454996116 | 1.91E-22 | positive |
| MLANA | BACH1-IT1 | 0.453670829 | 2.62E-22 | positive |
| RASA2 | BACH1-IT1 | 0.423837426 | 2.16E-19 | positive |
| ROCK1 | BACH1-IT1 | 0.476639978 | 9.31E-25 | positive |
| CALB2 | AL161785.1 | 0.606202771 | 1.07E-42 | positive |
| RASA2 | AL355388.2 | 0.411231604 | 3.04E-18 | positive |
| LPAR2 | AL356740.3 | 0.412648995 | 2.27E-18 | positive |
| SOD1 | AL109811.2 | 0.438082078 | 9.51E-21 | positive |
| MLANA | AL080317.3 | 0.413325376 | 1.97E-18 | positive |
| SPRN | AL035587.1 | 0.401581716 | 2.13E-17 | positive |
| RASA2 | AC000123.1 | 0.430418261 | 5.20E-20 | positive |
| MLANA | AC025171.2 | 0.41563825 | 1.22E-18 | positive |
| ERI3 | PITPNA-AS1 | 0.407234463 | 6.86E-18 | positive |
