# Supplementary material for: Cell-in-cell associated lncRNA signature predicts prognosis and immunotherapy response in gastric cancer
Source: Front Oncol. 2025 Jul 8;15:1597187. doi: 10.3389/fonc.2025.1597187 (PMC12280372; doi:10.3389/fonc.2025.1597187)
Supplement: Supplementary file 2 [file Table2.docx]

**Table S2.** The CICRlncRNAs associated with OS in GC.

| **LncRNA** | **HR** | **HR.95L** | **HR.95H** | ***P*** |
| --- | --- | --- | --- | --- |
| AF131216.4 | 1.618002655 | 1.139064106 | 2.298318926 | 0.007208351 |
| BOLA3-AS1 | 1.286695058 | 1.076607796 | 1.53777836 | 0.005578982 |
| AC245041.2 | 1.070962268 | 1.0110373 | 1.134439035 | 0.019616708 |
| PVT1 | 0.672119709 | 0.500663217 | 0.902292975 | 0.008187913 |
| AP001922.6 | 1.976696437 | 1.109659954 | 3.521194749 | 0.02071238 |
| AP001528.2 | 1.318395786 | 1.054213358 | 1.648781469 | 0.015405907 |
| NR2F1-AS1 | 1.836496251 | 1.05676971 | 3.191535913 | 0.031099906 |
| LINC01978 | 0.776203498 | 0.642220874 | 0.9381381 | 0.008780144 |
| LINC01579 | 1.370198683 | 1.039214568 | 1.806599415 | 0.025573154 |
| AC007541.1 | 2.025973874 | 1.238127328 | 3.315143802 | 0.004952659 |
| AL353804.2 | 0.595286106 | 0.356129172 | 0.995047796 | 0.047826849 |
| NR2F2-AS1 | 1.980591471 | 1.061005851 | 3.697192217 | 0.031880098 |
| AP003392.1 | 0.846294858 | 0.720067422 | 0.994649896 | 0.04286142 |
| AC037198.1 | 1.161087411 | 1.002751442 | 1.344424869 | 0.045858169 |
| AC084033.3 | 1.58349328 | 1.081976644 | 2.317472362 | 0.018008436 |
| AP000695.2 | 1.245131818 | 1.06573033 | 1.454733153 | 0.005745945 |
| AC245041.1 | 1.029285618 | 1.013912931 | 1.044891381 | 0.000170183 |
| AL161785.1 | 1.109293407 | 1.056286131 | 1.164960731 | 3.30E-05 |
