# Supplementary material for: Cell-in-cell associated lncRNA signature predicts prognosis and immunotherapy response in gastric cancer
Source: Front Oncol. 2025 Jul 8;15:1597187. doi: 10.3389/fonc.2025.1597187 (PMC12280372; doi:10.3389/fonc.2025.1597187)
Supplement: Supplementary file 3 [file Table3.docx]

**Table S3.** The differentially expressed genes (DEGs) between the risk groups.

| **Gene** | **LowMean** | **HighMean** | **LogFC** | ***P*** | **FDR** |
| --- | --- | --- | --- | --- | --- |
| LINC00926 | 2.022872527 | 0.87596 | -1.207468514 | 0.00175115 | 0.004726257 |
| MMP11 | 18.73949231 | 44.95121676 | 1.2622783 | 4.21E-08 | 4.38E-07 |
| WNT2 | 1.132964286 | 2.951715135 | 1.381451112 | 1.95E-15 | 2.13E-13 |
| C1QC | 93.99286429 | 207.4273162 | 1.141982756 | 5.20E-17 | 1.10E-14 |
| CPE | 8.836382967 | 18.30894865 | 1.051021099 | 4.69E-06 | 2.83E-05 |
| MIR5094 | 2.5377 | 1.169991892 | -1.117022996 | 7.49E-17 | 1.53E-14 |
| AC136428.1 | 1.42459011 | 2.914423784 | 1.032663794 | 0.00039911 | 0.001331202 |
| CCL18 | 19.02676593 | 44.02374054 | 1.21025137 | 2.01E-08 | 2.31E-07 |
| MTND2P13 | 14.89135824 | 0.559395135 | -4.734463831 | 1.70E-05 | 8.72E-05 |
| IGKV2-24 | 25.49463681 | 55.63794486 | 1.125875345 | 6.08E-05 | 0.000264325 |
| LAMP5 | 1.291021429 | 2.770998919 | 1.101893201 | 6.13E-09 | 7.96E-08 |
| LCN12 | 1.521439011 | 0.557842162 | -1.447507619 | 1.46E-10 | 2.99E-09 |
| POSTN | 36.3385478 | 76.62201351 | 1.076258172 | 5.93E-12 | 1.79E-10 |
| NPIPB15 | 3.513830769 | 1.691839459 | -1.054452035 | 1.84E-07 | 1.60E-06 |
| IGKV2D-28 | 3.772052747 | 8.112406486 | 1.104780093 | 0.00078271 | 0.002380674 |
| RNU7-45P | 9.685308242 | 4.634364324 | -1.0634265 | 2.11E-08 | 2.40E-07 |
| SERPINF1 | 24.1241467 | 50.28115946 | 1.059540004 | 4.93E-14 | 3.16E-12 |
| THBS2 | 10.42580824 | 22.83261405 | 1.13093681 | 5.92E-13 | 2.59E-11 |
| MIR320D1 | 3.417071429 | 1.470245946 | -1.216702893 | 1.23E-05 | 6.58E-05 |
| ANKRD36C | 2.362642308 | 1.058061081 | -1.158978313 | 1.64E-10 | 3.32E-09 |
| CRLF1 | 1.104114835 | 2.397049189 | 1.118369285 | 0.010596091 | 0.022206027 |
| MIR6087 | 56.63526319 | 144.143393 | 1.347732198 | 9.93E-07 | 7.24E-06 |
| DPEP3 | 0.596095055 | 1.654605405 | 1.47287289 | 0.000712771 | 0.002196126 |
| OLFML2B | 10.26895 | 23.68832054 | 1.205887244 | 2.37E-16 | 3.75E-14 |
| RNA5SP202 | 0.745884066 | 2.363960541 | 1.664182642 | 0.01339378 | 0.027160756 |
| COL10A1 | 7.651476923 | 21.5111573 | 1.491274988 | 2.81E-14 | 1.98E-12 |
| IGHV1-3 | 52.91602802 | 136.0982151 | 1.362871467 | 0.000121802 | 0.000480379 |
| CSF1R | 8.252874725 | 16.60336324 | 1.008506863 | 4.58E-15 | 4.14E-13 |
| FGL1 | 0.97931044 | 2.964265946 | 1.597836719 | 0.003712936 | 0.009042588 |
| MTATP8P2 | 24.97988407 | 12.16089892 | -1.038516906 | 9.80E-05 | 0.000397878 |
| SFRP2 | 51.94480495 | 149.7146768 | 1.527164282 | 3.93E-11 | 9.49E-10 |
| IGHV1-69 | 23.79487857 | 53.53126541 | 1.169730664 | 0.004351754 | 0.010368618 |
| HBB | 14.19577582 | 40.10157405 | 1.498197169 | 0.020605992 | 0.039014051 |
| TYROBP | 58.82812912 | 118.1669795 | 1.006248885 | 6.07E-16 | 8.15E-14 |
| IGKV2-28 | 3.343709341 | 8.0959 | 1.275742027 | 0.000518192 | 0.001668529 |
| CHIT1 | 1.002530769 | 2.073301081 | 1.048283121 | 0.000187562 | 0.000698301 |
| COL11A2 | 2.888346154 | 0.53741027 | -2.426147855 | 0.000382797 | 0.001283243 |
| CTHRC1 | 17.51242198 | 41.96552919 | 1.26082615 | 2.28E-17 | 5.46E-15 |
| AC080129.2 | 1.376811538 | 0.644110811 | -1.095950282 | 6.34E-10 | 1.09E-08 |
| IGKV1D-42 | 0.696667582 | 1.901701622 | 1.448748566 | 0.002108644 | 0.005562386 |
| IGLV3-9 | 15.31115934 | 45.31278595 | 1.565334668 | 0.000274975 | 0.000969962 |
| IGHV3-43 | 12.33653077 | 27.13945027 | 1.137454756 | 0.007938334 | 0.017360402 |
| TMPRSS5 | 1.423214835 | 0.660904865 | -1.106638934 | 0.001665167 | 0.004536556 |
| ISLR | 26.54951264 | 59.40516054 | 1.161902886 | 1.84E-13 | 9.35E-12 |
| TREM2 | 6.316299451 | 14.91450054 | 1.23956419 | 3.17E-16 | 4.90E-14 |
| FPR1 | 2.224448901 | 5.124015135 | 1.203826779 | 8.18E-14 | 4.99E-12 |
| CST6 | 1.935626923 | 12.34549838 | 2.673112262 | 0.001715013 | 0.004641916 |
| AC010320.3 | 1.444427473 | 0.708430811 | -1.027798902 | 3.40E-11 | 8.39E-10 |
| CAPN14 | 2.630257692 | 0.957743784 | -1.457492489 | 0.007709155 | 0.016932632 |
| IGLV5-48 | 0.724758791 | 2.052693514 | 1.501945402 | 0.002731034 | 0.006961101 |
| RNU6-130P | 2.220481319 | 1.024070811 | -1.116556958 | 1.06E-10 | 2.23E-09 |
| AL139393.2 | 0.918116484 | 1.932934595 | 1.074043713 | 1.39E-12 | 5.09E-11 |
| CD14 | 31.42821264 | 63.29599351 | 1.010053957 | 3.09E-18 | 1.06E-15 |
| GALNT15 | 0.678696154 | 1.408968649 | 1.053801768 | 1.34E-09 | 2.11E-08 |
| BIRC7 | 0.694404945 | 1.565791351 | 1.173042853 | 0.002571122 | 0.006605677 |
| C1QB | 98.37137308 | 228.4943395 | 1.215847981 | 1.77E-16 | 2.95E-14 |
| BGN | 186.1652374 | 376.0098584 | 1.014186784 | 1.15E-14 | 9.22E-13 |
| VGLL3 | 0.908196703 | 1.939292973 | 1.094454066 | 4.48E-10 | 8.17E-09 |
| IGHV3-66 | 7.962154945 | 17.00966486 | 1.095123864 | 0.011400591 | 0.023631253 |
| IGLV2-8 | 45.41496154 | 106.0408768 | 1.22338094 | 4.33E-05 | 0.000197061 |
| AL691482.3 | 2.421652747 | 1.155797297 | -1.067103603 | 5.74E-12 | 1.74E-10 |
| IGKV3-11 | 177.7853615 | 442.2002919 | 1.314563437 | 3.73E-06 | 2.32E-05 |
| AC078962.1 | 2.274921978 | 1.077996216 | -1.077464952 | 2.17E-09 | 3.21E-08 |
| MARCO | 1.066762088 | 3.288850811 | 1.624345108 | 5.92E-11 | 1.34E-09 |
| ZNF683 | 1.253948352 | 2.720125946 | 1.117195525 | 0.000104725 | 0.000421404 |
| IGKV2OR22-4 | 1.076163187 | 2.72349027 | 1.339559852 | 0.000289862 | 0.001015738 |
| LINC01503 | 0.687484066 | 1.382879459 | 1.008277226 | 1.38E-08 | 1.65E-07 |
| IGLV5-45 | 11.8276033 | 25.94656324 | 1.133385698 | 0.000682089 | 0.002113925 |
| IGLV3-27 | 16.1989467 | 33.62247676 | 1.053525994 | 0.009360235 | 0.019959112 |
| AC010970.1 | 21.75828846 | 45.45321622 | 1.062817305 | 4.10E-06 | 2.52E-05 |
| CILP | 2.269525824 | 5.05368973 | 1.154946189 | 2.37E-06 | 1.56E-05 |
| IGLV7-43 | 21.30439945 | 44.8642227 | 1.074414032 | 0.000182492 | 0.000681717 |
| CALB2 | 1.882693956 | 3.877177838 | 1.04220841 | 0.000304775 | 0.001059078 |
| AL158825.2 | 1.806791209 | 0.845621081 | -1.095346551 | 8.01E-13 | 3.19E-11 |
| ELANE | 0.178812637 | 7.813895135 | 5.449521288 | 0.025525821 | 0.046699572 |
| AC078883.2 | 1.57153022 | 0.727418378 | -1.111312733 | 5.43E-07 | 4.24E-06 |
| LINC01614 | 0.832037363 | 2.500228108 | 1.587339506 | 2.08E-15 | 2.18E-13 |
| IGKV3D-20 | 22.5680456 | 52.5908027 | 1.220529031 | 1.35E-05 | 7.14E-05 |
| IGHV1OR15-9 | 1.17442033 | 2.863711351 | 1.285937236 | 0.000301289 | 0.001049553 |
| LILRB4 | 2.345864835 | 5.001434595 | 1.092222082 | 1.86E-14 | 1.40E-12 |
| CLC | 0.774917033 | 2.496207568 | 1.687624143 | 0.000940037 | 0.002789684 |
| COL11A1 | 2.172038462 | 5.690627568 | 1.389538113 | 2.57E-10 | 4.99E-09 |
| MPO | 0.062465385 | 2.392085946 | 5.259068478 | 6.15E-05 | 0.000267256 |
| CGB5 | 0.246085165 | 3.676337297 | 3.901039544 | 0.004597304 | 0.010879523 |
| RNU7-41P | 7.015528022 | 3.415939459 | -1.038269286 | 1.09E-05 | 5.93E-05 |
| LINC00106 | 1.486631319 | 0.697604595 | -1.091565459 | 1.24E-21 | 2.74E-18 |
| ADAMTS2 | 6.894224725 | 14.43668541 | 1.066279313 | 8.44E-16 | 1.05E-13 |
| IGHV3-53 | 18.40238297 | 38.44802757 | 1.063016992 | 0.000137319 | 0.000533662 |
| AL117382.1 | 3.110474176 | 1.393470811 | -1.158451746 | 5.49E-10 | 9.71E-09 |
| IGHV4-4 | 25.3817544 | 78.08509892 | 1.621255471 | 1.17E-05 | 6.30E-05 |
| ITGA11 | 3.377925824 | 7.084842703 | 1.068598174 | 1.54E-13 | 8.15E-12 |
| FCGR1A | 1.015623077 | 2.102725946 | 1.049895751 | 3.87E-14 | 2.62E-12 |
| IGHV5-10-1 | 27.39253407 | 74.96995351 | 1.452529773 | 0.000115797 | 0.000459833 |
| FAP | 1.546352747 | 3.313817297 | 1.099624606 | 7.54E-16 | 9.55E-14 |
| IGKV1-39 | 12.59125 | 25.30553081 | 1.007031223 | 0.00010882 | 0.000436049 |
| MTND4P35 | 10.88506044 | 2.306467568 | -2.238592507 | 1.94E-08 | 2.24E-07 |
| FPR3 | 7.20576978 | 14.72016432 | 1.030569312 | 5.33E-14 | 3.39E-12 |
| IGHV3-7 | 106.3050648 | 229.5458124 | 1.110571779 | 2.37E-05 | 0.000116223 |
| RNU6-850P | 5.172086813 | 2.431138919 | -1.089114157 | 1.30E-18 | 5.75E-16 |
| IGHV3OR16-8 | 1.408401099 | 3.016627568 | 1.098878335 | 3.95E-05 | 0.000182531 |
| MIR647 | 18.98278242 | 9.364011351 | -1.019492884 | 1.15E-20 | 1.39E-17 |
| NTM | 0.861062637 | 1.957166486 | 1.18457639 | 1.12E-14 | 9.05E-13 |
| TREM1 | 0.780098901 | 1.721355135 | 1.141815827 | 4.57E-11 | 1.08E-09 |
| GAS1 | 4.751828571 | 9.906829189 | 1.059940588 | 8.54E-11 | 1.83E-09 |
| FP236383.3 | 3.169022527 | 8.033478378 | 1.341986873 | 1.08E-05 | 5.91E-05 |
| PTMAP1 | 2.264687912 | 1.041281081 | -1.120952693 | 2.81E-17 | 6.56E-15 |
| AC005086.2 | 1.419201648 | 0.685975676 | -1.048850265 | 3.01E-09 | 4.29E-08 |
| SPP1 | 39.54801374 | 118.3187541 | 1.581001622 | 1.69E-09 | 2.57E-08 |
| SIGLEC7 | 0.666397802 | 1.457078378 | 1.128622936 | 2.21E-16 | 3.54E-14 |
| AL355312.3 | 1.543001099 | 3.154417297 | 1.031634437 | 0.014319936 | 0.028724133 |
| PILRA | 2.940682418 | 6.386410811 | 1.118854371 | 8.18E-19 | 4.03E-16 |
| AC007849.1 | 3.051046703 | 1.413217838 | -1.110320399 | 4.33E-08 | 4.51E-07 |
| FP236383.2 | 2.170826923 | 4.760669189 | 1.132919675 | 0.000400776 | 0.001335418 |
| IGLV1-41 | 1.588495055 | 6.050099459 | 1.929298261 | 0.000116019 | 0.000460578 |
| AD000090.1 | 19.52585659 | 44.69100162 | 1.194598539 | 4.77E-05 | 0.000214399 |
| AC016542.2 | 3.393454945 | 1.49762973 | -1.180073879 | 2.04E-08 | 2.33E-07 |
| LINC00342 | 6.541625824 | 2.459357838 | -1.411367578 | 1.31E-11 | 3.73E-10 |
| PAGE1 | 2.565987363 | 0.455612973 | -2.493633334 | 0.000529774 | 0.001700059 |
| SPARC | 168.6693104 | 337.95978 | 1.002654067 | 8.34E-18 | 2.29E-15 |
| CST2 | 4.544943956 | 9.751700541 | 1.101391319 | 7.16E-10 | 1.22E-08 |
| C1QA | 74.93899341 | 155.6817054 | 1.054810915 | 1.46E-14 | 1.14E-12 |
| C3AR1 | 3.819245055 | 7.656827027 | 1.003459175 | 2.53E-14 | 1.81E-12 |
| IGLV6-57 | 77.93356099 | 229.5973211 | 1.558789165 | 9.64E-05 | 0.000392424 |
| VSIG4 | 5.180248352 | 12.21291189 | 1.237314049 | 1.97E-15 | 2.13E-13 |
| IGHV1-69D | 53.66933516 | 172.9040184 | 1.687801477 | 7.15E-05 | 0.000304972 |
| S100A12 | 3.235441209 | 6.690534054 | 1.048158913 | 0.002953703 | 0.00744871 |
| OSM | 1.296589011 | 3.356907568 | 1.372411562 | 4.44E-11 | 1.06E-09 |
| AL359075.1 | 1.920110989 | 0.948217838 | -1.017899268 | 3.27E-09 | 4.58E-08 |
| TLR8 | 0.772625824 | 1.585030811 | 1.036669081 | 3.57E-09 | 4.96E-08 |
| SNORA11 | 0.724887363 | 1.722573514 | 1.24873681 | 0.000767831 | 0.0023462 |
| IGLV9-49 | 8.382413187 | 18.54095838 | 1.145278277 | 0.000266496 | 0.000944315 |
| OBP2B | 2.766025275 | 0.128285946 | -4.430379306 | 0.014896697 | 0.029657321 |
| MATN3 | 1.411905495 | 3.101743243 | 1.135435742 | 3.65E-09 | 5.06E-08 |
| SFRP4 | 19.1165978 | 49.20415081 | 1.363954236 | 4.66E-11 | 1.10E-09 |
| MFAP5 | 4.570412637 | 9.178875676 | 1.005993024 | 1.77E-09 | 2.68E-08 |
| GPNMB | 19.26590659 | 42.18346216 | 1.130627431 | 2.05E-15 | 2.16E-13 |
| AC002553.1 | 2.441262637 | 1.196067027 | -1.029329273 | 4.50E-20 | 3.99E-17 |
| OSCAR | 1.850313187 | 3.931756216 | 1.087404388 | 3.41E-16 | 5.10E-14 |
| MTCO1P42 | 3.826065385 | 0.257058378 | -3.895693588 | 0.000533885 | 0.001710315 |
| IGHV3-11 | 36.05917582 | 77.88160486 | 1.110916191 | 4.42E-06 | 2.69E-05 |
| AC122718.1 | 2.251738462 | 1.124902703 | -1.001239046 | 8.84E-09 | 1.11E-07 |
| SLC17A4 | 1.724006044 | 0.78407027 | -1.136709969 | 0.003182354 | 0.007921623 |
| HTR2B | 0.664996154 | 1.405042162 | 1.079195522 | 6.22E-05 | 0.000269743 |
| HSD11B1 | 1.888994505 | 4.002203784 | 1.083176123 | 5.59E-13 | 2.48E-11 |
| RNASE2 | 1.285271978 | 3.283901622 | 1.353337226 | 3.84E-16 | 5.61E-14 |
| IGKV2-30 | 41.88841538 | 89.12470378 | 1.089274068 | 0.001599204 | 0.00438651 |
| IGHV1-18 | 137.7922346 | 295.1531335 | 1.098969072 | 0.002316588 | 0.00602485 |
| IGLC7 | 17.2106467 | 39.86714973 | 1.211899156 | 0.013265363 | 0.026937128 |
| ADORA3 | 0.731745055 | 1.514988649 | 1.049893988 | 4.70E-13 | 2.12E-11 |
| HTRA3 | 14.96999725 | 34.51611135 | 1.20519598 | 5.73E-16 | 7.78E-14 |
| NNAT | 5.937274176 | 2.573668649 | -1.205974413 | 0.000659181 | 0.002056749 |
| CCL23 | 0.70362967 | 2.754977297 | 1.969152206 | 7.14E-07 | 5.43E-06 |
| IGKV1D-16 | 7.019552198 | 22.29043838 | 1.666974086 | 0.019653117 | 0.0375263 |
| ATP6V1C2 | 2.334000549 | 1.08704973 | -1.102386959 | 8.00E-07 | 5.96E-06 |
| SHISA2 | 1.904421429 | 4.05001027 | 1.088572799 | 6.74E-06 | 3.86E-05 |
| CCL8 | 2.674012088 | 5.563442703 | 1.056971923 | 2.21E-06 | 1.47E-05 |
| KRT81 | 0.32007967 | 2.831016216 | 3.14481706 | 0.000193508 | 0.000717224 |
| CD177 | 5.931524176 | 20.58030324 | 1.794789465 | 0.001710532 | 0.004632622 |
| AC009961.2 | 1.687658791 | 0.626641081 | -1.429311994 | 4.01E-11 | 9.65E-10 |
| ISX | 2.031413736 | 0.991172432 | -1.035276135 | 0.012198767 | 0.025035907 |
| FNDC1 | 8.405776923 | 19.08032757 | 1.182632864 | 3.85E-12 | 1.23E-10 |
| APOE | 91.77122967 | 210.9945946 | 1.201092195 | 1.30E-13 | 7.18E-12 |
| IGHV4-31 | 26.7339978 | 54.25986216 | 1.021209788 | 7.66E-05 | 0.000324515 |
| ITGBL1 | 1.268854396 | 2.849542703 | 1.167203887 | 2.31E-12 | 7.92E-11 |
| BCAT1 | 2.147069231 | 4.562574595 | 1.087479435 | 4.34E-07 | 3.47E-06 |
| DERL3 | 1.315885165 | 2.640132973 | 1.004577001 | 2.47E-06 | 1.62E-05 |
| ADAM12 | 1.826703846 | 3.993731351 | 1.128494533 | 2.11E-11 | 5.53E-10 |
| KRT40 | 1.824264286 | 0.267085946 | -2.771938783 | 0.02016648 | 0.03830738 |
| APOC1 | 18.58487857 | 40.70389189 | 1.131037482 | 3.11E-10 | 5.86E-09 |
| LRRC15 | 2.000230769 | 5.492074054 | 1.457184623 | 4.42E-11 | 1.06E-09 |
| MIR320E | 9.176067033 | 4.586523784 | -1.00047481 | 4.37E-13 | 1.99E-11 |
| CST1 | 54.17234231 | 140.9499362 | 1.379554448 | 2.12E-06 | 1.42E-05 |
| AQP9 | 1.419593407 | 3.219196216 | 1.181222736 | 9.76E-10 | 1.62E-08 |
| SLAMF8 | 1.204151099 | 2.698798919 | 1.164301054 | 2.75E-13 | 1.33E-11 |
| PDGFRL | 1.508402198 | 3.251175676 | 1.107940356 | 1.34E-09 | 2.11E-08 |
| IGLV7-46 | 36.68037473 | 80.61170973 | 1.135981044 | 0.00663398 | 0.014901106 |
| CLEC4E | 1.056752198 | 2.163223243 | 1.033545445 | 2.41E-08 | 2.68E-07 |
| FCGR3A | 13.34576484 | 28.51560162 | 1.095369483 | 6.77E-15 | 5.66E-13 |
| LINC02474 | 1.113614835 | 2.352078919 | 1.078686132 | 0.004559487 | 0.010797717 |
| MTND1P23 | 442.3712192 | 147.8125984 | -1.581488288 | 2.19E-06 | 1.46E-05 |
| IGHV2-26 | 23.32156868 | 49.16023243 | 1.075826905 | 0.006793436 | 0.015200055 |
| OLR1 | 2.024103297 | 5.338782162 | 1.399227767 | 3.64E-17 | 8.20E-15 |
| MTND4P24 | 28.68445495 | 10.88324649 | -1.398160126 | 2.25E-11 | 5.88E-10 |
